# Hnrnpul1 controls transcription, splicing, and modulates skeletal and limb development in vivo

**DOI:** 10.1093/g3journal/jkac067

**Published:** 2022-03-23

**Authors:** Danielle L Blackwell, Sherri D Fraser, Oana Caluseriu, Claudia Vivori, Amanda V Tyndall, Ryan E Lamont, Jillian S Parboosingh, A Micheil Innes, François P Bernier, Sarah J Childs

**Affiliations:** 1 Department of Biochemistry and Molecular Biology, University of Calgary, Calgary, AB T2N 4N1, Canada; 2 Alberta Children’s Hospital Research Institute, University of Calgary, Calgary, AB T2N 4N1, Canada; 3 Department of Medical Genetics, University of Alberta, Edmonton, AB T6G 2R3, Canada; 4 Centre for Genomic Regulation (CRG), The Barcelona Institute of Science and Technology, Barcelona 08003, Spain; 5 Universitat Pompeu Fabra (UPF), Barcelona 08002, Spain; 6 Department of Medical Genetics, University of Calgary, Calgary, AB T2N 4N1, Canada

**Keywords:** Hnrnpul1, alternative splicing, zebrafish, limb, craniofacial, scoliosis

## Abstract

Mutations in RNA-binding proteins can lead to pleiotropic phenotypes including craniofacial, skeletal, limb, and neurological symptoms. Heterogeneous nuclear ribonucleoproteins (hnRNPs) are involved in nucleic acid binding, transcription, and splicing through direct binding to DNA and RNA, or through interaction with other proteins in the spliceosome. We show a developmental role for Hnrnpul1 in zebrafish, resulting in reduced body and fin growth and missing bones. Defects in craniofacial tendon growth and adult-onset caudal scoliosis are also seen. We demonstrate a role for Hnrnpul1 in alternative splicing and transcriptional regulation using RNA-sequencing, particularly of genes involved in translation, ubiquitination, and DNA damage. Given its cross-species conservation and role in splicing, it would not be surprising if it had a role in human development. Whole-exome sequencing detected a homozygous frameshift variant in *HNRNPUL1* in 2 siblings with congenital limb malformations, which is a candidate gene for their limb malformations. Zebrafish Hnrnpul1 mutants suggest an important developmental role of hnRNPUL1 and provide motivation for exploring the potential conservation of ancient regulatory circuits involving hnRNPUL1 in human development.

## Introduction

Mutations in proteins involved in alternative splicing (AS) lead to spliceosomopathies in humans. Despite being expressed ubiquitously, mutations in core and AS factors can result in tissue-restricted, cell-type-specific phenotypes including craniofacial, limb, skeletal, and neurological syndromes (Lehalle *et al.* 2015). Tissue-specificity can occur because of the sensitivity of individual tissues during embryonic development to AS, for example neural crest is sensitive to AS in the case of craniofacial anomalies (Lehalle *et al.* 2015). AS is the process of producing multiple different mRNA transcripts and protein isoforms through the differential selection of splice sites within a single pre-mRNA molecule. AS of pre-mRNA is carried out by the spliceosome, which is a complex of small nuclear RNAs and proteins. AS events include exon skipping, intron retention, and use of alternative 5′ or 3′ splice sites. For example, pathogenic variants in *TXNL4A* (Wieczorek *et al.* 2014), *EIF4A3* (Favaro *et al.* 2014), *EFTUD2* (Lines *et al.* 2012), *SF3B4* (Bernier *et al.* 2012), and *SNRPB* (Lynch *et al.* 2014) cause human spliceosomopathies (Lehalle *et al.* 2015).

Members of the heterogeneous nuclear ribonucleoprotein (hnRNP) family are involved in nucleic acid binding, splicing, and transcription. They are present in the spliceosome and contribute directly and indirectly to the processing of pre-mRNA into mature mRNA, with nearly all hnRNP proteins having RNA-binding motifs (Dreyfuss *et al.* 1993; Geuens *et al.* 2016). Pathogenic variants associated with human disease have been discovered in hnRNP family members *HNRNPK* (Au *et al.* 2015), *HNRNPU* (Poot 2019), *HNRNPDL* (Batlle *et al.* 2020), and HNRNP’s in general (Gillentine *et al.* 2021). In particular, members of the hnRNPU family often act as repressors of mRNA splicing (Matlin *et al.* 2005). hnRNPU proteins are involved in DNA repair (Hegde *et al.* 2012; Polo *et al.* 2012) and U2 snRNP maturation (Xiao *et al.* 2012). hnRNPUL1 (also known as E1B-AP5) is a transcriptional repressor (Kzhyshkowska *et al.* 2003).

We studied the developmental role of hnRNPUL1 in zebrafish to define its in vivo function. Zebrafish are an ideal model for this study due to the conservation of developmental processes and genetic networks with human, coupled with rapid development. We identified a multitissue phenotype involving fin, craniofacial, and skeletal abnormalities including scoliosis. Through RNA-sequencing (RNAseq) and AS analysis, we found that alternative splicing events are disrupted in zebrafish *hnrnpul1* mutants. We detected a homozygous frameshift variant in Heterogeneous Nuclear Ribonucleoprotein U Like 1 (*HNRNPUL1*) gene in 2 siblings with craniofacial and limb anomalies in humans, raising the possibility that *HNRNPUL1* is critical to vertebrate development.

## Materials and methods

### Animal and patient data

All zebrafish (*Danio rerio*) strains were maintained and raised under established protocols (Westerfield 2000) and all animal procedures were approved by the University of Calgary Animal Care Committee (protocol AC17-0189). Zebrafish embryos were collected and incubated at 28.5°C in E3 medium (5 mM NaCl, 170 µM KCl, 330 µM CaCl_2_, 300 µM MgSO_4_, and 223 µM Methylene blue) and staged in hours postfertilization (hpf) or days post fertilization (dpf). When required, endogenous pigmentation was inhibited from 24 hpf by the addition of 0.003% 1-phenyl-2-thiourea (PTU, Sigma-Aldrich) in E3 medium.

This study was part of the Finding of Rare Disease Genes in Canada (FORGE Canada) consortium and approved by the University of Calgary Conjoint Health Research Ethics Board (REB# 23927).

DNA was extracted from all family members from whole blood using Puregene Chemistry (Qiagen). Exome capture was undertaken in both affected individuals using the SureSelect 50 Mb All Exon Kit V3 (Agilent) followed by sequencing with an HiSeq2000 (Illumina). Variant calling and annotation were as described in Lynch *et al.* (2014). Confirmation and segregation of the *HNRNPUL1* c.1406dup variant were performed by PCR amplification (HotStar Taq Plus, Qiagen, Toronto, ON, USA) from genomic DNA, and Sanger sequencing with the ABI BigDye Terminator Cycle Sequencing Kit v1.1 (Life Technologies, Burlington, ON, USA) on a 3130xl genetic analyzer (Life Technologies). Sequence subtraction and analysis were performed using Mutation Surveyor software (SoftGenetics, State College, PA, USA).

### Generation of *hnrnpul1* and *hnrnpul1l* mutant zebrafish

CRISPR mutations were created in both the *hnrnpul1* and *hnrnpul1l* genes by injection of guide RNAs following the protocol of Gagnon *et al.* (2014). Cas9 was transcribed from pCS2-Cas9 (Addgene 47322) and injected into embryos at the single-cell stage along with in vitro transcribed guides (mMessage Machine T7 transcription kit, ThermoFisher), and a homology-directed repair STOP cassette sequence oligonucleotide (Integrated DNA Technologies, Sequences in Supplementary Table 4). Guide RNAs were designed to target a location close to the human mutation using CHOPCHOP (Labun *et al.* 2019). Founders were identified by genomic PCR analysis using primers in Supplementary Table 4. DNA from F1 heterozygotes was cloned into pCRBlunt II-TOPO vector (Thermofisher) and sequenced. Mutants were genotyped by PCR using primers described in Supplementary Table 4, detailed protocol in Supplementary Methods.

### Whole-mount in situ hybridization

Embryos were fixed in 4% paraformaldehyde in PBS with 0.1% Tween-20 (PFA) at 4°C overnight and stored in 100% methanol at −20°C until required. All whole-mount in situ hybridization (WISH) was carried out according to standard protocols (Lauter *et al.* 2011). Antisense probes for *hnrnpul1*, *hnrnpul1l*, *scxa*, *hand2, tbx5, foxd3*, and *sox10* were produced by in vitro transcription using T7 polymerase (Roche), in the presence of digoxigenin-11-UTP (Sigma), from PCR fragments amplified from embryonic zebrafish cDNA (Supplementary Table 4). Antisense probes for *gli3* and *col1a1a* were a gift from Peng Huang and produced from plasmid clones. Images were taken on a Zeiss Stemi SV 11 microscope with a Zeiss Axiocam HRc camera. Area and length measurements were completed in ImageJ using the line and measure tools or Zen Blue (Zeiss) using the line tool. Confocal images were collected on a Zeiss LSM700 confocal microscope and assembled in ImageJ and Adobe Photoshop.

### Alcian blue and alizarin red staining

Alcian blue staining of 16 dpf zebrafish was carried out as previously described (Walker and Kimmel 2007). In brief, fish were fixed in 4% PFA overnight at 4°C and stained in 0.04% Alcian blue in 100 mM Tris-HCl/10 mM MgCl_2._ Following staining, fish were washed in decreasing concentrations of Ethanol/100 mM Tris-HCl to remove excess stain. Fish were bleached in 3% H_2_0_2_/0.5% KOH until pigment was lost. Fish were then washed in increasing concentrations of glycerol in 1% KOH until 100% glycerol. Fish were imaged in 100% glycerol.

Alizarin red staining of larval and adult zebrafish was carried out as previously described (Connolly and Yelick 2010). In brief, adult zebrafish were eviscerated and fixed for 48 h in 4% PFA at 4°C. Zebrafish were bleached in 30% H_2_0_2_/1% KOH for 2 h followed by 2 h in 15% H_2_0_2_/0.5% KOH. Zebrafish were cleared in 1% trypsin/2% borax solution overnight and stained in 1 mg/ml alizarin red in 1% KOH overnight. Following staining, fish were washed in increasing concentrations of glycerol in 1% KOH until 100% glycerol. Fish were imaged in 100% glycerol on a Zeiss Stemi SV11 microscope.

### Antibody staining

Embryos were fixed in 4% PFA in PBT overnight, then placed in methanol overnight at −20°C. They were permeabilized with acetone (Sigma) at −20°C for 20 min before transfer to PBT for 3× 5 min, then blocked in PBT plus 5% normal sheep serum (Sigma) for 1 h. Primary antibodies [Phospho-histone H3 (Ser10) from EMD Millipore Corporation 06570, or active Caspase 3 from BD Pharmingen 559565] were diluted 1/250 in blocking solution and applied overnight. Alexa-Fluor 555-Phalloidin (Thermo-Fisher, A34055) was added at 1/25 dilution. MF20 [Developmental Studies Hybridoma Bank (DSHB)] and Thbs4b (GeneTex GTX125689) were used at 1/250 according to the protocol described (Subramanian and Schilling 2014). After washing for 2 h in multiple changes of PBT, Goat anti-Rabbit-Alexa 488 or 647 secondary antibody (Thermo-Fisher, A11008 or A32733) was applied for 2 h in blocking solution. Embryos were washed with multiple changes of PBT overnight before being visualized using a Zeiss LSM700 confocal microscope. Cells positive for phospho-Histone H3 (PHH3) were counted blinded.

### RNA-sequencing

Zebrafish were genotyped from excised tail tissue, while matching trunk and head tissue from the same embryo was snap frozen at −80°C for RNA extraction after genotyping. Tails were exposed to 25 mM NaOH at 55°C for 30 min then neutralized with 40 mM Tris HCl pH5 to extract genomic DNA (Meeker *et al.* 2007) followed by PCR genotyping. Eight embryos of each genotype were pooled per replicate and total RNA was purified using RNeasy Plus Mini Kit (Qiagen). Three replicates each of wild-type sibling and *hnrnpul1/1l* mutants were sequenced using paired-end reads on Illumina NextSeq500 to a read depth of ∼100M reads. RNA libraries were prepared using NEBNext Ultra II Directional RNA Library Prep Kit (New England Biolabs). AS analysis of RNAseq data was completed using the Vertebrate AS and Transcript Tools (VAST-TOOLS) v2.2.2 (Irimia *et al.* 2014; Tapial *et al.* 2017). For all events, a minimum read coverage of 10 actual reads per sample was required, as described (Irimia *et al.* 2014) using genome release danRer10 (Torres-Méndez *et al.* 2019). Percent spliced in (PSI) values for single replicates were quantified for all types of alternative events, including single and complex exon skipping events (S, C1, C2, C3, ANN), microexons (MIC), alternative 5′ss and 3′ss (Alt5, Alt3), and retained introns (IR-S, IR-C). A minimum ΔPSI of 10% was required to define differentially spliced events upon each knockdown, as well as a minimum range of 5% between the PSI values of the 2 samples.

Differential gene expression analysis was performed using RPKM output from VAST-TOOL analysis. For each gene, *P*-values were determined by Student’s *t*-test of RPKM values from 3 biological replicates. Log_2_ FC was calculated using the mean RPKM for each genotype. A *P*-value of ≤0.05 was required to define a gene as significantly differentially expressed. Volcano plots and heat maps were performed using GraphPad Prism. Gene ontology and pathway analysis used Qiagen ingenuity pathway analysis (IPA).

### Statistical analysis

All experiments were performed in at least 3 independent biological replicates. All quantitative data are presented as mean ± standard deviation. Statistical analysis was performed using PRISM Graph Pad Software. ns, *P* > 0.05, *P ≤ 0.05, **P ≤ 0.01, ***P ≤ 0.001, ****P ≤ 0.0001.

## Results

### Generation of double homozygous zebrafish *hnrnpul1* and *hnrnpul1l* mutants

**Fig. 1.**
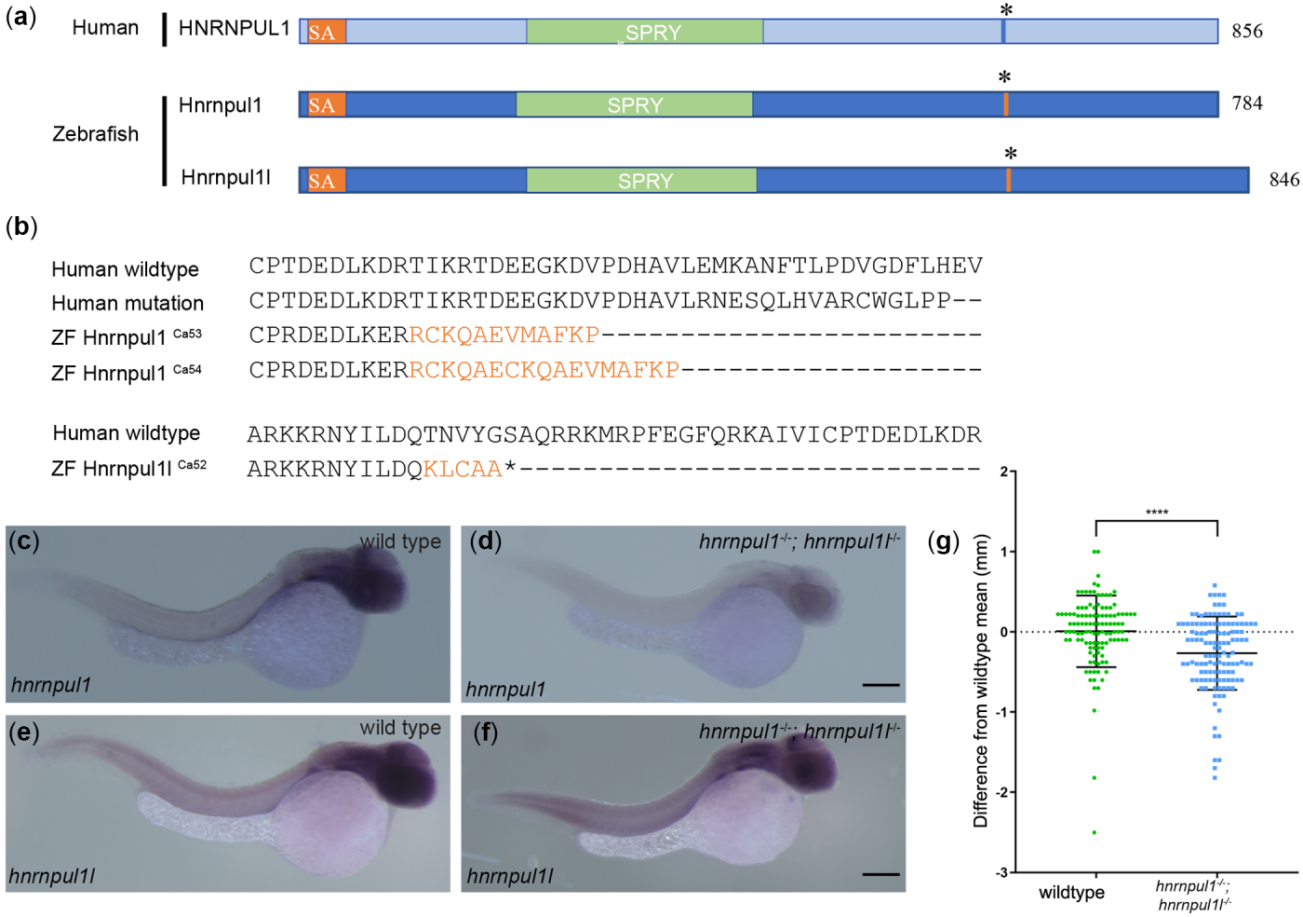
Mutation of human HNRNPUL1 and zebrafish *hnrnpul1* and *hnrnpul1l*. a) Schematic showing domains of human HNRNPUL1 and zebrafish Hnrnpul1 and Hnrnpul1l proteins. The mutation location in human HNRNPUL1 and equivalent sequence in zebrafish is marked by *. b) Comparisons of the locations of human HNRNPUL1 wild type, human mutant, and zebrafish *hnrnpul1*^ca53^ and ^ca54^ protein sequences showing frameshifted sequence (lighter font) following the mutation. Zebrafish *hnrnpul1l* wild type is compared to zebrafish *hnrnpul1l* in the exon 11 region where splicing to exon 13 creates a frameshift and stop codon after the new splice junction. c–f) WISH staining for *hnrnpul1* (c, d) and *hnrnpul1l* (e, f), at 24 hpf reveals expression that is not spatially restricted in wild types (c, e), but is reduced in maternal zygotic (MZ) *hnrnpul1/1l* mutants (d, f). g) Quantification of SL normalized to the mean wild-type SL of 16 dpf larvae. Wild type *n*= 115, *hnrnpul1/1l* MZ mutant *n*= 127. ****P < 0.0001, determined by Student’s *t*-test. Scale bars = 200 µm.

hnRNPUL1 has been studied in cell lines, however, there are no data on its role at the organismal level nor during animal development. We investigated its role in the zebrafish because of ease of genetic analysis. Due to the genome duplication in the teleost lineage, there are 2 closely related *HNRNPUL1* orthologs in zebrafish, *hnrnpul1* and *hnrnpul1l* and therefore we used a double knockout strategy (hereafter referred to as *hnrnpul1/1l* mutants, Fig. 1a). Human HNRNPUL1 protein sequence shows 65% similarity with zebrafish *hnrnpul1* (chromosome 18) and 67% with *hnrnpul1l* (chromosome 5), respectively. The DNA binding (SAP) and protein–protein interaction (SPRY) domains show higher conservation (76% and 77% similarity for Hnrnpul1 and 83% and 78% similarity for Hnrnpul1l, respectively). We identified 2 siblings with craniofacial and limb malformations with a homozygous frameshift variant of unknown significance (VUS) in *HNRNPUL1* [NM_007040.6: c.1805dup, p. (Glu560Argfs*17)]. The human variant is in exon 11, corresponding to exon 12 in both zebrafish *hnrnpul1* and *hnrnpul1l* (Supplementary Figs. 1 and 2). We designed CRISPR guides to target this site to introduce loss-of-function alleles into zebrafish. To ensure introduction of a loss-of-function mutation, a homology-directed repair “stop cassette” with stop codons in 3 reading frames was included in the CRISPR-Cas9 injections. *hnrnpul1*^Ca53^, *hnrnpul1*^Ca54^, and *hnrnpul1l*^Ca52^ alleles were isolated with mutations at similar locations to the human mutation in the DNA (Supplementary Fig. 1 and 2) and protein (Fig. 1b). *hnrnpul1*^Ca53^ has a 35-nucleotide insertion and *hnrnpul1*^Ca54^ has a 63-nucleotide insertion. Both *hnrnpul1*^Ca53^ and *hnrnpul1*^Ca54^ mutations create a frame shift and premature stop codon resulting in truncation of 6 amino acids after the mutation for allele Ca53, and 11 amino acids for Ca54. *hnrnpul1l*^Ca52^ has a 106-nucleotide insertion, resulting in a frameshift mutation and premature stop codon. This is predicted to produce a nonsense protein mutation resulting in truncation of 3 amino acids after the mutation (Fig. 1b). All 3 alleles are predicted to result in a truncated protein. No phenotypic differences between the *hnrnpul1* Ca53 and Ca54 alleles were noted—single and double mutants were viable and fertile. Unless otherwise noted, all data in the manuscript were collected from maternal zygotic double mutants for *hnrnpul1*^ca54−/−^; *hnrnpul1*l^*c*a52−/−^.

We determined the expression pattern of both *hnrnpul1* and *hnrnpul1l* in embryos at 24 hpf. Both transcripts were broadly expressed (i.e. ubiquitously; Fig. 1, c and e). These results are in line with previous reports in the Zebrafish Information Network that expression is “not spatially restricted” for *hnrnpul1* from 1 cell to pec-fin stage (60 hpf; Thisse and Thisse 2004). No previous expression analysis is available for *hnrnpul1l*. *hnrnpul1* mutation results in strongly decreased *hnrnpul1* expression in homozygous mutants, suggesting that nonsense-mediated decay is activated, while *hnrnpul1l* is expressed relatively normally in double mutants (Fig. 1, d and f). Analysis of the gross morphology of *hnrnpul1/1l* double homozygous mutants shows *hnrnpul1/1l* mutant larvae are significantly smaller than wild-type embryos (Fig. 1g). Additionally, low-frequency developmental abnormalities including edema and embryo curvature (Supplementary Fig. 3) are seen; however, viable and fertile adults were obtained for all allelic combinations including *hnrnpul1/1l* double mutants.

### *hnrnpul1/1l* modulates AS and transcription

**Fig. 2.**
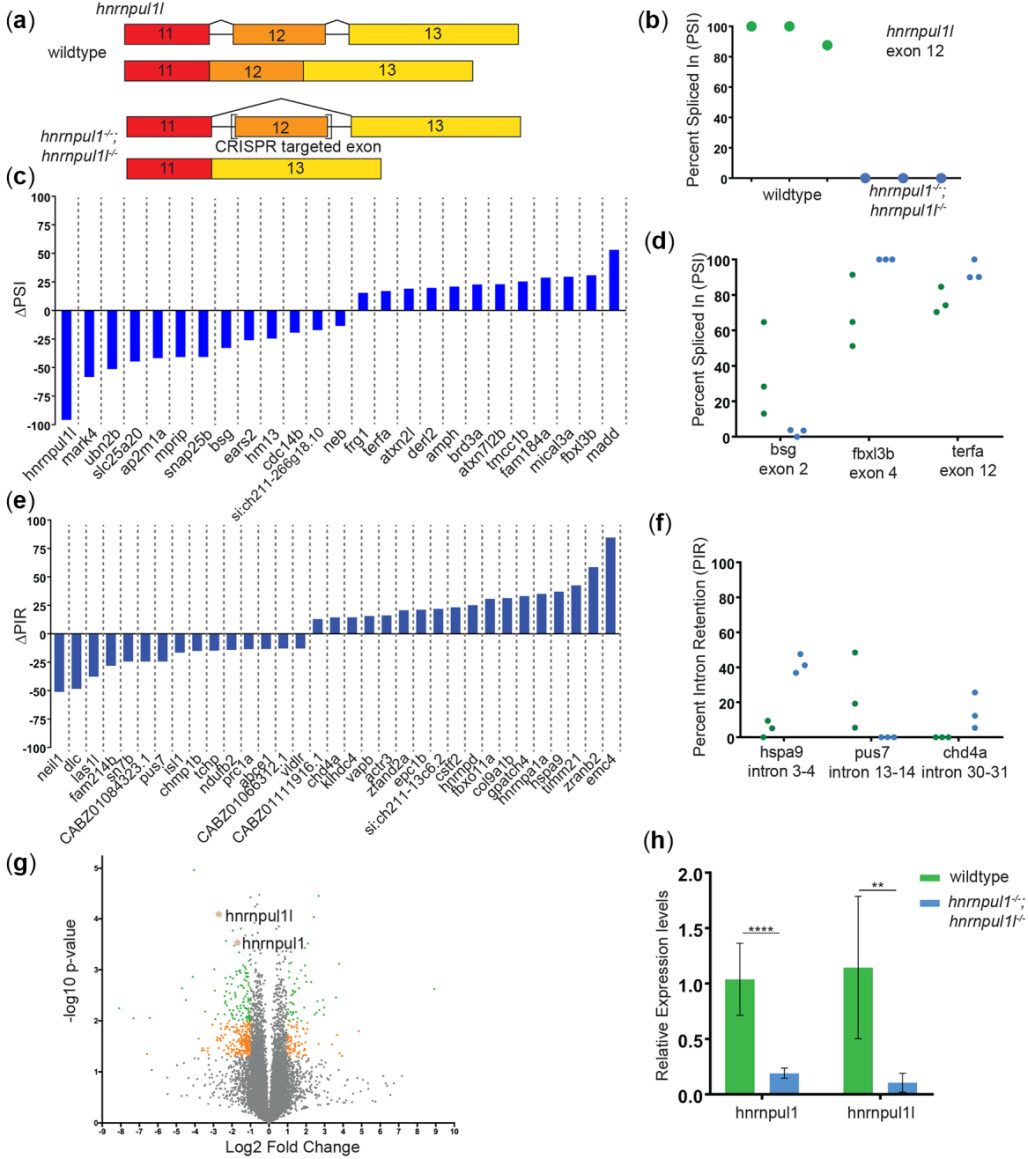
Loss of *hnrnpul1l* and *hnrnpul1* leads to differential splicing. a) Schematic showing the processing of hnrnpul1l to form standard transcript and AS of exon 12 of the *hnrnpul1l* gene as a result of CRISPR-Cas9 targeted mutagenesis. b) PSI for exon 12 of *hnrnpul1l* in wild type and *hnrnpul1/1l* mutant embryos at 3 dpf. c) Change in PSI of all exon skipping events in *hnrnpul1/1l* mutant embryos compared to wild types. d) Detailed view of PSI of genes associated with phenotypes, points represent each biological replicate. e) Change in percent intron retention (PIR) of all intron retention events in *hnrnpul1/1l* mutant embryos compared to wild types. f) Detailed view of PIR of genes associated with phenotypes, points represent each biological replicate. Details of affected exon/intron are given in Supplementary Table 1. g) Volcano plot showing all differentially expressed genes. Gray points = 1>Log2 FC>-1 or *P* > 0.05, orange points = 1<Log2 FC<-1 and *P* ≤ 0.05, green points = 1<Log2 FC<-1 and *P* ≤ 0.01. h) qPCR validation *hnrnpul1* and *hnrnpul1l* expression in *hnrnpul1/1l* mutants at 3 dpf compared to wild types. **P ≤ 0.01, ****P ≤ 0.0001, determined by Student’s *t*-test.

The hnRNP family regulates AS, but knowledge of the specificity and targets of *hnrnpul1* during development is limited. Thus, we performed paired-end bulk RNAseq to identify differentially spliced events between embryos genotyped as wild type (from an incross of *hnrnpul1l* heterozygotes), and embryos genotyped as double mutant (MZ for *hnrnpul1l* and zygotic mutant for *hnrnpul1*) at 3 dpf, a stage where fins and tendons are developing. We used VAST-TOOLS to identify splice junctions and characterize splicing events (Irimia *et al.* 2014; Tapial *et al.* 2017). We observed AS events in 76 different mRNAs: 25 skipped exons, 33 retained introns, and 7 alternative 3′ splice site and 11 alternative 5′ splice site changes in mutants (Fig. 2, Supplementary Table 1). The most differentially expressed exon in our AS analysis was exon 12 of *hnrnpul1l*, the exon containing the mutation (Fig. 2, a and b). This exon is skipped 100% of the time in genetic mutants as determined by gel analysis and VAST-TOOLS (Supplementary Fig. 2). In contrast, exon skipping is not seen in the *hnrnpul1* gene. PCR or RNAseq displays a variety of splice forms, most of which are not canonically spliced as exon–exon junctions but result in intron retention and unpredicted variants (Supplementary Fig. 1). As we have noted there is nonsense-mediated decay of *hnrnpul1*; any transcript detectable by these techniques represents a very low expression level compared to wild type.

We identified splicing changes in many mRNAs from genes with a variety of developmental roles (Fig. 2, c and e). *basigin (bsg/*CD147) showed a 33% reduction in exon 2 usage in *hnrnpul1/1l* mutants compared to wild type. *F-box and leucine-rich repeat protein 3 (fbxl3b)* showed a 31% increase in exon 4 usage in *hnrnpul1/1l* mutants compared to wild type, while *telomeric repeat binding factor a (terfa)* showed a 17% increase in exon 12 usage (Fig. 2d). We also observed changes in intron retention (Fig. 2e). *Heat shock protein a9 (hspa9)* showed a 37% increase in retention of intron 3-4, while *chromodomain helicase DNA-binding protein 4a (chd4a)* showed a 14% increase in retention of intron 30–31 in *hnrnpul1/1l* mutants compared to wild type. *Pseudouridylate synthase 7 (pus7)* showed a 24% reduction in the retention of intron 13–14 in *hnrnpul1/1l* mutants (Fig. 2f).

Differential gene expression analysis identified 1,575 genes that were significantly changed (*P* < 0.05) with 1,003 genes downregulated (660 genes downregulated >1.5-fold), and 572 genes upregulated (333 genes upregulated >1.5-fold; Fig. 2g;Supplementary Table 2). *hnrnpull1l* were downregulated 6.5-fold and *hnrnpul1* is downregulated 3-fold in this analysis, consistent with our observations of nonsense-mediated decay (Fig. 2h). To identify pathways that are dysregulated in *hnrnpul1/1l* mutants, we used IPA. There was significant upregulation of several pathways including translation (EIF4 and EIF2 signaling), protein ubiquitination, DNA damage via the 14-3-3 pathway, and the kinetochore metaphase pathways (Supplementary Fig. 4 and Supplementary Table 3). With respect to translation and ribosomal pathway changes, *eif1ax*, *eif33b*, and *eif3k* factors were upregulated along with 14 *rpl* genes for the large ribosomal subunit, 6 *rps* genes for the small ribosomal subunit, and the 40S ribosomal protein S30, *fau* (Supplementary Fig. 4 and Supplementary Table 3). In the kinetochore-metaphase pathway, *cyclin B1*, cyclin-dependent kinase *cdk1*, and the kinetochore protein *zwilch* were upregulated among other genes (Supplementary Fig. 4 and Supplementary Table 3)*.* Although most pathways were upregulated (consistent with human HNRNPUL1 being a transcriptional repressor), we also find that early growth response genes *egr1* and *egr4* were downregulated 1.7- and 2.9-fold, respectively, suggesting that cell division and differentiation might be reduced in mutants.

Thus, loss of *hnrnpul1/1l* leads to changes in splicing and transcriptional regulation of genes involved in fundamental cellular processes (translation, ubiquitination, and cell cycle), as well as developmental genes.

### *hnrnpul1* mutation results in reduced fin growth but not fin specification

**Fig. 3.**
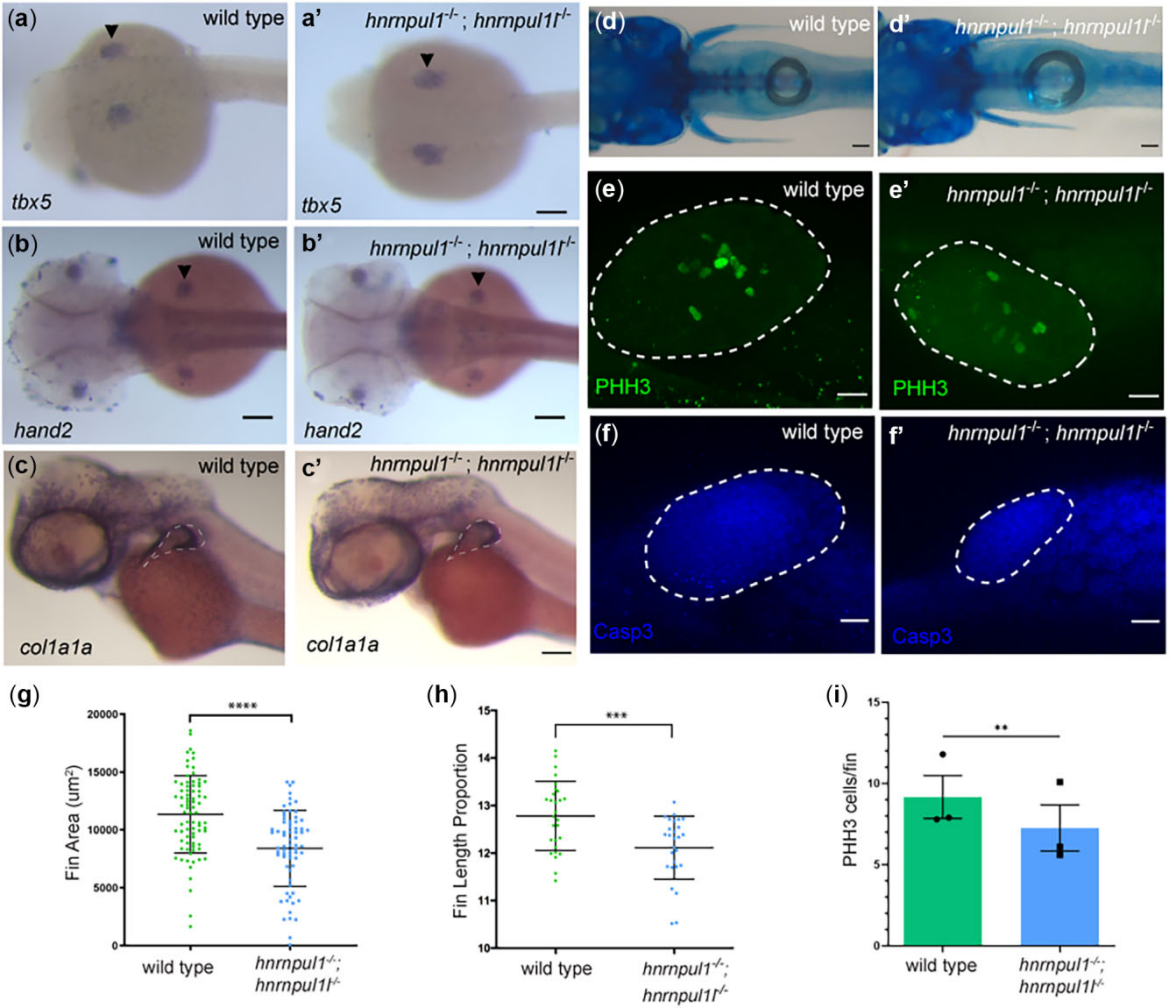
Loss of *hnrnpul1* and *hnrnpul1l* does not affect fin specification, but leads to decreased fin growth in embryos and larvae. a–c) mRNA expression of fin specification markers *tbx5* (a, a’) at 24 hpf, and *hand2* (b, b’) at 48 hpf in wild type (a, b) and *hnrnpul1/1l* mutant (a’, b’) embryos. c, c’) mRNA expression of *col1a1a* in wild type and *hnrnpul1/1l* mutant embryos at 48 hpf. Dashed line shows outside edge of staining where fin size was measured. d, d’) Alcian blue cartilage staining of wild type and *hnrnpul1/1l* mutant fish at 16 dpf. e, e’) PHH3 staining of wild type and *hnrnpul1/1l* mutant fins at 48 hpf. f, f’) Activated Caspase 3 (Casp3) immunostaining in wild type and *hnrnpul1/1l* mutant fins at 48 hpf. Dotted lines show the fin boundary. g) Quantification of fin area in wild type and *hnrnpul1/1l* mutant col1a1a stained embryos at 48 hpf. Wild type *n* = 79, *hnrnpul1/1l* *n*= 68, from 2 trials. h) Quantification of fin length at 16 dpf as a percentage of body length. Wild type *n* = 28, *hnrnpul1/1l* *n* = 27, from 2 trials. i) Quantification of proliferation via PHH3 immunostaining in wild type and *hnrnpul1/1l* mutant fins at 48 hpf, *n* = 30 fins, from 3 trials. **P ≤ 0.01, ***P ≤ 0.001, ****P ≤ 0.0001, ns = *P* > 0.05, determined using Student’s *t*-test. Scale bars: a–d’ = 100 µm, e–f’ = 20 µm.

Pathogenic variants in the minor spliceosome have been linked to primordial dwarfism and limb-growth defects (Edery *et al.* 2011; Drake *et al.* 2020). The paired pectoral fins of mutant zebrafish are a paralogous structure to mammalian limbs. To test whether fin/limb specification is affected by loss of *hnrnpul1/1l*, we examined the expression of *hand2*, *tbx5*, and *gli3* by in situ hybridization. We found their expression was normal in *hnrnpul1/1l* mutants (Fig. 3, a–b’, Supplementary Fig. 5). We also tested the expression of *wnt5b*, a wingless/wnt gene highly expressed in fin, and found it was also unchanged in mutants (Supplementary Fig. 5). Thus, fins appeared to be normally specified in *hnrnpul1/1l* mutants.

We did, however, find changes in fin growth. *col1a1a* localizes to the apical fold (AF) of the developing fin bud at 48 hpf (Fig. 3, c and c’). Using the outer edge of *col1a1a* staining to mark the fin boundary, we measured fin area. *hnrnpul1/1l* mutants had significantly smaller fin bud area (8,415 ± 3,282 um^2^) compared to wild types (11,350 ± 3,338 um^2^, *P* < 0.0001; Fig. 3g). We next tested fin size at larval stages using Alcian blue staining at 16 dpf (Fig. 3, d and d’). As larval fish differ in growth rates, to ensure smaller fin length was not due to overall smaller fish size we calculated the fin length as a proportion of standard length (SL; tip to tail) for each animal. We find that *hnrnpul1/1l* mutants have significantly shorter fins compared to wild type with 12.8 ± 0.7% of body length in wild-type animals and 12.1 ± 0.7% of body length in *hnrnpul1/1l* mutant animals (*P* = 0.0008; Fig. 3h). To ensure that decreased fin size was not due to defective overall growth, we quantified eye size but find no significant difference between *hnrnpul1/1l* mutants and wild types, suggesting that the growth defect at this stage is specific to the fin and not the entire body (Supplementary Fig. 5). We also tested whether the endochondral disc or fin fold was specifically affected. We measured both the disc size compared it to the total fin length (disc and fold) in 16 dpf *hnrnpul1/1l* mutants and wild types. There is no significant difference in the ratio of disc: fin ratio indicating that both disc and fin fold are proportionately affected. Our data suggest that fin size is reduced from embryonic through larval stages.

We tested the mechanism for reduced fin size by quantitating cell death and proliferation of fin mesenchymal cells. Staining for activated Caspase 3 protein revealed no differences between wild-type and mutant staining patterns (Fig. 3, f and f’). However, when we stained for a proliferation marker, PHH3, that marks the nuclei of cells in mitosis, we detected a significant decrease from an average of 9 positive nuclei per fin at 48 hpf in wild type, to an average of 7 positive nuclei per fin in *hnrnpul1/1l* mutant (*P* = 0.006, Fig. 3, e, e’, and i).

Taken together, our data suggest that *hnrnpul1* and *hnrnpul1l* play a role in regulating fin growth in embryos and larvae, but not in initial fin bud cell specification. This suggests that deficient proliferation in embryonic fins may lead to decreased fin size.

### Skeletal defects in *hnrnpul1* mutant fins

**Fig. 4.**
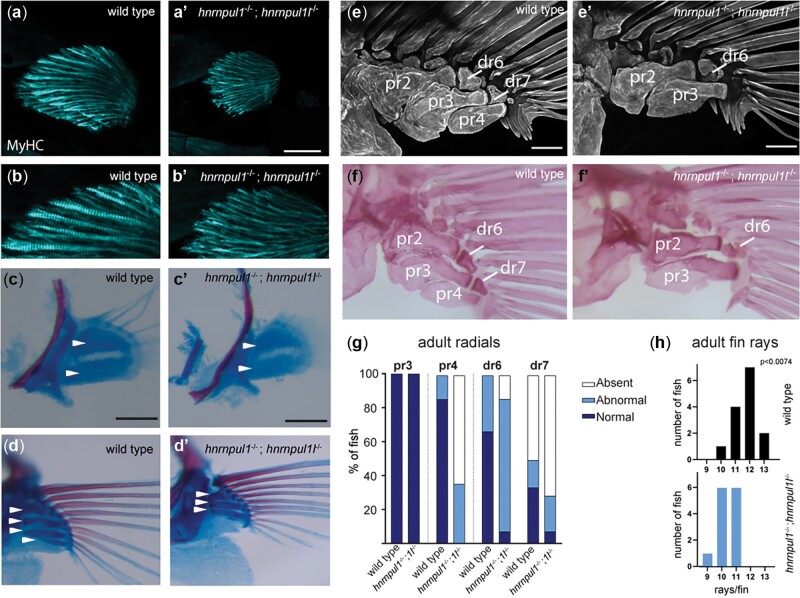
Musculoskeletal defects in *hnrnpul1/1l* mutant fins. a, b) Fin muscle, as highlighted by MF20 staining for myosin heavy chain (MyHC) in wild type (a, b) and *hnrnpul1/1l* mutant (a’, b’) fins at 72 hpf. Scale bar = 50 μm. c–f) Alcian blue (cartilage) and alarizin red (bone) staining. c, c’) At early stages (40 dpf, SL = 6.2–6.7 mm) 2 CSZs are present in both wild type and *hnrnpul1/1l* mutant fins (arrowheads, scale bars = 50 μm). d, d’) At larval stages (40 dpf, SL = 11 mm) there are 4 proximal radial precursors in wild-type fins, but 3 proximal radial precursors in *hnrnpul1/1l* mutant fins (arrowheads; scale bars = 100 μm). e, f’) In adult fish (3-month old), alizarin red staining fluorescent imaged (e, e’) or brightfield imaged (f, f’) shows a missing proximal radial (pr4) and distal radial 7 (dr7) in a *hnrnpul1/1l* mutant. Scale bars are 200 µm. g) Quantification of the frequency of missing proximal and distal radials in adults (*n* = 12 wild type, 14 *hnrnpul1/1l* mutants). h) Quantification of the frequency of fins with the indicated number of rays per fin (*n* = 14 wild type, 13 *hnrnpul1/1l* mutant, *P* < 0.074, chi-squared test).

We found that not only are *hnrnpul1/1l* mutant fins smaller, but also they showed skeletal patterning defects. In early development, the endoskeletal disc is located between the adductor and abductor muscles of the fin. Myosin II heavy chain (monoclonal antibody MF20 staining) at 72 hpf shows that muscle was present in both mutant and wild-type fins (Fig. 4, a–b’), however, muscle fiber size is slightly smaller on average in *hnrnpul1/1l* mutant fins (Supplementary Fig. 6).

We examined cartilage and bone development using Alcian blue and Alarizin red staining, respectively. As zebrafish larval growth is highly variable depending on food availability and spacing, all fish were categorized by SL. Wild types and mutants between 6.2 and 6.7 mm SL showed 2 cartilage subdivision zones (CSZ’s) as expected in developing fins (Fig. 4, c and c’). As larvae continue growing, the CSZ’s divide into cartilage elements that will become bones. In larger 1.1 mm SL siblings, we observed a striking loss of a fourth cartilage rudiment (white arrows) in mutant fins (Fig. 4, d and d’).

At 16 weeks post fertilization, zebrafish are young adults. We found that bones on the posterior side of the fin are affected in mutants. While the third proximal radial, pr3, was present in all mutants and wild types, there was a striking loss of the proximal radial 4 (pr4). One hundred percent of *hnrnpul1/1l* mutants have abnormal or missing pr4 vs wild types that show 14% abnormal pr4, with none missing. Similarly, distal radial 7 (dr7), which is variably present in wild types, it is more frequently absent in *hnrnpul1/1l* mutants (Fig. 4, e–g). There is a similar loss of fin rays adjacent to pr4/dr7 in *hnrnpul1/1l* mutants with an average of 11.7 rays per fin in wild types, and 10.3 rays per fin in *hnrnpul1/1l* mutants (*P* = 0.0074; Chi-square; Fig. 4h). Thus, *hnrnpul1/1l* mutants have a loss of pectoral-fin skeletal elements including proximal and distal radials, and fin rays.

### *hnrnpul1* mutation results in increased incidence of caudal scoliosis in adult zebrafish

**Fig. 5.**
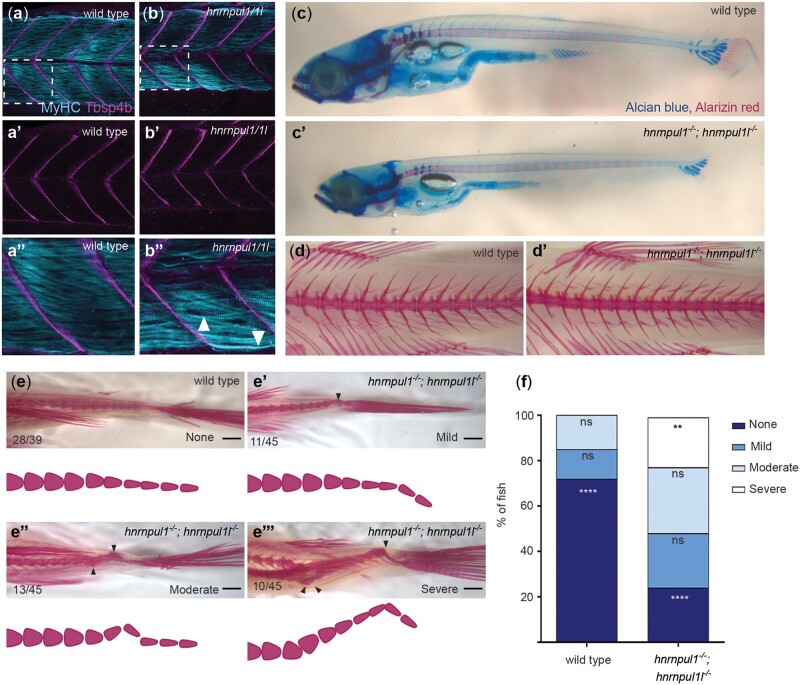
Loss of hnrnpul1l and hnrnpul1 leads to caudal scoliosis in adult Zebrafish. a, b) Muscle (MyHC, blue) and tendon (Tbsp4b, magenta) staining of 72 hpf wild type (a, a’’) and *hnrnpul1/1l* mutant (b, b’’) embryos, arrowheads highlight less organized muscle fibers in *hnrnpul1/1l* mutants. c, c’) Alcian blue cartilage and alarizin red bone staining of 16 dpf wild type and *hnrnpul1/1l*. d, d’) Alizarin red bone staining of 16-week-old adult wild type (d) and *hnrnpul1/1l* mutant (d’) fish. e, e’’’) Alizarin red bone staining of 16-week-old adult wild type and *hnrnpul1/1l* mutants graded by scoliosis in the caudal (tail) region (arrowheads) as none (e), mild (e’), moderate (e’’), and severe (e’’’). Schematics show spine curvature from the images above. f) Quantification of the proportion of fish with none, mild, moderate, or severe scoliosis. Wild type *n* = 39, *hnrnpul1/1l* mutant *n* = 45, from 3 trials. **P ≤ 0.01, ****P ≤ 0.0001 determined by Fisher’s exact test. Scale bar = 1000 µm (e, e’’’).

Sixteen-week post fertilization, young adult *hnrnpul1/1l* mutants showed scoliosis in the caudal spine adjacent to the tail. We tested whether this developed early in development by first looking at muscle and tendon development. We stained 72 hpf wild types and *hnrnpul1* mutants for myosin heavy chain (MF20; muscle) and Thrombospondin 4b (Tbsp4b; tendon). The myotendinous junctions between somites of the trunk showed no differences between mutants and wild types (Fig. 5, a–b’). Muscle fiber size was indistinguishable from wild type (Supplementary Fig. 6).

To understand the origins of scoliosis, we tested different larval stages. We carefully controlled housing density, a factor that influences growth rates. Fish were housed at a density of 10 per 3 L tank from 4.5 weeks until 16 weeks of age, at which point they were stained with Alcian blue and alizarin red to visualize cartilage and bone (respectively). Although *hnrnpul1/1l* mutant larvae were smaller than wild-type larvae at 16 dpf, the development of cartilage and bone in the spinal column was comparable among mutants and wild-type fish at this stage (Fig. 5, c and c’). At 16 weeks post fertilization, vertebrae of the mid-trunk were also normally formed in both wild types and mutants (Fig. 5, d and d’). However, when we looked at the very caudal region of the animal, we observed penetrant scoliosis in mutants. The relative severity of spinal curvature was scored as none, mild, moderate, or severe scoliosis (Fig. 5, e–e’’’). The incidence of scoliosis was significantly higher in *hnrnpul1/1l* mutants (76%) compared to wild types (28%, *P* < 0.0001; Fig. 5f). The most severe phenotype occurred only in *hnrnpul1/1l* mutants. Overall, our data suggest that loss of *hnrnpul1*/*1l* contributed to caudal scoliosis in young adults.

### *hnrnpul1* mutation results in craniofacial defects

**Fig. 6.**
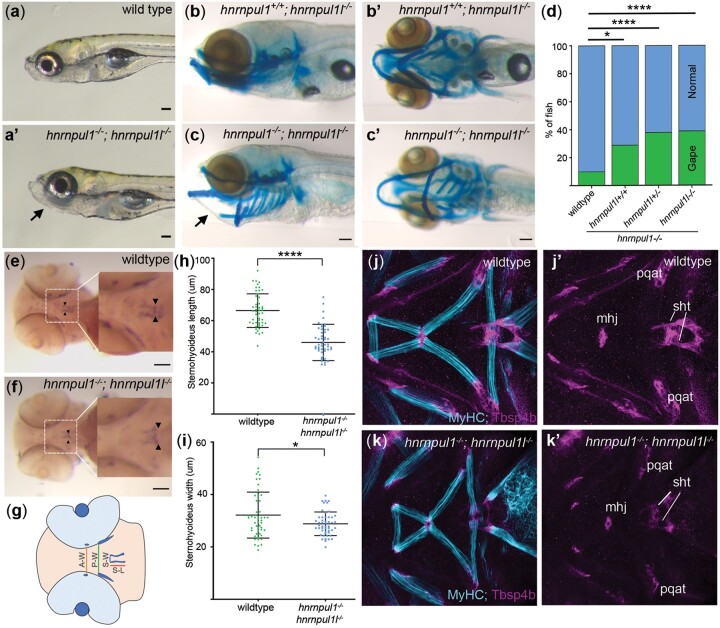
*hnrnpul1/1l* mutants show a shortened sternohyoideus tendon. a, a’) Images of live 8 dpf wild type and *hnrnpul1/1l* mutant larvae. b–c’) Lateral (b, c), and ventral (b’, c’) Alcian blue staining at 8 dpf in normal *hnrnpul1l*^−/−^ mutant (b, b’) and a gaping-jaw phenotype in *hnrnpul1/1l* mutant (arrows) (c, c’). d) Quantification of the proportion of fish showing a gaping-jaw phenotype. Wild type *n* = 283, *hnrnpul1l^−/−^; hnrnpul^+/+^* *n* = 24, *hnrnpul1l^−/−^; hnrnpul1^+/−^* *n* = 64, *hnrnpul1^−/−^hnrnpul1l^−/−^ n* = 84 from 5 trials. e, f) WISH staining for scleraxis (scxa) in the sternohyoideus tendon (box, arrow heads) in wild type and *hnrnpul1/1l* mutant embryos at 72 hpf. g) Schematic showing craniofacial tendons and location of tendon measurements. A-W = width between adductor mandibulae tendons, P-W = width between Palatoquadrate tendons, S-W= width between sternohyoideus tendons, S-L = sternohyoideus length. h, i) Quantification of the length (h) and width (i) between the sternohyoideus tendons in wild type (*n* = 51) and *hnrnpul1/1l* mutant (*n* = 50) embryos, from 3 trials. j–k’) Muscle (MyHC, blue) and tendon (Thbs4b, magenta) staining of the craniofacial region of 72 hpf wild type (j, j’) and *hnrnpul1/1l* mutant (k, k’). *P ≤ 0.05, ****P ≤ 0.0001 determined by Fisher’s exact test (d) and Student’s *t*-test (h, j) Scale bars = 100 µm. mhj, mandibulohyoid junction; pqat, palatoquadrate adductor tendon; sht, sternohyoideus tendon.

One of the most obvious phenotypes visible in mutant larvae was a jaw that gapes open at 8 dpf as compared wild types (Fig. 6, a and a’). The incidence of an open jaw is significantly increased in an allelic series (wild type = 10%, *hnrnpul1^+/+^; hnrnpul1l^−/−^* = 29%, *hnrnpul1^+/−^; hnrnpul1l^−/−^* = 38%, *hnrnpul1^−/−^hnrnpul1l^−/−^*= 39%; Fig. 6d). Alcian blue staining shows cartilage was correctly patterned in mutants as compared to wild types, however, and therefore we examined whether the jaw opening could be due to defects in muscle and tendon formation (Fig. 6, b–c’).

We analyzed the development of tendons in the embryonic craniofacial region that are responsible for opening and closing of the jaw (Fig. 6g). Expression of *scleraxis* (*scxa*), a tendon-specific marker at 72 hpf showed that *hnrnpul1/1l* mutants had a significantly shorter sternohyoideus tendon (wild type = 0.66 ± 0.1 µm, *hnrnpul1/1l *=* *0.47 ± 0.1 µm, *P* < 0.0001; Fig. 6, e, f, and h). The distance between the most anterior points of the sternohyoideus tendons was also significantly narrower in *hnrnpul1/1l* mutants compared to wild types (wild type = 0.35 ± 0.09 µm, *hnrnpul1/1l *=* *0.30 ± 0.05 µm, *P* = 0.007; Fig. 6, e, f, and i). However, we found no difference in the spacing of other craniofacial tendons, the adductor mandibulae or palatoquadrate tendons (Supplementary Fig. 7). These latter tendons are not responsible for jaw movement. Staining for muscle (MyHC) and myotendinous junctions (Thsp4b) in wild types and mutants revealed that craniofacial tendons and muscles were present and correctly patterned. We found a significant decrease in muscle fiber width in this region, potentially indicating weaker muscles (Supplementary Fig. 6). Craniofacial tendons develop from neural crest cell (NCC) populations; therefore, we analyzed the expression of the NCC markers *foxd3* and *sox10.* Expression of these specification markers at 12 and 32 hpf was normal in *hnrnpul1/1l* mutants (Supplementary Fig. 8). Thus, our data suggest the sternohyoideus tendon does not grow to a normal size. Interplay between weak muscle and shorter tendons may contribute to the gaping jaw phenotype by not allowing the mandible to close properly.

### *HNRNPUL1* as a candidate gene for human developmental anomalies

We found a VUS in the novel candidate disease gene *HNRNPUL1* using whole-exome sequencing of 2 similarly affected sisters with craniofacial and limb abnormalities. These sisters were born to consanguineous first cousin parents of Arab descent. Given the similarity between the 2 sisters but no family history of craniofacial or limb abnormalities, this suggested a potentially biallelic autosomal recessive inheritance pattern. Our analysis identified the variant c.1673dup, p. (Glu560Argfs*17) in the gene *HNRNPUL1.* This gene has not been previously associated with a known genetic condition. We did not detect any additional patients with variants in this gene using web-based tools such as Matchmaker exchange (Buske *et al.* 2015) or GeneMatcher (Sobreira *et al.* 2015).

**Fig. 7.**
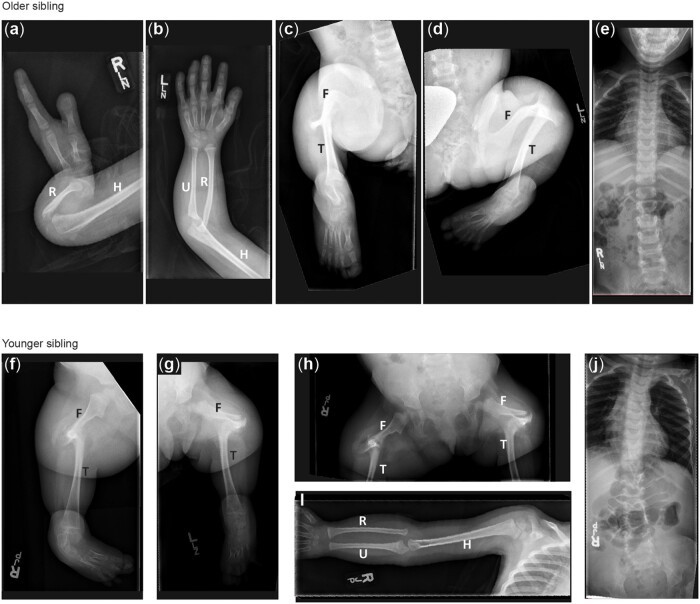
Radiographic features of siblings with a VUS in *HNRNPUL1*. a–e) X-ray images of older sibling. Right arm showing short humerus and absent ulna with 2 fixed in extension digits of the right hand (a). Left arm showing short humerus and normal upper arm (b). Right (c) and Left (d) legs showing mid-shaft femoral pseudoarthroses, fused tibia to the femoral condyles, absent fibulas, and abnormal toes. f–j) X-ray images of the younger sibling Right (f) and Left (g) legs showing bilateral fibular agenesis, short and bowed femurs, and 4 metarsals and tarsals (h). Right arm (i) showing normal upper limb development.

The probands both presented at birth with multiple skeletal malformations following uncomplicated pregnancies. The older sister was born to a 27-year-old G4P4 (gravidity and parity) woman. Limb malformations were noted on a 27-week ultrasound. The child was born at 38-weeks of gestation by spontaneous vaginal delivery with a birth weight of 3.8 kg (75–90th percentile). Outside of limb malformations and dysmorphic features, the child’s neonatal examination and clinical course were unremarkable. She was born with bilateral short humeri, right absent ulna, with 2 fixed in extension digits of the right hand; 5 rays present but with missing carpals, abnormal nails, and dorsal creases on the left hand (Fig. 7, a and b). Her lower limbs had mid-shaft femoral pseudoarthroses, fused tibia to the femoral condyles, absent fibulas, and abnormal toes (Fig. 7, c and d). Her karyotype shows mosaic Turner syndrome, which is thought to be an incidental finding. Her course has been mostly uncomplicated, except for orthopedic issues, however, she does have a significant learning disability. She is minimally dysmorphic with hypertelorism, upslanting palpebral fissures, prominent eyes and eyelashes, and a high palate.

The younger sister was born at term to a 28-year-old G5P5 woman. Her birth weight was 4.03 kg (75–90th percentile) with a head circumference of 39.0 cm (+2.5 SD). She had bilateral-absent fibular, short, and bowed femurs, and 4 metatarsals and tarsals bilaterally (Fig. 7, f, g, and h). Upper limb development was normal (Fig. 7i) with the exception of the hands showing stiff index finger dorsal creases and abnormal nails progressively more severe from ray 5 to 1, despite the presence of 5 rays. Her echocardiogram and abdominal ultrasounds were normal. She was felt to be minimally dysmorphic with a prominent forehead, relative macrocephaly, bitemporal narrowing, hypertelorism with prominent eyes, and heavy eyelashes. Her palate was high but there was no clefting. Her clinical course has been mostly unremarkable outside of orthopedic complications and her intelligence is felt to be within the normal range. In addition to their skeletal malformations, both sisters had bicornuate uterus, hirsutism, and the first sister showed bilateral hydronephrosis, with the second sister showing Dandy-Walker malformation. Clinically, the sisters share some features with Al-Awadi/Raas-Rothschild/Furhmann syndrome (OMIM 276820), which arises due to homozygous variants in *WNT7A*, however, *WNT7A* sequencing was negative.

Whole-exome sequence analysis was undertaken as part of the Care for Rare Canada consortium in both affected siblings. Variants were filtered based on rarity in the internal variant database and the Genome Aggregation Database (gnomAD), along with predicted effects on protein function (Beaulieu *et al.* 2014). Given the family structure, an autosomal recessive mode of inheritance was favored, although heterozygous variants present in both siblings were assessed since germline mosaicism could not be excluded. Rare homozygous variants were identified in both affected siblings in the *PODXL*, *CFTR*, *HNRNPUL1*, and *PRKD2* genes. The variants in PODXL, CFTR, and PRKD2 were excluded as causative candidates (Supplementary Table 3).

The rare homozygous variant in *HNRNPUL1* [Chr19 (GRCh37):g.41807595dup, NM_007040.5 (*HNRNPUL1*):c.1673dup, p. (Glu560Argfs*17)] is predicted to result in a frameshift and premature stop approximately two-thirds of the way through the protein. No individuals with homozygous loss-of-function variants have been previously reported, and data from the gnomAD suggest that *HNRNPUL1* loss-of-function variants are not tolerated in humans. Segregation of this variant by Sanger sequence analysis of both affected individuals, both parents, and 4 unaffected siblings shows that both patients are homozygous, both parents are heterozygous for the c.1673dup variant, while unaffected siblings are not homozygous for this variant.

## Discussion

Although we can detect changes in AS using sequencing technology, we still have an incomplete understanding of how global mutations in splicing factors impact tissue-specific development and disease (Cieply and Carstens 2015; Suzuki *et al.* 2019). Phenotypes resulting from loss of *hnrnpul1/1l* in zebrafish are observed in multiple systems from fin growth and patterning to skeletal morphology and tendon growth. This is not surprising, as *hnrnpul1* and *hnrnpul1l* have a widespread expression pattern in embryonic zebrafish. It is particularly interesting that the phenotypes we observe are developmental. We are the first to show that *hnrnpul1/1l* affects both AS and transcriptional regulation of mRNAs in vivo. Tissue-specific phenotypes likely occur through varying composition of components of the spliceosome within different tissues [reviewed in Baralle and Giudice (2017)] and target transcript expression in tissues.

We cannot verify that the human variant in the candidate gene *HNRNPUL1* is pathogenic without identifying additional affected individuals with comparable phenotypes, and/or further studies with the patient mutations. However, the viability and phenotypes of both humans and zebrafish mutants provide evidence that mutations in this gene are tolerated in animals but result in severe developmental consequences. Loss of *HNRNPUL1* may be tolerated because even a complete loss of a splicing regulator may not lead to complete loss or mis-splicing of targets. For instance, we show that there is a difference in the ratio, but not a complete loss of splice variants in zebrafish *hnrnpul1* mutants. A number of key pathways and genes regulated at splicing or transcription levels by Hnrnpul1 influence cell and organismal growth.

### Involvement of Hnrnpul1 in fin and limb growth

Paired fins of teleosts (including zebrafish) are ancestral to limbs in tetrapods (including humans) as they are both derived from locomotive organs in common ancestral vertebrates (Shubin *et al.* 1997). Although patterning and development of the skeleton are not identical across species, the same genetic pathways are used to set up fin and limb development. Specification of limb and fin-bud tissue is marked by expression of *tbx5* as early as 14–16 hpf in the zebrafish (Gibert *et al.* 2006). Following specification, at approximately 23–26 hpf, condensation of mesenchymal cells forms the fin bud. Similar to mammals, the fish fin develops an apical ectodermal ridge (AER) as a signaling center driving mesenchymal cell proliferation and growth. In fish, the AER transforms into the AF at approximately 36 hpf. The limb/fin bud actively grows as mesenchymal cells organize and begin to differentiate muscle around 46–48 hpf (Grandel and Schulte-Merker 1998). The roles of genes in limb specification, such as retinoic acid, *tbx5*, *fgf10*, *hand2*, and *wnt5* are conserved across vertebrates (Mercader 2007). We found no changes in the expression of specification genes in zebrafish *hnrnupl1/1l* mutants, suggesting that the fin is specified correctly.

However, relatively quickly after specification, we see differences in fin growth. Limb mesenchymal proliferation is driven by FGF signals from the AER in fish and tetrapods. Fgf10 induces Fgf8 via Wnt3 in chick, mouse, and zebrafish (Mercader 2007; Yano and Tamura 2013). We observed a decrease in cell proliferation in *hnrnpul1/1l* mutants, but no change in cell death. This observation suggests that a reduction in normal proliferation and cell cycling prevents normal proliferation of fin mesenchyme in embryogenesis. The deficiency in fin size is also seen at larval stages, where fins are disproportionately smaller in mutants.

Patterning of the pectoral-fin endoskeleton occurs during mid-larval stages, arising from cells of the embryonic fin-bud mesenchyme that develop into the endoskeletal disc. During larval stages, the disc forms 2 CSZs separated by a zone of matrix decomposition. These zones then divide to form 4 cartilage rudiments that will form the 4 long bones, the proximal radials. Six to 8 smaller distal radials also form (Grandel and Schulte-Merker 1998). The ∼12 fin rays located on the distal portion of the fin are derived from the fin fold. *hnrnpul1/1l* mutants show striking differences from wild-type patterning. The initial formation of 2 CSZs occurs normally, but in larger larvae, wild types show 4 cartilage ruminants while mutants show only 3 rudiments. In young adult mutants, we frequently observed only 3 proximal radial bones, not 4. The missing elements are on the posterior side of the fin and are the last to develop. Most *hnrnpul1/1l* mutants are missing both the fourth proximal radial and the seventh distal radial and fin rays adjacent to these bones.

Double loss-of-function of Wiskott–Aldrich syndrome-like-b (*waslb*) and the proto-oncogene *vav2* in zebrafish results in loss of pr4, similar to what we observe in *hnrnpul1/1l* mutants (Hawkins *et al.* 2021). Strikingly, gain-of-function mutations in the *waslb* and *vav2* result in supernumerary intermediate bones in the pectoral fin via upregulation of *hox11b*, showing that these 2 genes have high-level control of skeletal formation. The role of Wasl in limbs is conserved in mice where its loss leads to bone fusions in limbs. Mechanistically, Wasl interacts with Cdc42 and Arp2/3 in nucleating filamentous actin (F-actin) while Vav2 is a Rho GTPase that also activates Cdc42. Conservation between the roles of mouse and fish Wasl/waslb in actin organization and bone abnormalities points to actin organization as a contributor to limb phenotypes across species. Whether actin abnormalities underlie *hnrnpul1/1l* phenotypes is unknown. Mechanical tension, relying on the interactions between tendons and acto-myosin inside cells, shapes morphology of developing intramembranous bones (Galea *et al.* 2021). Functional changes in *hnrnpul1/1l* mutants due to smaller fins might mean there is decreased force during swimming, potentially resulting in altered bone shape and development.

Growth pathways downstream of splicing mutations can also contribute to skeletal defects. Mice with limb-specific deficiency in the minor spliceosomal protein U11 show growth abnormalities as well as digit condensation defects during development (Drake *et al.* 2020). Similar to *hnrnpul1/1l* zebrafish mutants, growth defects arise from reduced proliferation in U11 mutants. Proliferation defects in U11 mutants are due to changes in cell-cycle regulation downstream of minor-intron retention changes in splicing and result in fewer mesenchymal progenitors for cartilage and bone formation. Wolpert suggested that a threshold of cells need to be present for a rudiment to form (Wolpert *et al.* 1979). Similar to U11 mutants, insufficient proliferation and/or growth in *hnrnpul1/1l* mutants may mean that there are too few cells for some elements of the skeleton to form.

There are similarities between the zebrafish model and patients with *HNRNPUL1* VUS, but it is important to state that at this point, we cannot conclude that the patients’ *HNRNPUL1* variant is the cause of their limb anomalies, as our analysis is correlative. The affected individuals with the *HNRNPUL1* VUS have shortened limbs, rather than an absence of limbs, consistent with correct specification, but downstream growth defects *hnrnpul1* mutants. The patients have variable loss and shortening of bones with some limbs unaffected and others severely affected—we show variable loss of bones in the pectoral fins of the radials and rays. Overall, both patients show shortening of elements of the limb zeugopod (humerus and fibula) and variable agenesis of elements in the stylopod (ulna and tibia). Zebrafish do not have correlates of these bones; however, mesenchymal outgrowth that forms the fins and limbs (including bones) occurs via a homologous process.

### Caudal scoliosis in *hnrnpul1/1l* mutants

Scoliosis is defined as a 3D rotation of the spine to an angle greater than 10°. Congenital scoliosis is present at birth arising due to a developmental abnormality, while idiopathic scoliosis (IS) develops during childhood or adolescence with no known cause (Goldstein and Waugh 1973). The etiology of IS remains unknown but it does not appear to be due to vertebral abnormalities (Wajchenberg *et al.* 2016). Neurological, muscular, growth, and even hormonal abnormalities may be associated with IS [reviewed in Latalski *et al.* (2017)]. The fact that concordance is much higher in monozygotic twins than dizygotic twins does suggest a genetic link (Kesling and Reinker 1997). GWAS studies implicate genes involved in the formation of cartilaginous tissues of the spine. Modeling one of these genes in mice suggests that at least some human IS has roots in genes involved in spinal cartilage development (Bagnat and Gray 2020). Human IS has been difficult to study in mammalian models because common models such as mouse are quadrupeds and show a difference in spine structure and gravitational load (Ouellet and Odent 2013). The zebrafish has recently emerged as an excellent model for IS, due to a similar cranial to caudal spinal load and the ease of genetic manipulation, which makes them susceptible to late-onset spinal curvatures (Gorman and Breden 2009; Roy 2021).

A genetic screen for mutants with scoliosis in zebrafish has identified 5 groups of scoliosis mutants depending on location of scoliosis and vertebral structure among other factors (Gray *et al.* 2021). Scoliosis in *hnrpul1/1l* mutants arises in late larval or adult stages and is restricted to the caudal spine—most similar to the described Group IV scoliosis mutants. One gene that has been characterized causing scoliosis in Group IV is *adamts9* (Gray *et al.* 2021). *adamts9* mutants have structural defects in the central canal while the Reissner fiber is normal. We do not know if a similar mechanism could be at play in *hnrnpul1/1l* mutants.

Cerebrospinal fluid (CSF) flow in the CSF-containing central canal is important for normal spine development. Mutations in *ptk7*, a regulator of Wnt signaling required for cilia motility cause scoliosis (Hayes *et al.* 2014; Grimes *et al.* 2016; Van Gennip *et al.* 2018). CSF circulation is important for circulation of neurotensin neuropeptides (Zhang *et al.* 2018). Defects in mechanosensation by Pkd2l1 also lead to IS (Sternberg *et al.* 2018) as do defects in the Reissner fiber, that is suspended in the CSF. Scoliosis developed late in *hnrnpul1/1l*, so the RNAseq we undertook at 3 dpf may have been too early to detect changes relevant to motile cilia and CSF circulation. However, we did find a decrease in the expression of *arl3l2*. ARL3 proteins are present in the cilia and mutations lead to Joubert syndrome in humans (Alkanderi *et al.* 2018; Powell *et al.* 2021). Whether *arl3l2* plays a role in cilia activity in the zebrafish is currently unknown but may provide a mechanistic link between *hnrnpul1/1l* and scoliosis.

Disruption of pathways involved in embryonic muscle development may cause late-onset scoliosis (Lleras-Forero *et al.* 2020). Differences in fast-muscle area and fiber size are seen in *her1/her7* mutant embryos at 32 hpf. These mutants appear to recover and are normal throughout larval stages, but scoliosis appears with aging. Muscle defects are seen in the vicinity of scoliotic lesions. Weakness in muscles that stabilize the spine could allow it to curve through aging (Lleras-Forero *et al.* 2020). We did not detect severe muscle defects in *hnrnpul1/1l* mutants at 32 or 72 hpf, however, we did not test muscle function. Whether caudal scoliosis in *hnrnpul1/1l* mutants is caused or exacerbated by abnormal muscle function remains to be explored.

### Hnrnpul1 roles in craniofacial tendon development

*hnrnpul1/1l* mutants have an increased frequency of a craniofacial phenotype of an open jaw. Despite apparent displacement of the craniofacial skeleton, there are no morphological defects in craniofacial skeletal development. We thus examined the development of tendons and muscle. Zebrafish craniofacial bone, cartilage, and tendons share patterning and developmental pathways with mammalian tendons and are derived from the neural crest. Developing tendons require expression of *scleraxis (scxa)* for specification, followed by expression of differentiation markers *tenomodulin (tnmd)* and *collagen (col1a)*, and *thrombospondin 4b* (*tbsp4b*; Chen and Galloway 2014). Muscle and tendon development is coupled—muscle attachment is required for the normal maturation of craniofacial tendons (Chen and Galloway 2014) and mechanical load is needed for their differentiation (Brunt *et al.* 2017). Zebrafish mutations leading to a disruption of craniofacial tendon differentiation have been linked to jaw closure defects (Mcgurk *et al.* 2017). Zebrafish embryos that are anesthetized to prevent movement, show reduced jaw muscle activity and reduced tendon cell numbers in the jaw via reduced Wnt16 activity (Brunt *et al.* 2017).

In *hnrnpul1/1l* mutants, we observe a shorter sternohyoideus tendon that connects the sternohyoideus muscle to the hyohyal junction at the second pharyngeal arch midline using the *scxa* marker (Mcgurk *et al.* 2017). This region is referred to as the mandibulohyoid junction and is important for jaw opening. We find that tendons outside of this junction (Adductor Mandibulae or Palatoquadrate tendons) show no difference in size, consistent with a specific defect in jaw-tendon development, as opposed to tendon development in general. A shorter sternohyoideus tendon would hold the mandible open. Staining with Thsp4b, a protein essential for muscle attachment at myotendinous junctions, shows that the sternohyoideus tendon is present in mutants at 3 dpf and that all muscles and tendons of the head in *hnrnpul1/1l* mutants are present and appear to be correctly connected, however, decreased sternohyoideus tendon length in *hnrnpul1/1l* mutants could lead to the open jaw phenotype.

### Hnrnpul1 regulation of translation, growth, and cell cycle genes

IPA identified the major pathways affected in Hnrnpul1 mutants as “EIF2 signaling,” “EIF4 and p706SK signaling,” “Kinetochore Metaphase signaling,” “Protein ubiquitination,” and “DNA Damage 13-3-3δ.” In all cases, multiple genes in these pathways were upregulated ∼1,5- to ∼2-fold in mutants at the RNA level. Our sequencing was conducted on whole animals, and thus there may be higher differential expression if we looked at single tissues such as the fin. In addition to upregulated ribosomal and growth pathways, we also observed some important genes for growth and morphogenesis that were downregulated 3- to 4-fold in mutants including *egr1* and *egr4*. At the same time, genes involved in polyubiquitination were also upregulated. Therefore, the loss of *hnrnpul1* leads to a complex set of changes in gene regulation that would be predicted to have opposing effects on growth.

The upregulation of protein ubiquitination, specifically proteins involved in polyubiquitination, suggests an upregulation of the proteosome and protein degradation. Similarly, changes in the relative proportions of ribosomal subunits can lead to translational dysregulation and reactive oxygen species production, leading to DNA damage (Kampen *et al.* 2020). Increases in expression of EIF4BPs inhibit translation of muscle-specific genes, inhibiting muscle fiber growth (Yogev *et al.* 2013). We observe increased expression of EIF4BP1 and EIF4BP2 in *hnrnpuul1/1l* mutants that would be predicted to lead to decreased translation. DNA damage proteins were also upregulated in our RNAseq analysis. Thus, our analysis paints a picture of dysregulated proteostasis in *hnrnpul1/1l* mutants, potentially leading to changes in growth and other cellular functions.

There are increases in the transcription of cyclins and cyclin-dependent kinases in *hnrnpul1/1l* mutants. While this might result in increased cell division, this is not reflected in the in vivo phenotype, at least at the level of the fin where proliferation is decreased. It is possible that upregulation of these cyclins is compensatory, but not sufficiently compensatory to increase growth to normal levels.

In terms of developmental regulators, members of the FGF, Nodal, Notch, and Sonic Hedgehog signaling effectors (*fgf11*, *gdf3*, *her15/hes5*, and *hhatl1*) are downregulated in *hnrnpul1* mutants. Gdf3 is a Nodal ligand important for mesodermal and endodermal tissues. Embryonic morphological phenotypes observed in *gdf3* mutants resemble the low-frequency morphological defects we observe in *hnrnpul1* mutants (Bisgrove *et al.* 2017; Pelliccia *et al.* 2017).

### Hnrnpul1/1l modulates AS

Homology to other HNRNP genes suggested that HNRPUL1 might play a role in AS, but this has never been demonstrated directly. We provide the first evidence for its role in AS in zebrafish by demonstrating alternative exon usage, intron retention, and use of alternative 3′ and 5′ splice sites in *hnrnpul1/1l* mutants. Some of these might have phenotypic consequences. For instance, *hnrnpul1/1l* mutants are smaller at larval stages. Some alternatively spliced genes in *hnrnpul1/1l* mutants are associated with short stature in humans including *PUS7* (de Brouwer *et al.* 2018), *CHD4A* (Weiss *et al.* 2016), and *FBXL3* (Ansar *et al.* 2019). CHD4 is also a chromodomain helicase DNA-binding protein that participates in DNA repair (Pan *et al.* 2012). Human HNRNPUL1 is directly involved in DNA repair (Polo *et al.* 2012). Whether DNA repair defects contribute to any of the phenotypes in embryonic or adult zebrafish *hnrnpul1/1l* mutants should be investigated. A large-scale zebrafish screen identified *telemetric repeat factor a* (*terfa*) mutants as having a protruding jaw phenotype (Golling *et al.* 2002). *terfa* has altered AS in *hnrnpul1/1l* mutants and could be contributing to the phenotype in *hnrnpul1/1l* mutants.

In conclusion, loss of Hnrnpul1, a broadly expressed regulator of transcription and AS, has a developmental phenotype affecting multiple genes and tissues. Defective embryonic growth, coupled with even further reduced fin-mesenchymal proliferation, may be connected to developmental patterning of fin/limb-bone elements in *hnrnpul1/1l* mutants. Known mechanisms affecting fin/limb growth include decreased mechanical load, defective actin remodeling leading to loss of fin bones, mutations affecting proliferation, and limb growth. Further analysis of these mutants will shed light on the importance of transcriptional and splicing regulation on embryonic development, and fin/limb growth in particular.

## Data availability

Zebrafish strains and reagents are available upon request. Supplementary Table 1 contains RNAseq data for alternatively spliced genes. Supplementary Table 2 contains differentially expressed genes. Supplementary Table 3 contains IPA pathway analysis. Gene expression data are available at NCBI GEO database under the accession GSE144754.

Supplemental material is available at *G3* online.

## Supplementary Material

jkac067_Supplementary_Table_S1

jkac067_Supplementary_Table_S2

jkac067_Supplementary_Table_S3

jkac067_Supplementary_Material
