# Supplementary material for: Hnrnpul1 controls transcription, splicing, and modulates skeletal and limb development in vivo
Source: G3 (Bethesda). 2022 Mar 23;12(5):jkac067. doi: 10.1093/g3journal/jkac067 (PMC9073674; doi:10.1093/g3journal/jkac067)
Supplement: jkac067_Supplementary_Table_S1 [file jkac067_supplementary_table_s1.pdf]

Blackwell et al., Table S1: AS events

Gene Name	Gene ID	EVENT	Affected exon/intron	COORD	LENGTH	FullCO	COMPLEX	hnrnpul1/1l mutant average	DKO1	DKO2	DKO3	WT average	WT1	WT2	WT3	dPSI
hnrnpul1l	ENSDARG00000079780	DreEX0005068	13	chr5:61150155-61150323	169	chr5:61144632,61150155-61150323,61151040	S	0	0	0	0	95.86666667	100	100	87.6	-95.87
mark4	ENSDARG00000024966	DreEX0005820	16	chr15:23694785-23694867	83	chr15:23692386,23694785-23694867,23702150+23702158	S	32.18666667	28.64	0	67.92	90.5	100	77.95	93.55	-58.31
ubn2b	ENSDARG00000100508	DreEX0082949	14	chr3:7954681-7954749	69	chr3:7961187,7954681-7954749,7954555	S	28.07	0	44.68	39.53	79.43	100	58.06	80.23	-51.36
slc25a20	ENSDARG00000040401	DreEX0072278	8	chr22:135530-135654	125	chr22:135395,135530-135654,136237	S	55.31666667	83.7	0	82.25	100	100	100	100	-44.68
ap2m1a	ENSDARG00000002790	DreEX0013740	8	chr2:10003553-10003567	15	chr2:10003782,10003553-10003567,9999392+9999407	MIC	18.32	23.84	0	31.12	60.09	75.66	65.75	38.86	-41.77
mprip	ENSDARG00000102081	DreEX0047254	intron 15-16	chr12:501924-504059	2136	chr12:498740,501924-504059,509803	S	5.69	17.07	0	0	46.49333333	42.31	52.17	45	-40.8
snap25b	ENSDARG00000058117	DreEX0074047	intron 4-5	chr17:12162810-12162924	115	chr17:12169135,12162810-12162924,12152958	C3	20.21	25.68	0	34.95	60.94	70.67	54.79	57.36	-40.73
bsg	ENSDARG00000019881	DreEX0017472	2	chr22:18908465-18908815	351	chr22:18904664,18908465-18908815,18910027	S	2.48	3.88	0	3.56	35.37666667	64.71	28.34	13.08	-32.9
ears2	ENSDARG00000103099	DreEX0003830	4	chr1:58451484-58451614	131	chr1:58455497,58451484-58451614,58446902+58446961	C3	63.12333333	58.67	55.7	75	89.14	86.6	100	80.82	-26.02
hm13	ENSDARG00000037846	DreEX0037727	intron 11-12	chr23:469529-469718	190	chr23:471555,469529-469718,468377	S	4.283333333	5.86	1.22	5.77	28.88666667	14.98	47.76	23.92	-24.61
cdc14b	ENSDARG00000021483	DreEX0020101	1	chr8:1156996-1157061	66	chr8:1164574,1156996-1157061,1156873	C1	26.35333333	25.56	25.59	27.91	45.77666667	53.51	49.49	34.33	-19.43
si:ch211-266g18.10	ENSDARG00000057903	DreEX0068102	intron 24-25	chr17:15466314-15466358	45	chr17:15466527,15466314-15466358,15466096+15466099	C1	0	0	0	0	17.14333333	24.35	15.04	12.04	-17.14
neb	ENSDARG00000032630	DreEX0050008	121	chr9:23016894-23016998	105	chr9:23019453,23016894-23016998,23016092	S	0	0	0	0	13.61666667	24.45	7.69	8.71	-13.62
fgr1	ENSDARG00000056504	DreEX6030881	6	chr1:15986731-15986835	105	chr1:15986962,15986731+15986393-15986835,15983264+15983C	ANN	97.28333333	98.49	100	93.36	81.85666667	79.19	80.8	85.58	15.42
terfa	ENSDARG00000039302	DreEX0078215	12	chr4:76611232-76611339	108	chr4:76611010,76611232-76611339,76611452	S	93.38	90.12	90.02	100	76.42333333	84.65	74.23	70.39	16.96
atxn2l	ENSDARG00000011597	DreEX0016244	22	chr3:15273851-15273899	49	chr3:15272104,15273851-15273899,15275431	S	27.43333333	19.63	37.11	25.56	8.35	14.1	7.77	3.18	19.08
derl2	ENSDARG00000042401	DreEX6013850	6	chr5:58336592-58336682	91	chr5:58335092,58336592-58336682,58338991	ANN	98.45	100	100	95.35	78.56666667	62.6	85.27	87.83	19.88
amph	ENSDARG00000007663	DreEX0012972	21	chr2:31914057-31914098	42	chr2:31916019,31914057-31914098,31911007	C3	95.86	100	100	87.58	74.89666667	63.82	78.6	82.27	20.96
brd3a	ENSDARG00000006527	DreEX0017295	9	chr21:18234026-18234094	69	chr21:18234454+18234469,18234026-18234094,18233160	S	67.2	66.91	69.08	65.61	44.43333333	36.38	51.11	45.81	22.77
atxn7l2b	ENSDARG00000056268	DreEX0016265	intron 9-10	chr8:25086221-25086291	71	chr8:25084913,25086221-25086291,25087235	S	27.54333333	25.8	36.08	20.75	4.533333333	13.6	0	0	23.01
tmcc1b	ENSDARG00000060954	DreEX0079251	intron 6-7	chr11:18032238-18032388	151	chr11:18033100,18032238-18032388,18030527	S	26.53	16.6	38.46	24.53	1.156666667	0	0	3.47	25.37
fam184a	ENSDARG00000014081	DreEX0031677	18	chr20:41887209-41887417	209	chr20:41883334,41887209-41887417,41902164	C1	34.71333333	32.1	40.98	31.06	5.873333333	0	5.62	12	28.84
mical3a	ENSDARG00000021979	DreEX0046459	31	chr18:10924514-10924564	51	chr18:10922220,10924514-10924564,10927410	C1	38.49333333	25.37	24.2	65.91	8.933333333	8.1	0	18.7	29.56
fbxl3b	ENSDARG00000021882	DreEX6024787	4	chr6:60007247-60007418	172	chr6:60007833,60007247-60007418,60004605	ANN	100	100	100	100	69.12333333	51.18	64.76	91.43	30.88
madd	ENSDARG00000003495	DreEX0044312	36	chr7:34053012-34053081	70	chr7:34056528,34053012-34053081,34050479	C1	94.23	100	100	82.69	41.18	5.77	73.05	44.72	53.05
neil1	ENSDARG00000018061	DreINT0103628	5-6	chr25:26386542-26386679	138	chr25:26386680-26386773=26386420-26386541:-	IR	0	0	0	0	51.08333333	56.99	51.9	44.36	-51.08
dlc	ENSDARG00000002336	DreINT0057467	6-7	chr15:20530052-20530389	338	chr15:20530390-20530606=20529358-20530051:-	IR	19.96333333	18.86	0	41.03	68.39	48.65	100	56.52	-48.43
las1l	ENSDARG00000062457	DreINT0088464	11-12	chr5:21615475-21617542	2068	chr5:21617543-21617862=21615324-21615474:-	IR	0	0	0	0	37.65333333	28.38	76.67	7.91	-37.65
fam214b	ENSDARG00000068650	DreINT0066618	7-8	chr5:18522054-18524081	2028	chr5:18524082-18524240=18521985-18522053:-	IR	0.463333333	0	0	1.39	28.56333333	52.17	15.72	17.8	-28.1
snx27b	ENSDARG00000016977	DreINT0154148	12-13	chr16:23608849-23608954	106	chr16:23608800-23608848=23608955-23609342:+	IR	0	0	0	0	24.45666667	47.95	17.09	8.33	-24.46
CABZ01084323.1	ENSDARG00000104937	DreINT0005264	18-19	KN150000.1:37877-40116	2240	KN150000.1:37667-37876=40117-40277:+	IR	0	0	0	0	24.40333333	28.42	33.61	11.18	-24.4
pus7	ENSDARG00000031774	DreINT0122857	13-14	chr25:219504-219787	284	chr25:219402-219503=219788-219917:+	IR	0	0	0	0	24.4	19.2	48.55	5.45	-24.4
isl1	ENSDARG00000004023	DreINT0082688	3-4	chr5:40130533-40131949	1417	chr5:40131950-40132209=40130246-40130532:-	IR	0	0	0	0	16.51333333	10.48	33.9	5.16	-16.51
chmp1b	ENSDARG00000099624	DreINT0046566	8-9	chr5:22523710-22523837	128	chr5:22523653-22523709=22523838-22524308:+	IR	36.44333333	34.08	36.52	38.73	51.54	56.18	51.63	46.81	-15.1
tchp	ENSDARG00000035605	DreINT0161324	11-12	chr5:19375036-19375135	100	chr5:19374892-19375035=19375136-19375452:+	IR	0	0	0	0	14.83333333	8.3	18.72	17.48	-14.83
ndufb2	ENSDARG00000045490	DreINT0103274	3-4	chr4:22761470-22761553	84	chr4:22761380-22761469=22761554-22762156:+	IR	0.466666667	0.51	0	0.89	14.55	7	27.94	8.71	-14.08
prc1a	ENSDARG00000100918	DreINT0119190	13-14	chr25:7282830-7284550	1721	chr25:7282671-7282829=7284551-7284985:+	IR	0	0	0	0	13.42	11.57	12.16	16.53	-13.42
abce1	ENSDARG00000007216	DreINT0024248	9-10	chr23:43905513-43905596	84	chr23:43905597-43905686=43905391-43905512:-	IR	0	0	0	0	13.30666667	7.44	27.27	5.21	-13.31
CABZ01066312.1	ENSDARG00000100624	DreINT0004711	6-7	chr4:73710365-73710889	525	chr4:73710890-73711064=73710209-73710364:-	IR	0	0	0	0	12.99333333	10.92	21.39	6.67	-12.99
vldlr	ENSDARG00000006257	DreINT0173861	17-18	chr10:15346010-15346130	121	chr10:15345840-15346009=15346131-15347025:+	IR	13.02666667	14.78	13.93	10.37	25.99	27.42	25.91	24.64	-12.96
CABZ01111915.1	ENSDARG00000104583	DreINT0005516	1-2	KN149798.1:73-259	187	KN149798.1:34-72=260-475:+	IR	15.02666667	13.22	11.4	20.46	2.076666667	2.08	3.61	0.54	12.95
chd4a	ENSDARG00000063535	DreINT0046095	30-31	chr19:5101861-5103136	1276	chr19:5103137-5103270=5101716-5101860:-	IR	14.43666667	25.63	12.3	5.38	0	0	0	0	14.44
klhdc4	ENSDARG00000100206	DreINT0086909	1-2	chr25:12633465-12633552	88	chr25:12633553-12633755=12633373-12633464:-	IR	14.49	25.17	11.97	6.33	0	0	0	0	14.49
vapb	ENSDARG00000070435	DreINT0173431	3-4	chr6:49629863-49634335	4473	chr6:49629759-49629862=49634336-49634416:+	IR	15.70333333	10.92	31.15	5.04	0	0	0	0	15.7
actr3	ENSDARG00000100510	DreINT0008499	6-7	chr9:56884401-56885058	658	chr9:56884285-56884400=56885059-56885117:+	IR	99.61333333	99.58	100	99.26	83.41666667	79.81	78.77	91.67	16.19
zfand2a	ENSDARG00000103511	DreINT0177839	9-10	chr3:42679832-42686700	6869	chr3:42679747-42679831=42686701-42687238:+	IR	23.72	14.07	42.11	14.98	3	9	0	0	20.72
epc1b	ENSDARG00000060054	DreINT0063555	14-15	chr2:43783415-43785365	1951	chr2:43783072-43783414=43785366-43786709:+	IR	21.19	24	30.77	8.8	0	0	0	0	21.19
si:ch211-13c6.2	ENSDARG00000027738	DreINT0136119	11-12	chr19:42479760-42479856	97	chr19:42479688-42479759=42479857-42479958:+	IR	21.96666667	39.75	14.8	11.35	0	0	0	0	21.97
cstf2	ENSDARG00000090788	DreINT0052862	13-14	chr14:40994757-40994915	159	chr14:40994916-40995049=40994611-40994756:-	IR	41.71	46.87	36.91	41.35	18.45666667	19.64	15.43	20.3	23.25
hnrnpd	ENSDARG00000059246	DreINT0078622	6-7	chr10:5001911-5004090	2180	chr10:5004091-5004246=5001806-5001910:-	IR	78.87666667	86.47	84.65	65.51	53.63666667	50.87	56.71	53.33	25.24
fbxo11a	ENSDARG00000061631	DreINT0067575	12-13	chr12:24730094-24731125	1032	chr12:24731126-24731343=24729913-24730093:-	IR	30.72	40.21	42.86	9.09	0	0	0	0	30.72
col9a1b	ENSDARG00000031483	DreINT0050507	31-32	chr13:39004380-39004494	115	chr13:39004495-39004539=39004347-39004379:-	IR	37.64	33.33	21.05	58.54	6.27	0	13.25	5.56	31.37
gpatch4	ENSDARG00000091931	DreINT0074113	6-7	chr16:29092939-29094594	1656	chr16:29092888-29092938=29094595-29096051:+	IR	33.19666667	46.03	12.26	41.3	0	0	0	0	33.2
hnrnpa1a	ENSDARG00000011020	DreINT0078583	7-8	chr11:2071270-2072814	1545	chr11:2072815-2072898=2071096-2071269:-	IR	46.67333333	41.97	72.09	25.96	11.62	11.39	15.58	7.89	35.05
hspa9	ENSDARG00000003035	DreINT0079371	3-4	chr14:11838714-11841344	2631	chr14:11838626-11838713=11841345-11841526:+	IR	41.88	36.84	41.18	47.62	4.856666667	5.14	0	9.43	37.02
timm21	ENSDARG00000071594	DreINT0021122	5-6	chr24:16002789-16002868	80	chr24:16002683-16002788=16002869-16003286:+	IR	45.21666667	22.46	92.23	20.96	2.65	0	0	7.95	42.57
zranb2	ENSDARG00000044332	DreINT0181832	7-8	chr6:37327302-37327764	463	chr6:37327144-37327301=37327765-37327824:+	IR	59.85666667	35.11	93.37	51.09	1.21	0	0	3.63	58.65
emc4	ENSDARG00000102733	DreINT0009895	2-3	KN150334.1:15644-15701	58	KN150334.1:15702-15742=15514-15643:-	IR	96.76333333	100	100	90.29	12.11	9.69	15.92	10.72	84.65
pdss1	ENSDARG00000017910	DreALTD0013773-3/3	7	chr24:5989325-5989438	7	chr24:5989332+5989329+5989325-5989438,5989239	Alt5	0	0	0	0	61.72	100	60	25.16	-61.72
grinab	ENSDARG00000052746	DreALTD0008955-5/6	7	chr16:53629088-53629309	10	chr16:53629088-53629299+53629301+53629303+53629307+5362	Alt5	22.72333333	38.28	0	29.89	81.47333333	95.68	79.17	69.57	-58.75
sppl2	ENSDARG00000030448	DreALTD0019479-4/4	14	chr2:37515381-37515563	75	chr2:37515381-37515488+37515491+37515538+37515563,37519:	Alt5	18.79333333	36.25	0	20.13	73.71666667	64.06	70.33	86.76	-54.93
prrc2b	ENSDARG00000079639	DreALTD0001691-2/3	39	chr5:71239237-71239449	123	chr5:71239360+71239237+71239213-71239449,71232792	Alt5	32.19333333	38.04	32.9	25.64	52.32666667	45.41	57.84	53.73	-20.14
sub1a	ENSDARG00000053446	DreALTA0028751-2/3	2	chr10:8132968-8133064	27	chr10:8132552,8132995+8132968+8132950-8133064	Alt3	15.1	14.95	13.51	16.84	33.55333333	30.63	24.37	45.66	-18.45
rbm17	ENSDARG00000031346	DreALTA0022475-2/2	10	chr4:25513862-25513938	3	chr4:25516722,25513862-25513938+25513935	Alt3	58.63	62.83	50	63.06	76.47666667	80.77	71.74	76.92	-17.85
cmss1	ENSDARG00000004727	DreALTA0008140-2/3	4	chr9:30179208-30179340	3	chr9:30178870,30179211+30179208+30179205-30179340	Alt3	2.076666667	0	6.23	0	18.74666667	15.84	19.23	21.17	-16.67
cmss1	ENSDARG00000004727	DreALTA0008140-3/3	4	chr9:30179205-30179340	6	chr9:30178870,30179211+30179208+30179205-30179340	Alt3	97.92333333	100	93.77	100	81.25333333	84.16	80.77	78.83	16.67
prrc2b	ENSDARG00000079639	DreALTD0001691-3/3	39	chr5:71239213-71239449	147	chr5:71239360+71239237+71239213-71239449,71232792	Alt5	67.80666667	61.96	67.1	74.36	47.67333333	54.59	42.16	46.27	20.14
bcl7a	ENSDARG00000052907	DreALTD0004136-3/3	4	chr5:41194954-41195127	18	chr5:41194954-41195109+41195124+41195127,41195218	Alt5	97.27	100	100	91.81	72.81	79.1	68.6	70.73	24.46
nrxn1a	ENSDARG00000061647	DreALTD0012982-2/2	21	chr12:24507647-24507728	9	chr12:24507647-24507719+24507728,24514162	Alt5	55.81666667	46.26	63.71	57.48	29.06	36.86	24.76	25.56	26.76
SYT2	ENSDARG00000014169	DreALTA0003051-2/2	3	chr6:55174844-55174975	9	chr6:55166453,55174853+55174844-55174975	Alt3	61.71333333	81.82	53.32	50	33.57333333	23.4	33.57	43.75	28.14
grm8a	ENSDARG00000077654	DreALTA0013189-2/2	10-11	chr4:3727378-	52	chr4:3721213,3727430+3727378-	Alt3	82.04333333	100	90.91	55.22	37.87666667	50	30.3	33.33	44.16
onecut1	ENSDARG00000007982	DreALTD0013229-2/2	1	chr18:-38775345	15	chr18:-38775330+38775345,38778737	Alt5	89.44	100	100	68.32	43.16333333	56.81	34.48	38.2	46.28
max	ENSDARG00000024844	DreALTA0016534-2/3	4	chr20:28931343-28931481	15	chr20:28932847,28931343-28931502+28931481+28931466	Alt3	75.53666667	62.41	100	64.2	25.68	14.44	16.67	45.93	49.86
sppl2	ENSDARG00000030448	DreALTD0019479-2/4	14	chr2:37515381-37515491	3	chr2:37515381-37515488+37515491+37515538+37515563,37519:	Alt5	81.20666667	63.75	100	79.87	26.28333333	35.94	29.67	13.24	54.93
grinab	ENSDARG00000052746	DreALTD0008955-2/6	7	chr16:53629088-53629301	2	chr16:53629088-53629299+53629301+53629303+53629307+5362	Alt5	68.71666667	43.8	93.21	69.14	0	0	0	0	68.72
pdss1	ENSDARG00000017910	DreALTD0013773-2/3	7	chr24:5989329-5989438	3	chr24:5989332+5989329+5989325-5989438,5989239	Alt5	100	100	100	100	30.94666667	0	18	74.84	69.05
