# Supplementary material for: Hnrnpul1 controls transcription, splicing, and modulates skeletal and limb development in vivo
Source: G3 (Bethesda). 2022 Mar 23;12(5):jkac067. doi: 10.1093/g3journal/jkac067 (PMC9073674; doi:10.1093/g3journal/jkac067)
Supplement: jkac067_Supplementary_Table_S2 [file jkac067_supplementary_table_s2.pdf]

Blackwell et al., Table S2: Gene expression changes

NAME	ID	DKO average	WT average	FC	Log2(FC)	p-value	(-log10pval)
aagab	ENSDARG00000041170	12.01666667	16.83666667	0.71372	-0.4865698	0.0042	2.376735264
aars	ENSDARG00000069142	34.63666667	47.51	0.72904	-0.4559311	0.049612	1.304409908
abca3b	ENSDARG00000100524	6.883333333	11.24333333	0.612215	-0.7078905	0.026001	1.585013004
abca4a	ENSDARG00000057169	0.923333333	6.406666667	0.144121	-2.7946505	0.021052	1.676705231
abca4b	ENSDARG00000062661	1.3	4.16	0.3125	-1.6780719	0.015134	1.820043501
abhd16a	ENSDARG00000078929	21.82	26.7	0.817228	-0.2911886	0.033841	1.470559703
abhd8b	ENSDARG00000076620	1.14	2.45	0.465306	-1.1037479	0.026079	1.583705692
abi1a	ENSDARG00000010155	17.73	12.87666667	1.376909	0.4614334	0.001286	2.890889719
abi3a	ENSDARG00000060072	3.133333333	5.876666667	0.533182	-0.9072998	0.022227	1.653113167
AC024175.17	ENSDARG00000082753	271.6833333	464.0166667	0.585503	-0.7722506	0.014607	1.835428104
AC024175.19	ENSDARG00000083046	62.76	116.7566667	0.537528	-0.8955877	0.017314	1.761590716
AC024175.4	ENSDARG00000080337	25433.27	45149.64667	0.56331	-0.8279977	0.030377	1.517459164
acaca	ENSDARG00000078512	4.9	6.516666667	0.751918	-0.4113525	0.004366	2.35995706
acad8	ENSDARG00000042658	4.3	8.526666667	0.5043	-0.9876452	0.024884	1.604071806
ACADSB (2 of 2)	ENSDARG00000098174	4.583333333	8.773333333	0.522416	-0.9367279	0.028015	1.552601788
adal	ENSDARG00000012986	4.566666667	2.1	2.174603	1.1207522	0.034416	1.463241668
adam23a	ENSDARG00000062323	11.40666667	20.68333333	0.551491	-0.8585915	0.005937	2.226435811
adcy2b	ENSDARG00000014588	1.496666667	3.336666667	0.448551	-1.1566546	0.038243	1.417449193
adcyap1r1a	ENSDARG00000105201	4.826666667	9.056666667	0.532941	-0.907953	0.02907	1.536551145
add3a	ENSDARG00000040874	8.906666667	5.146666667	1.73057	0.7912473	0.024394	1.612722808
adgra1	ENSDARG00000054177	3.016666667	6.573333333	0.458925	-1.1236699	0.001365	2.864744928
adgrb1a	ENSDARG00000075133	11.45	23.68333333	0.483462	-1.0485246	0.047853	1.320089655
adgrg1	ENSDARG00000027222	8.346666667	4.283333333	1.948638	0.9624662	0.018962	1.722106135
adi1	ENSDARG00000020448	25.12666667	18.22333333	1.378818	0.4634324	0.01418	1.848330592
adipor1b	ENSDARG00000042717	10.54	17.91666667	0.588279	-0.7654274	0.02742	1.561937128
adka	ENSDARG00000039429	23.79	30.57666667	0.778044	-0.3620759	0.047897	1.319694617
adprm	ENSDARG00000026090	2.536666667	1.57	1.615711	0.6921694	0.038156	1.418433138
AEBP1 (1 of 2)	ENSDARG00000006901	15.78	11.01	1.433243	0.5192827	0.021744	1.662653971
AES (3 of 3)	ENSDARG00000067832	6.503333333	10.79333333	0.602532	-0.7308892	0.037264	1.428713003
agbl5	ENSDARG00000045900	2.27	4.263333333	0.532447	-0.9092896	0.018458	1.733825328
agfg1b	ENSDARG00000019526	6.616666667	12.68333333	0.521682	-0.9387574	0.032747	1.484829824
agpat6	ENSDARG00000019897	16.53	24.31	0.679967	-0.5564632	0.02453	1.610305751
ahcyl2	ENSDARG00000039343	9.946666667	14.20333333	0.700305	-0.5139445	0.040844	1.388874318
ahnak	ENSDARG00000061764	42.11333333	28.58	1.473525	0.5592712	0.018482	1.733256412
aifm1	ENSDARG00000058088	13.86	20.69	0.669889	-0.5780064	0.014398	1.841685353
ak3	ENSDARG00000058226	15.40666667	11.74333333	1.31195	0.3917128	0.002086	2.680695615
ak5	ENSDARG00000012555	2.686666667	5.086666667	0.528178	-0.9209032	0.039993	1.398013211
akap12b	ENSDARG00000055678	49.17	33.85	1.452585	0.5386225	0.044883	1.347921532
akap1b	ENSDARG00000006062	1.916666667	4.186666667	0.457803	-1.1272026	0.004159	2.380996744
akt1s1	ENSDARG00000074667	65.20333333	44.61666667	1.461412	0.547363	0.004879	2.311646234
alas2	ENSDARG00000038643	6.95	2.56	2.714844	1.4408692	0.020179	1.695102971
alcamb	ENSDARG00000058538	38.67	26.16333333	1.478023	0.5636684	0.028712	1.541935641
aldh1l1	ENSDARG00000077004	13.55333333	10.11333333	1.340145	0.4223891	0.041137	1.385762919
aldh7a1	ENSDARG00000018426	29.59333333	37.18333333	0.795876	-0.3293839	0.039215	1.406548088
aldoaa	ENSDARG00000011665	314.4666667	281.68	1.116397	0.15885	0.021054	1.676671857
alg9	ENSDARG00000012840	15.26333333	19.25	0.7929	-0.3347884	0.04134	1.383633651
alkbh4	ENSDARG00000052247	5.533333333	8.683333333	0.637236	-0.6501001	0.047039	1.32754017
alox12	ENSDARG00000069463	7.9	4.426666667	1.784639	0.8356319	0.044598	1.350686797
ambra1b	ENSDARG00000039878	3.773333333	5.62	0.671412	-0.5747306	0.04204	1.376337881
amigo3	ENSDARG00000074469	0.37	2.183333333	0.169466	-2.5609352	0.019305	1.714334199
AMIGO3 (2 of 2)	ENSDARG00000079569	0.753333333	3.546666667	0.212406	-2.2351035	0.011289	1.9473435

amn1	ENSDARG00000070478	1.82	3.963333333	0.459209	-1.1227759	0.011227	1.949747279
amph	ENSDARG00000007663	19.71333333	43.19	0.456433	-1.1315256	0.008309	2.080433063
anapc11	ENSDARG00000102531	126.5133333	74.26666667	1.703501	0.7685027	0.006943	2.158446233
anapc16	ENSDARG0000001382	80.20333333	57.4	1.397271	0.4826115	0.046684	1.330828644
angptl2b	ENSDARG00000068369	8.76	5.12	1.710938	0.7747871	0.010498	1.978878767
ankfy1	ENSDARG00000061013	6.74	9.47	0.711721	-0.4906158	0.045007	1.346716549
ankra2	ENSDARG00000035399	53.38	36.31666667	1.469849	0.5556675	0.042327	1.373386842
ankrd13c	ENSDARG00000103831	18.05333333	29.80333333	0.605749	-0.7232085	0.023011	1.63806503
ANKRD34A	ENSDARG00000076719	1.523333333	2.603333333	0.585147	-0.7731284	0.007236	2.140479026
ankrd6a	ENSDARG00000057790	0.94	2.793333333	0.336516	-1.5712551	0.038377	1.415923594
ano1	ENSDARG00000102335	1.536666667	4.03	0.381307	-1.3909756	0.000816	3.088059951
ANO2 (1 of 3)	ENSDARG00000003210	0.603333333	2.793333333	0.21599	-2.2109605	0.032667	1.4858858
anp32e	ENSDARG00000054804	227.29	326.98	0.695119	-0.5246682	0.049835	1.302464588
ap1g1	ENSDARG00000102394	7.023333333	10.07666667	0.69699	-0.5207907	0.028963	1.538149958
ap1g2	ENSDARG00000054337	7.623333333	10.76666667	0.70805	-0.4980778	0.000408	3.389199868
apba1b	ENSDARG00000074328	14.54666667	25.76666667	0.564554	-0.8248173	0.004541	2.342843451
APBB3	ENSDARG00000053771	0.996666667	2.38	0.418768	-1.2557786	0.024955	1.60284939
apmap	ENSDARG00000017422	16.27333333	27.46666667	0.592476	-0.755172	0.003912	2.407585494
arf6a	ENSDARG00000101626	16.16666667	24.82333333	0.651269	-0.6186746	0.025094	1.600426278
arfgap3	ENSDARG00000097583	3.516666667	2.33	1.509299	0.5938786	0.022419	1.64937638
arfgef1	ENSDARG00000063474	5.763333333	11.21333333	0.513971	-0.9602398	0.000353	3.451635855
arfip2a	ENSDARG00000059244	29.18333333	40.48	0.720932	-0.4720646	0.034183	1.466194926
arhgap35	ENSDARG00000060297	4.61	7.37	0.625509	-0.6768979	0.038652	1.412832015
ARHGAP44 (1 of 2)	ENSDARG00000102424	0.913333333	2.473333333	0.369272	-1.4372433	0.025366	1.595745495
arhgdia	ENSDARG00000043795	49.99333333	77.50666667	0.64502	-0.6325847	0.028871	1.539534713
arih2	ENSDARG00000012848	23.20666667	29.82333333	0.778138	-0.3619022	0.02715	1.566231152
arl13a	ENSDARG00000052575	6.353333333	15.70666667	0.404499	-1.3057914	0.005546	2.256054968
arl3l2	ENSDARG00000015404	5.513333333	28.20666667	0.195462	-2.3550395	0.004116	2.385533393
arl4aa	ENSDARG00000099918	22.42	13.02666667	1.721085	0.7833183	0.041124	1.385901531
arl4ab	ENSDARG00000033182	3.326666667	6.163333333	0.539751	-0.8896335	0.034317	1.464494802
arl4cb	ENSDARG00000032708	33.45666667	22.74333333	1.471054	0.55685	0.002805	2.552112649
arl5c	ENSDARG00000035719	4.556666667	6.57	0.693557	-0.5279145	0.020444	1.689443567
arl8ba	ENSDARG00000006915	13.03	17.28333333	0.753905	-0.4075444	0.009915	2.00370671
arl8bb	ENSDARG00000070318	1.323333333	2.866666667	0.461628	-1.1151977	0.035032	1.455539782
arpc4	ENSDARG00000054063	68.43666667	48.27666667	1.417593	0.5034434	0.006311	2.199907768
arpc4l	ENSDARG00000058225	149.7866667	105.84	1.415218	0.5010242	0.005842	2.233427984
arr3a	ENSDARG00000056511	74.46666667	252.7133333	0.294669	-1.7628351	0.011761	1.929569813
arr3b	ENSDARG00000098475	42.03666667	103.49	0.406191	-1.2997712	0.0398	1.400115434
arrdc1b	ENSDARG00000079839	1.09	2.106666667	0.517405	-0.9506339	0.005701	2.244024029
arrdc2	ENSDARG00000020761	6.83	5.463333333	1.250153	0.3221041	0.034488	1.462326548
asb12a	ENSDARG00000017086	22.72	15.75666667	1.441929	0.5280005	0.00014	3.852448477
asf1ba	ENSDARG00000101037	20.22	15.2	1.330263	0.4117117	0.00096	3.017513695
asic1a	ENSDARG00000008329	3.126666667	5.08	0.615486	-0.7002031	0.035024	1.455635179
aspg	ENSDARG00000052942	4.243333333	5.923333333	0.716376	-0.4812113	0.00716	2.145094402
asphd1	ENSDARG00000075813	4.786666667	8.533333333	0.560938	-0.8340881	0.003013	2.521034264
asxl1	ENSDARG00000036956	12.62	14.81	0.852127	-0.2308597	0.009651	2.015447458
atg12	ENSDARG00000069545	25.41	13.73	1.850692	0.8880648	0.032278	1.491090728
atg4c	ENSDARG00000044373	9.163333333	6.176666667	1.48354	0.569044	0.002738	2.562641958
atg4da	ENSDARG00000043548	9.773333333	11.19	0.873399	-0.1952874	0.019621	1.707284348
atoh1a	ENSDARG00000055294	6.24	2.33	2.678112	1.4212161	0.028771	1.541042166
ATP11B	ENSDARG00000101094	5.893333333	8.62	0.683681	-0.548604	0.027092	1.567163695
atp1b1b	ENSDARG00000076833	4.9	14.25	0.34386	-1.5401083	0.044016	1.356393941
atp1b2b	ENSDARG00000034424	10.05	27.56666667	0.364571	-1.4557293	0.030342	1.51794944
atp2b1b	ENSDARG00000007788	12.81	29.93333333	0.427951	-1.2244825	0.001185	2.926136114

atp2b2	ENSDARG00000063433	2.726666667	6.316666667	0.431662	-1.2120251	0.033475	1.475274869
atp5g1		294.8533333	401.33	0.73469	-0.4447915	0.001943	2.711423339
atp5g3a		160.9266667	216.7766667	0.742362	-0.4298061	0.028558	1.544273114
atp6ap1a	ENSDARG00000041417	34.95	72.5	0.482069	-1.0526885	0.011126	1.953680063
ATP6AP1L (1 of 2)	ENSDARG00000090963	10.77666667	21.78333333	0.494721	-1.0153137	0.002243	2.649136008
atp6v0cb	ENSDARG00000036577	170.2933333	371.8333333	0.457983	-1.1266342	0.01165	1.933680458
atp6v0d1	ENSDARG00000069090	77.83333333	98.60333333	0.789358	-0.3412483	0.021977	1.658030588
atp6v1aa	ENSDARG00000034534	49.24	93.37666667	0.527327	-0.9232313	0.016432	1.784315517
atp6v1ba	ENSDARG00000013443	56.26333333	91.71333333	0.61347	-0.7049365	0.001052	2.978099924
atp6v1d	ENSDARG00000011175	47.83	88.12666667	0.542742	-0.8816628	0.008541	2.068489356
atp6v1e1b	ENSDARG00000030694	151.0633333	210.11	0.718973	-0.4759913	0.049092	1.308987803
atp6v1g1	ENSDARG00000022315	299.7166667	410.77	0.729646	-0.4547315	0.01574	1.803001097
atp8a2	ENSDARG00000077492	4.853333333	9.963333333	0.487119	-1.0376525	0.012192	1.913915299
atrnl1b	ENSDARG00000006420	10.52666667	12.48666667	0.843033	-0.2463397	0.010352	1.984960473
atxn7l2a	ENSDARG00000055300	5.516666667	8.623333333	0.639737	-0.6444488	0.0343	1.464707027
atxn7l3	ENSDARG00000029331	1.736666667	4.29	0.404817	-1.3046568	0.039032	1.408580652
azin1b	ENSDARG00000035869	109.7566667	171.4033333	0.640341	-0.6430866	0.00212	2.673669272
BAALC	ENSDARG00000089549	6.67	10.43333333	0.639297	-0.6454415	0.010165	1.992878338
babam1	ENSDARG00000077526	13.00333333	20.12333333	0.646182	-0.6299878	0.027172	1.565871638
bahd1	ENSDARG00000086789	2.236666667	3.72	0.601254	-0.7339524	0.005754	2.240018684
BAHD1 (2 of 2)	ENSDARG00000102965	0.806666667	2.693333333	0.299505	-1.7393482	0.005178	2.285836086
bambib	ENSDARG00000099677	4.696666667	8.576666667	0.54761	-0.8687798	0.013353	1.874425835
bbc3	ENSDARG00000069282	1.176666667	2.646666667	0.444584	-1.1694708	0.012515	1.902583123
bbs5	ENSDARG00000039827	7.85	15.05333333	0.521479	-0.9393184	0.016259	1.788895306
bbx	ENSDARG00000012699	7.36	12.38	0.594507	-0.7502336	5.77E-05	4.239157452
bc2		50.59333333	38.48333333	1.314682	0.3947135	0.006153	2.210941867
bcat1	ENSDARG00000045568	16.82333333	28.54	0.589465	-0.7625217	0.023427	1.630287232
bckdhb	ENSDARG00000014676	3.466666667	7.08	0.489642	-1.0302002	0.004594	2.337834062
bckdk	ENSDARG00000016904	28.64	39.11333333	0.732231	-0.449629	0.010356	1.984798419
BEGAIN	ENSDARG00000043673	3.243333333	8.393333333	0.386418	-1.3717666	0.003683	2.433757031
bet1l	ENSDARG00000032037	2.516666667	6.363333333	0.395495	-1.3382686	0.038475	1.414819254
bhlhe23	ENSDARG00000037588	39.33666667	66.08666667	0.595228	-0.7484845	0.021191	1.673841412
birc5a	ENSDARG00000075621	19.83333333	11.46666667	1.729651	0.7904811	0.019451	1.71106503
bnip3	ENSDARG00000099961	10.66666667	15.71333333	0.678829	-0.5588799	0.030502	1.515667731
brd2a	ENSDARG00000022280	16.65	24.23333333	0.68707	-0.5414707	0.003311	2.479988049
BRD8 (2 of 2)	ENSDARG00000055999	2.613333333	4.323333333	0.604472	-0.7262529	0.049232	1.307752444
brk1	ENSDARG00000041974	103.8633333	148.1033333	0.70129	-0.5119177	0.007826	2.10643585
bscl2l	ENSDARG00000025912	29.51333333	11.87333333	2.485682	1.3136419	0.007134	2.146641156
bsg	ENSDARG00000019881	277.0166667	248.2666667	1.115803	0.1580822	0.027927	1.553976202
btbd2a	ENSDARG00000042091	14.2	23.62666667	0.601016	-0.7345252	0.004736	2.324633635
btbd6b	ENSDARG00000032369	24.07	31.04666667	0.775285	-0.3672022	0.001266	2.897514575
btc	ENSDARG00000098112	2.69	1.66	1.620482	0.6964229	0.039269	1.405949849
bub1ba	ENSDARG00000078825	5.023333333	2.673333333	1.879052	0.9100053	0.021313	1.671357707
bud31	ENSDARG00000017084	81.37	60.50333333	1.344885	0.4274824	0.048947	1.310274111
BX321873.1	ENSDARG00000098447	10.42333333	6.526666667	1.597038	0.6753985	0.00896	2.047675238
BX324179.3	ENSDARG00000103008	2.536666667	0.663333333	3.824121	1.935128	0.035419	1.450762692
BX510909.3		11.57	8.91	1.298541	0.3768915	0.003945	2.403943314
BX511246.1	ENSDARG00000100234	3.296666667	0	4.296667	2.1032179	0.000305	3.515251163
BX572103.7	ENSDARG00000104062	6.103333333	2.216666667	2.753383	1.4612055	0.00672	2.172600464
BX901974.1	ENSDARG00000067592	3.376666667	5.283333333	0.639117	-0.6458487	0.037757	1.423008013
BX927081.1	ENSDARG00000105300	0.97	4.3	0.225581	-2.14828	0.010025	1.998923898
BX957331.1	ENSDARG00000098380	162.4266667	249.5733333	0.650817	-0.6196753	0.034812	1.458276618
C13H10orf32		24.71333333	18.32	1.348981	0.4318701	0.043899	1.357542432
C13H6orf203	ENSDARG00000077614	32.80666667	21.54666667	1.522587	0.6065243	0.027512	1.56048523

C18H15orf59	ENSDARG00000026247	7.563333333	15.52666667	0.487119	-1.037654	0.022335	1.651020047
C1H3orf70 (2 of 2)	ENSDARG00000091513	6.646666667	9.573333333	0.69429	-0.5263903	3.39E-05	4.469798352
C1H4orf27		20.46666667	16.05333333	1.274917	0.3504033	0.012609	1.899313444
C1H4orf33	ENSDARG00000017985	18.78	10.16666667	1.847213	0.8853503	0.002599	2.585135391
C20H2orf44	ENSDARG00000016528	1.56	3.213333333	0.485477	-1.0425246	0.009718	2.012426645
C20H2orf71 (1 of 2	ENSDARG00000095802	1.323333333	5.27	0.251107	-1.9936265	0.01314	1.881409483
C20H2orf71 (2 of 2	ENSDARG00000102288	1.686666667	4.813333333	0.350416	-1.5128615	0.019741	1.704640255
C21H18orf54	ENSDARG00000071487	2.406666667	1.16	2.074713	1.0529115	0.043902	1.35751917
C21H5orf24 (2 of 2	ENSDARG00000104582	13.15	25.18	0.52224	-0.9372155	0.008352	2.078217164
C24H8orf33		7.56	5.76	1.3125	0.3923174	0.040972	1.387508221
C3H19orf43		40.02333333	32.22	1.242189	0.3128848	0.012499	1.903118795
C3H19orf52		29.76666667	21.41333333	1.3901	0.4751883	0.012127	1.916241157
C5H3orf30	ENSDARG00000093783	2.69	0.753333333	3.570796	1.8362459	0.048682	1.312633896
C5H9orf142	ENSDARG00000078807	15.45	8.583333333	1.8	0.8479969	0.025237	1.59795413
C5H9orf16		38.75333333	35.48	1.092259	0.1273144	0.011657	1.93339857
C8H9orf64		7.713333333	5.986666667	1.288419	0.3656015	0.002392	2.621157284
C9H2orf49	ENSDARG00000042025	9.713333333	15.13666667	0.641709	-0.6400092	0.032926	1.482462154
CA10 (1 of 2)	ENSDARG00000009568	1.956666667	4.093333333	0.478013	-1.0648782	0.021793	1.661684464
ca10a	ENSDARG00000052644	18.20666667	25.62666667	0.710458	-0.493179	0.00705	2.151834092
ca16b	ENSDARG00000060123	3.79	5.52	0.686594	-0.5424704	0.005399	2.267683948
cab39	ENSDARG00000043451	19.90666667	27.41666667	0.726079	-0.4618015	0.028861	1.539687121
CABZ01025393.1	ENSDARG00000044600	2.156666667	4.403333333	0.48978	-1.0297928	0.026707	1.573380857
CABZ01030107.1	ENSDARG00000058631	5.883333333	1.51	3.896247	1.9620852	0.001302	2.885271346
CABZ01041962.1	ENSDARG00000076082	12.28	5.413333333	2.268473	1.1817214	0.000447	3.34988383
CABZ01071911.1	ENSDARG00000090998	12.43666667	8.313333333	1.49599	0.5811009	0.009427	2.025648735
CABZ01088567.1	ENSDARG00000103065	2.25	7.203333333	0.312355	-1.6787397	0.044809	1.348636589
cacna1db	ENSDARG00000101589	0.646666667	4.903333333	0.131883	-2.9226687	0.002543	2.594646784
cadm3	ENSDARG00000057013	5.48	8.87	0.617813	-0.6947582	0.025902	1.586665401
cadm4	ENSDARG00000040291	13.39	37.86	0.353671	-1.4995185	0.022116	1.655294089
cadpsb	ENSDARG00000070567	16.53666667	26.64	0.620746	-0.6879256	0.004656	2.331947606
calb2a	ENSDARG00000041062	74.11666667	125.73	0.589491	-0.762459	0.046953	1.32833476
calb2b	ENSDARG00000036344	75.15	167.38	0.448978	-1.1552821	0.019412	1.71193083
calm1b	ENSDARG00000034187	124.3766667	252.3866667	0.492802	-1.0209198	0.01379	1.860431431
calub	ENSDARG00000005115	30.17666667	19.66333333	1.534667	0.6179255	0.032992	1.481596415
camk2d2	ENSDARG00000014273	18.75	30.72333333	0.610285	-0.7124442	0.038345	1.416295478
CAMK2N1	ENSDARG00000101387	25.98666667	49.22	0.52797	-0.9214731	0.02657	1.575604752
camkk1b	ENSDARG00000015134	0.566666667	2.906666667	0.194954	-2.3587934	0.018706	1.728015896
camkvb	ENSDARG00000005141	3.44	7.186666667	0.478664	-1.0629142	0.000666	3.17662377
CAMSAP3 (2 of 2)	ENSDARG00000091359	6.436666667	11.88666667	0.541503	-0.8849585	0.026876	1.570631693
camta1a	ENSDARG00000077428	3.346666667	7.056666667	0.474256	-1.076262	0.009172	2.037515313
cant1a	ENSDARG00000012192	11.53666667	7.77	1.48477	0.5702399	0.041268	1.384387169
caskb	ENSDARG00000100119	29.92333333	47.67333333	0.627674	-0.6719116	0.048822	1.311387769
CASKIN1 (2 of 2)	ENSDARG00000100166	10.07666667	16.35666667	0.616059	-0.6988603	0.049331	1.30687738
cat	ENSDARG00000104702	17.22333333	26.17	0.658133	-0.6035495	0.014042	1.852559259
cbln2b	ENSDARG00000077151	25.69666667	43.52666667	0.590366	-0.7603183	0.030901	1.51003139
cby1	ENSDARG00000077409	13.29666667	8.696666667	1.528938	0.6125302	0.034097	1.467284786
CCDC34 (2 of 2)	ENSDARG00000102725	13.50333333	3.696666667	3.65284	1.8690187	0.014674	1.833466118
ccdc47	ENSDARG00000054362	54.16666667	64.99666667	0.833376	-0.2629604	0.030889	1.510199448
ccdc53		14.97666667	12.87666667	1.163086	0.2179574	0.046747	1.330244871
ccdc85al	ENSDARG00000005343	4.236666667	7.25	0.584368	-0.7750514	0.046291	1.334507274
CCDC86	ENSDARG00000059908	76.94333333	44.70333333	1.721199	0.7834139	0.034774	1.458741822
ccdc94		15.40333333	18.30333333	0.841559	-0.2488638	0.037744	1.423153296
ccnb1	ENSDARG00000051923	35.50333333	23.53	1.508854	0.5934532	0.028168	1.55025011
ccnb2	ENSDARG00000036180	5.606666667	3.96	1.415825	0.5016429	0.011043	1.956921568

ccne2	ENSDARG00000098529	20.38666667	13.09333333	1.557026	0.6387935	0.012202	1.913571154
ccnt2b	ENSDARG00000036510	16.84666667	23.08666667	0.729714	-0.4545967	0.0192	1.716707498
cd151	ENSDARG00000038288	5.69	2.883333333	1.97341	0.980691	0.031569	1.500737795
cd36	ENSDARG00000032639	4.91	6.796666667	0.722413	-0.4691043	0.04856	1.31372328
cdc123	ENSDARG00000075025	19.13333333	9.99	1.915249	0.9375317	0.03238	1.489723951
cdc14ab	ENSDARG00000057016	10.85333333	8.713333333	1.245601	0.3168416	0.03397	1.468909275
cdc20	ENSDARG00000100741	16.82666667	10.87333333	1.547517	0.6299551	0.049774	1.303001193
cdc42ep4a	ENSDARG00000101457	6.473333333	8.87	0.729801	-0.4544253	0.033912	1.469643423
cdh6	ENSDARG00000014522	13.42	21.64666667	0.619957	-0.6897602	0.033554	1.474249264
CDHR1 (2 of 2)	ENSDARG00000104756	1.593333333	4.576666667	0.348143	-1.5222491	0.005016	2.299650029
cdhr1a	ENSDARG00000004643	1.17	6.033333333	0.193923	-2.3664468	0.014365	1.842691242
cdipt	ENSDARG00000070686	17.06666667	32.44333333	0.526045	-0.9267407	0.002636	2.579029812
cdk1	ENSDARG00000087554	52.33666667	39.31666667	1.331157	0.412681	0.034323	1.46441014
cdk12	ENSDARG00000063726	3.67	5.98	0.613712	-0.7043654	0.050022	1.300842232
cdk2	ENSDARG00000026577	21.07666667	14.03666667	1.501544	0.5864463	0.026282	1.5803414
cdk9	ENSDARG00000044811	29.62666667	39.26666667	0.754499	-0.4064088	0.037773	1.422823007
cdkl5	ENSDARG00000015240	4.223333333	8.953333333	0.471705	-1.0840428	0.007938	2.100290864
cdon	ENSDARG00000061328	19.45	15.28666667	1.272351	0.3474963	0.042022	1.376520525
cdr2l	ENSDARG00000026834	19.47	24.49333333	0.79491	-0.3311362	0.012695	1.896376711
celf5a	ENSDARG00000071375	3.233333333	7.52	0.429965	-1.2177104	0.016042	1.794731953
cenpk	ENSDARG00000039616	13.64666667	8.02	1.701579	0.7668745	0.015822	1.800744115
cenpw	ENSDARG00000092136	21.37666667	15.05	1.420377	0.5062734	0.022638	1.645159303
cep19	ENSDARG00000059175	2.08	4.113333333	0.505673	-0.9837245	0.005742	2.240913292
cep290	ENSDARG00000062727	3.413333333	6.103333333	0.559257	-0.8384161	0.003316	2.479351406
cep85	ENSDARG00000060215	5.313333333	3.43	1.549077	0.6314086	0.007603	2.119026631
ces3	ENSDARG00000041595	0.97	4.053333333	0.239309	-2.0630522	0.030863	1.510558599
cfp	ENSDARG00000094451	7.15	1.116666667	6.402985	2.6787446	3.56E-05	4.448373422
cgref1	ENSDARG00000075444	9.59	6.916666667	1.386506	0.4714539	0.024447	1.611779754
chac1	ENSDARG00000070426	102.9033333	185.0733333	0.556014	-0.8468073	0.012269	1.91118304
chd5	ENSDARG00000105083	5.693333333	10.30666667	0.552393	-0.8562323	0.043455	1.361957253
chm	ENSDARG00000003845	7.26	10.54333333	0.688587	-0.5382896	0.027068	1.567542951
chmp4ba	ENSDARG00000016255	24.58	33.45333333	0.734755	-0.4446651	0.048971	1.310065334
chmp5a	ENSDARG00000038855	7.05	10.96666667	0.642857	-0.6374299	0.001647	2.783318836
chn1	ENSDARG00000101735	38.80333333	54.50666667	0.711901	-0.4902521	0.043423	1.362277848
chodl	ENSDARG00000034528	2.963333333	7.75	0.382366	-1.3869754	0.012788	1.893202237
chordc1b	ENSDARG00000017067	20.16333333	23.94666667	0.84201	-0.2480907	0.006488	2.187856732
chp2	ENSDARG00000025788	14.96666667	8.336666667	1.795282	0.8442104	0.005098	2.292607345
chrnb3a	ENSDARG00000052764	7.666666667	13.01666667	0.588988	-0.7636887	0.041021	1.386997656
chst1	ENSDARG00000088593	14.77333333	18.37	0.80421	-0.3143562	0.025318	1.59656425
chst2b	ENSDARG00000058585	7.936666667	5.583333333	1.421493	0.5074065	0.021799	1.661572081
churc1	ENSDARG00000010831	53.65333333	39.58	1.355567	0.4388962	0.035344	1.451687904
ciao1	ENSDARG00000059212	33.60666667	20.95333333	1.603882	0.6815677	0.004219	2.374823619
cib2	ENSDARG00000102820	16.17666667	24.41	0.662707	-0.5935579	0.015507	1.809482576
cicb	ENSDARG00000055502	9.506666667	14.37	0.661563	-0.5960486	0.045306	1.343841826
cinp	ENSDARG00000073910	16.75	8.196666667	2.043514	1.0310519	0.007281	2.137781676
ckap2l	ENSDARG00000100573	7.2	4.253333333	1.69279	0.759403	0.032352	1.490099761
ckbb	ENSDARG00000043257	809.16	1093.59	0.739912	-0.434575	0.002364	2.626441469
ckmt2a	ENSDARG00000069615	54.17666667	136.3833333	0.397238	-1.3319238	0.024538	1.610153833
CKN149934.1H1orf		20.13	27.06666667	0.743719	-0.4271701	0.043654	1.359978197
CKN150339.1H9orf	ENSDARG00000102865	1.943333333	3.013333333	0.644912	-0.6328269	0.020899	1.679866389
cks1b	ENSDARG00000007971	40.14333333	24.40666667	1.644769	0.7178851	0.012593	1.89986296
cldn11a	ENSDARG00000020031	39.49666667	26.22333333	1.506165	0.5908798	0.02094	1.679017485
cldn19	ENSDARG00000044569	2.523333333	1.026666667	2.457792	1.2973629	0.006763	2.169846837
cldn5a	ENSDARG00000043716	7.516666667	4.6	1.634058	0.7084592	0.014285	1.845131154

cldnd1a	ENSDARG00000104439	13.67	23.21666667	0.588801	-0.7641476	0.028343	1.54755575
clgn	ENSDARG00000009315	27.84333333	35.81333333	0.777457	-0.3631649	0.029273	1.533530301
clstn1	ENSDARG00000031720	50.85	88.55	0.574252	-0.8002445	0.027427	1.561821445
clstn2	ENSDARG00000060637	2.943333333	6.563333333	0.448451	-1.1569778	0.008752	2.057895387
clstn3	ENSDARG00000073883	16.52	33.06666667	0.499597	-1.0011639	0.037618	1.424598882
clta	ENSDARG00000045618	123.5366667	188.3233333	0.655982	-0.6082725	0.013832	1.859112843
cltca	ENSDARG00000043493	26.37666667	39.55	0.66692	-0.5844154	0.015642	1.805693927
cltcb	ENSDARG00000090716	30.03666667	46.41333333	0.647156	-0.6278146	0.022635	1.645220473
cmya5	ENSDARG00000061379	3.93	1.023333333	3.840391	1.9412532	0.000823	3.084433149
cnbpa	ENSDARG00000045776	226.87	170.35	1.331787	0.4133639	0.033222	1.478568961
CNGB3 (2 of 2)	ENSDARG00000101368	2.716666667	13.45	0.201983	-2.3076967	0.012918	1.888821308
cnot3a	#N/A	10.90333333	15.17666667	0.718427	-0.4770857	0.004783	2.320258074
cnpy1	ENSDARG00000003757	18.61	11.52666667	1.614517	0.6911027	0.012077	1.918052887
cntn1b	ENSDARG00000045685	9.306666667	15.00333333	0.620307	-0.6889466	0.014686	1.833106024
cntnap5l	ENSDARG00000073802	1.25	3.013333333	0.414823	-1.2694322	0.000215	3.668163567
coa3		35.88	47.09	0.761945	-0.3922408	0.033997	1.468561093
coa7	ENSDARG00000019355	3.303333333	6.253333333	0.528252	-0.9207029	0.036744	1.43481276
commd7	ENSDARG00000036784	36.31333333	21.13333333	1.718297	0.780979	0.001568	2.804613798
commd8	ENSDARG00000028201	13.16666667	21.24333333	0.619802	-0.69012	0.003158	2.500572211
cops4	ENSDARG00000043732	41.91	52.05666667	0.805084	-0.3127884	0.037787	1.422658895
copz1	ENSDARG00000017844	148.3233333	112.1166667	1.322937	0.4037448	0.011723	1.930970168
coq3	ENSDARG00000069857	14.98333333	17.69666667	0.846675	-0.240119	0.02581	1.588209119
coro1ca	ENSDARG00000035598	28.53	19.82666667	1.438971	0.5250376	0.00051	3.292281923
coro2ba	ENSDARG00000079440	1.03	2.303333333	0.447178	-1.1610789	0.044069	1.355867031
cox4i1l	ENSDARG00000012388	12.66666667	3.156666667	4.012672	2.0045631	0.047191	1.326140216
COX5B (1 of 3)	ENSDARG00000011208	8.856666667	19.01	0.465895	-1.1019228	0.001794	2.746294353
cox6b1	ENSDARG00000045230	237.86	197.4833333	1.204456	0.2683818	0.01239	1.90693148
cox6c	ENSDARG00000038577	661.0333333	536.35	1.232466	0.3015483	0.024278	1.614781754
COX7A2 (2 of 2)	ENSDARG00000086341	140.32	78.48	1.787971	0.8383237	0.005258	2.279182223
cox8a	ENSDARG00000095273	292.7966667	411.7233333	0.711149	-0.4917761	0.045794	1.339195357
CPAMD8	ENSDARG00000056530	3.876666667	2.023333333	1.91598	0.9380827	0.044925	1.347513303
cpe	ENSDARG00000055874	99.59	133.8566667	0.744005	-0.4266162	0.006473	2.188917646
cplx3a	ENSDARG00000099196	0	3.163333333	0.240192	-2.0577391	0.049878	1.302092322
cpne4a	ENSDARG00000101095	5.653333333	9.57	0.590735	-0.7594172	0.042229	1.374388361
cpsf1	ENSDARG00000034178	11.44333333	8.676666667	1.318863	0.3992945	0.032101	1.493487196
cpt1aa	ENSDARG00000059770	14.18333333	11.03666667	1.28511	0.3618921	0.026526	1.576326493
CR388042.1	ENSDARG00000104048	8.973333333	3.463333333	2.590953	1.3734828	0.013126	1.881880509
CR974475.2	ENSDARG00000090107	3.69	1.183333333	3.11831	1.6407643	0.040472	1.39284437
crabp1b	ENSDARG00000035904	21.58666667	18.34333333	1.176813	0.2348847	0.045713	1.339963657
crabp2a	ENSDARG00000073978	44.72	20.91333333	2.138349	1.0964972	0.000752	3.123587927
creb3l3l	ENSDARG00000020926	30.18666667	41.41666667	0.728853	-0.4563	0.008703	2.060333855
crmp1	ENSDARG00000056742	18.14	40.52333333	0.447643	-1.1595784	0.014444	1.840320543
cry2a	ENSDARG00000069074	37.29333333	57.77333333	0.645511	-0.631486	0.017539	1.755988803
cry5	ENSDARG00000019498	1.956666667	3.456666667	0.566056	-0.8209835	0.031226	1.505483704
cry-dash	ENSDARG00000002396	5.013333333	8.14	0.615889	-0.6992586	0.020508	1.688087282
csf1a	ENSDARG00000075343	3.83	2.076666667	1.844302	0.8830747	0.04447	1.351931392
csnk1db	ENSDARG00000006125	26.11	39.96666667	0.653294	-0.6141948	0.009337	2.029808125
csnk2a2a	ENSDARG00000012818	11.96333333	19.46333333	0.61466	-0.7021394	0.016389	1.785456482
csnk2a2b	ENSDARG00000013582	11.61333333	22.89	0.507354	-0.9789353	0.049487	1.305505777
cspg5a	ENSDARG00000069981	65.13333333	112.55	0.578706	-0.7890981	0.041066	1.386522293
cst14a.2	ENSDARG00000045352	212.8033333	135.8	1.567035	0.6480373	0.016236	1.789527268
ctage5	ENSDARG00000099973	9.666666667	16.16666667	0.597938	-0.7419318	0.046438	1.33312993
CTBP1	ENSDARG00000019213	17.85666667	27.47	0.650042	-0.6213941	0.003603	2.443326823
ctbp2a	ENSDARG00000044062	29.39333333	60.19	0.488342	-1.0340348	0.008155	2.088580891

cth	ENSDARG00000074301	42.92333333	28.30333333	1.516547	0.6007901	0.042183	1.374859577
ctnnbl1	ENSDARG00000011958	22.47333333	18.48333333	1.21587	0.2819892	0.044239	1.354198916
CU929126.1	ENSDARG00000102443	0.926666667	2.486666667	0.372654	-1.4240907	0.037447	1.426583168
cuedc1a	ENSDARG00000068716	12.95	22.73666667	0.569565	-0.8120687	0.010062	1.997336858
CUTA	ENSDARG00000054288	8.66	4.743333333	1.82572	0.8684658	0.023868	1.622178269
cwf19l1	ENSDARG00000002128	6.573333333	9.72	0.676269	-0.5643312	0.047345	1.324726614
cx52.9	ENSDARG00000086453	0.793333333	2.83	0.28033	-1.834803	0.002823	2.549291829
cxcl12a	ENSDARG00000037116	94.37333333	53.53333333	1.762889	0.8179418	0.030244	1.519363281
cxxc1a	ENSDARG00000101996	23.92333333	4.053333333	5.902138	2.5612377	0.006967	2.156932325
cyb561d2	ENSDARG00000044114	6.156666667	3.433333333	1.793204	0.8425395	0.021198	1.673708655
cyb5r1	ENSDARG00000018966	8.94	12.74333333	0.701543	-0.511396	0.030738	1.51232174
cyhr1	ENSDARG00000061057	4.156666667	6.996666667	0.594092	-0.7512407	0.01261	1.899289444
cyt1l	ENSDARG00000036832	4119.003333	1104.243333	3.730159	1.8992372	0.005503	2.259427752
cyth3a	ENSDARG00000007807	7.72	10.96	0.70438	-0.505575	0.023133	1.635775341
dact2	ENSDARG00000056986	2.876666667	1.506666667	1.909292	0.9330378	0.00043	3.366218388
dagla	ENSDARG00000062956	5.166666667	8.353333333	0.618516	-0.6931182	0.046307	1.334350834
dalrd3	ENSDARG00000068602	14.77333333	22.96	0.643438	-0.6361273	0.034476	1.462483758
dbpb	ENSDARG00000057652	3.076666667	5.193333333	0.592426	-0.7552927	0.04849	1.314350877
dcaf15	ENSDARG00000077790	3.666666667	5.396666667	0.679432	-0.5575995	0.03032	1.518271062
dclk1a	ENSDARG00000018856	9.486666667	16.84666667	0.563118	-0.82849	0.013193	1.879643507
dcp1b	ENSDARG00000045484	2.076666667	4.716666667	0.440283	-1.183498	0.049489	1.305489124
dctn2	ENSDARG00000031388	106.59	84.30333333	1.264363	0.3384105	0.018847	1.724763697
ddb1	ENSDARG00000074431	15.20333333	30.89666667	0.49207	-1.0230635	0.016832	1.773861228
ddhd1a	ENSDARG00000086808	2.12	3.26	0.650307	-0.6208077	0.034152	1.466585156
ddx55	ENSDARG00000009864	6.503333333	5.146666667	1.263601	0.337541	0.048463	1.314587708
decr2	ENSDARG00000045257	10.97333333	7.083333333	1.549176	0.6315015	0.041392	1.383086557
dennd5b	ENSDARG00000003789	7.676666667	13.1	0.586005	-0.7710149	0.006991	2.15546363
denr	ENSDARG00000037229	52.13333333	72.96333333	0.714514	-0.4849655	0.028238	1.549162072
desi1a	ENSDARG00000033140	22.77	34.81333333	0.65406	-0.6125057	0.013495	1.869816591
desi2	ENSDARG00000004460	11.57333333	15.69	0.737625	-0.4390409	0.035739	1.446853514
dhcr7	ENSDARG00000103226	2.01	3.196666667	0.62878	-0.6693728	0.002812	2.550923452
dhrs2	ENSDARG00000021135	12.22333333	6.426666667	1.901971	0.9274952	0.008263	2.082855914
dhx37	ENSDARG00000062612	2.546666667	3.88	0.656357	-0.6074465	0.011564	1.936879337
diablob	ENSDARG00000014956	20.76	14.73	1.409369	0.495049	0.012841	1.89140114
dlgap4a	ENSDARG00000060849	9.693333333	15.24333333	0.635906	-0.6531137	0.03446	1.462686927
dmbx1a	ENSDARG00000009922	13.53333333	25.65	0.527615	-0.9224416	0.021747	1.662605333
dmxl2	ENSDARG00000091293	12.21666667	26.52	0.460659	-1.1182301	0.040682	1.390601101
dnajb12a	ENSDARG00000039363	20.81	15.93333333	1.306067	0.3852288	0.022624	1.645439091
dnajb6b	ENSDARG00000020953	14.70333333	10.94333333	1.343588	0.426091	0.008235	2.084345713
DNAJC11 (2 of 2)	ENSDARG00000102696	4.633333333	7.226666667	0.641144	-0.6412799	0.048194	1.317004116
dnajc16l	ENSDARG00000060725	12.65	7.873333333	1.606689	0.6840909	0.043896	1.357572762
dnajc18	ENSDARG00000056005	14.35333333	17.81	0.805914	-0.3113017	0.049864	1.302215027
dnajc19	ENSDARG00000044420	28.17333333	20.07666667	1.403287	0.4888105	0.019938	1.700313506
dnajc5aa	ENSDARG00000042948	9.373333333	28.24666667	0.331839	-1.5914465	0.015534	1.808720406
dnajc6	ENSDARG00000079891	10.12333333	15.27333333	0.662811	-0.5933306	0.046088	1.336414857
dnajc7	ENSDARG00000058148	25.24333333	34.94	0.722477	-0.4689772	0.030479	1.515999025
dnajc8	ENSDARG00000059373	29.12	51.36666667	0.566905	-0.8188221	0.003494	2.456692093
dnajc9	ENSDARG00000031293	52.13	33.98333333	1.533987	0.6172865	0.004482	2.348532894
dnlz	ENSDARG00000068655	7.396666667	4.103333333	1.8026	0.8500789	0.044969	1.34708411
dnph1	ENSDARG00000075730	19.42	8.106666667	2.395559	1.2603625	0.004472	2.349515407
dock10	ENSDARG00000075327	1.956666667	3.23	0.605779	-0.7231362	0.042874	1.367809666
dock11	ENSDARG00000062485	6.443333333	4.326666667	1.489214	0.5745513	0.006837	2.165134675
dpf2l	ENSDARG00000037291	61.04666667	37.16	1.642806	0.716162	0.046976	1.328128188
dph5	ENSDARG00000100242	6.776666667	10.09666667	0.671179	-0.5752314	0.004276	2.369010514

dpp3	ENSDARG00000020676	28.94	19.65	1.472774	0.5585356	0.043537	1.36113653
dpp6b	ENSDARG00000024744	9.34	13.63	0.685253	-0.5452911	0.021128	1.675150532
dpp9	ENSDARG00000052606	5.983333333	12.31333333	0.485923	-1.0412	0.034146	1.466654668
dpy30	ENSDARG00000004427	147.3633333	108.4733333	1.358521	0.4420372	0.022731	1.643373852
dpysl5b	ENSDARG00000059311	24.42333333	55.12	0.443094	-1.1743158	0.010386	1.983537326
dus4l	ENSDARG00000006567	6.706666667	3.676666667	1.824116	0.8671975	0.016693	1.777456987
DUSP18	ENSDARG00000078598	2.066666667	2.99	0.691193	-0.5328398	0.030619	1.514002563
dvl2	ENSDARG00000056184	12.22666667	8.643333333	1.414578	0.5003714	0.005901	2.229081775
dynll1	ENSDARG00000058454	380.2433333	473.6433333	0.802805	-0.3168781	0.020627	1.685564933
dyrk1ab	ENSDARG00000023814	9.553333333	12.83333333	0.744416	-0.4258198	0.023403	1.63072379
DYRK3	ENSDARG00000070600	13.55333333	19.04666667	0.711586	-0.4908908	0.015297	1.815405897
e4f1	ENSDARG00000038243	3.626666667	4.98	0.728246	-0.4575016	0.046206	1.335305081
eci2	ENSDARG00000102412	20.90666667	30.56	0.684119	-0.5476815	0.019873	1.701733931
edrf1	ENSDARG00000019834	1.383333333	2.14	0.646417	-0.629462	0.034643	1.460379261
eef1a1a	ENSDARG00000039502	18.31	24.35333333	0.751848	-0.4114875	0.027941	1.553765354
eef1a1l1	ENSDARG00000020850	6321.026667	4978.55	1.269652	0.3444333	0.032177	1.492454086
eef1g	ENSDARG00000056119	1582.993333	1242.503333	1.274035	0.3494055	0.011217	1.950129855
efnb2a	ENSDARG00000020164	15.92333333	23.25666667	0.684678	-0.546502	0.02039	1.69057242
egr1	ENSDARG00000037421	14.98666667	26.38	0.568107	-0.815765	0.028268	1.548701172
EGR4	ENSDARG00000077799	2.543333333	7.293333333	0.34872	-1.5198578	0.000156	3.808172892
EHBP1L1 (1 of 2)	ENSDARG00000057353	10.29	3.596666667	2.860982	1.5165106	0.013735	1.862165125
ehd1a	ENSDARG00000098853	15.37	8.473333333	1.813926	0.8591156	0.023761	1.62413913
eif1axb	ENSDARG00000057912	173.8633333	140.9633333	1.233394	0.3026338	0.01093	1.961383674
eif1b	ENSDARG00000012688	438.7166667	499.8866667	0.877632	-0.1883115	0.020689	1.684255041
eif3ba	ENSDARG00000059654	199.8433333	146.9533333	1.35991	0.4435114	0.003016	2.520537796
eif3k	ENSDARG00000068289	199.49	113.4833333	1.757879	0.813836	0.002154	2.666854146
eif4a2	ENSDARG00000016477	12.83666667	19.48333333	0.658854	-0.6019699	0.000188	3.726627906
eif4ba	ENSDARG00000017439	10.12333333	15.13	0.66909	-0.5797276	0.003547	2.450190054
eif4ebp1	ENSDARG00000043608	122.0533333	87.43333333	1.395959	0.4812564	0.01132	1.946160629
eif4ebp2	ENSDARG00000031819	162.2333333	119.77	1.354541	0.4378037	0.001973	2.704779295
ell	ENSDARG00000000568	7.576666667	9.333333333	0.811786	-0.3008291	0.009808	2.008433997
elmo1	ENSDARG00000098753	32.55333333	54.15	0.60117	-0.7341561	0.035036	1.455485968
elmo2	ENSDARG00000063527	10.96666667	20.25333333	0.541475	-0.8850343	0.017039	1.768551014
elovl2	ENSDARG00000045414	1.22	2.296666667	0.531205	-0.9126603	0.011803	1.928000043
elovl4b	ENSDARG00000027495	37.7	92.43	0.407876	-1.2937967	0.011764	1.929441587
elp4	ENSDARG00000008852	2.146666667	3.616666667	0.593548	-0.7525624	0.02904	1.536997935
EMC4 (2 of 2)	ENSDARG00000102733	0	94.26333333	0.010497	-6.5738491	0.044302	1.353572946
eml1	ENSDARG00000042840	6.93	15.43	0.449125	-1.1548108	0.002622	2.581446566
EML5	ENSDARG00000053517	1.566666667	3.566666667	0.439252	-1.1868781	0.002965	2.527997575
EML6	ENSDARG00000102755	4.15	7.186666667	0.577458	-0.7922114	0.007873	2.103851378
enah	ENSDARG00000032049	10.74	14.03666667	0.765139	-0.3862064	0.005028	2.298646012
ENDOD1 (7 of 12)		175.5633333	124.58	1.409242	0.4949191	0.035065	1.455126307
endog	ENSDARG00000058865	17.74333333	13.62	1.302741	0.3815504	0.0326	1.486782549
eno1b	ENSDARG00000013750	11.18	22.25333333	0.502397	-0.9931013	0.040173	1.396070904
eno2	ENSDARG00000014287	60.33	103.0866667	0.585236	-0.7729103	0.049342	1.306783814
entpd4	ENSDARG00000038422	1.363333333	3.946666667	0.345439	-1.5334963	0.038674	1.412582847
epb41l3a	ENSDARG00000002255	19.43	31.63666667	0.614161	-0.7033117	0.019553	1.70878367
epc1a	ENSDARG00000101579	14.63333333	18.45666667	0.792848	-0.3348836	0.047992	1.318835161
epc1b	ENSDARG00000060054	10.10333333	15.49333333	0.652108	-0.6168162	0.033401	1.476240655
epha8	ENSDARG00000023609	1.336666667	3.25	0.411282	-1.2818	0.03361	1.473534597
ephb6	ENSDARG00000057729	0.62	2.063333333	0.300485	-1.7346368	0.035303	1.452188041
eps8	ENSDARG00000102128	1.94	4.27	0.454333	-1.1381794	0.038781	1.411377455
ercc2	ENSDARG00000021985	4.68	6.066666667	0.771429	-0.3743955	0.029543	1.529547527
ercc3	ENSDARG00000002402	5.203333333	7.1	0.732864	-0.4483829	0.045335	1.34357087

exoc2	ENSDARG00000055610	21.59666667	16.12	1.339744	0.4219569	0.024042	1.619030641
exoc3	ENSDARG00000014582	11.58666667	16.03	0.722811	-0.4683088	0.041919	1.377584041
eya1	ENSDARG00000014259	13.32666667	10.53666667	1.26479	0.3388974	0.044255	1.354036532
F8A2		13.97	16.81	0.831053	-0.2669877	0.016102	1.793128039
fabp7a	ENSDARG00000007697	1687.13	1997.526667	0.84461	-0.2436436	0.030787	1.511634716
faimb	ENSDARG00000020814	6.406666667	25.04333333	0.255823	-1.9667808	0.028135	1.550753986
FAM126B (2 of 2)	ENSDARG00000101696	0.89	2.756666667	0.322854	-1.6310476	0.02291	1.639980839
fam131ba	ENSDARG00000070952	1.463333333	4.413333333	0.331571	-1.5926103	0.00654	2.184444081
FAM163A (1 of 2)	ENSDARG00000077470	0.383333333	2.426666667	0.157967	-2.6623046	0.02228	1.652093076
fam184a	ENSDARG00000014081	4.846666667	6.76	0.716963	-0.4800304	0.020387	1.690648062
fam196ab	ENSDARG00000104465	0.16	2.78	0.057554	-4.1189411	0.001371	2.863067107
fam207a	ENSDARG00000093061	9.14	5.51	1.658802	0.7301418	0.000438	3.358840427
fam43a	ENSDARG00000043009	16.69	26.50666667	0.629653	-0.6673713	0.042166	1.375037309
fam83d	ENSDARG00000077883	4.29	2.943333333	1.457531	0.5435267	0.050097	1.300188831
fam91a1	ENSDARG00000003320	7.943333333	13.12666667	0.60513	-0.7246842	0.000734	3.134430391
fam92a1	ENSDARG00000004436	14.50666667	10.59666667	1.368984	0.4531055	0.044268	1.353913719
farp2	ENSDARG00000016429	8.376666667	5.963333333	1.404695	0.4902573	0.002253	2.647167954
faub	ENSDARG00000043663	16.7	8.68	1.923963	0.9440812	0.015111	1.820696328
fbxl2	ENSDARG00000012135	1.74	5.54	0.314079	-1.6707987	0.002146	2.668470345
FBXO46	ENSDARG00000099814	5.086666667	7.4	0.687387	-0.5408047	0.011592	1.935855413
fbxw11a	ENSDARG00000104806	8.426666667	13.72333333	0.614039	-0.703597	0.004649	2.332642236
fbxw11b	ENSDARG00000017230	15.19	24.21	0.627427	-0.6724812	0.007077	2.150180185
fdps	ENSDARG00000040890	24.77333333	30.40666667	0.814734	-0.2955997	0.019163	1.717545491
fem1c	ENSDARG00000102147	12.97666667	16.34	0.794166	-0.3324881	0.036763	1.434588659
fen1	ENSDARG00000011404	29.45333333	23.24666667	1.266992	0.3414071	0.027773	1.556372809
fez1	ENSDARG00000023174	163.44	133.11	1.227857	0.2961422	0.036014	1.443533828
fezf2	ENSDARG00000070677	12.61666667	26.97666667	0.467688	-1.0963813	0.006389	2.194561853
fgd5a	ENSDARG00000088801	2.156666667	1.766666667	1.220755	0.2877734	0.045789	1.339241288
fgf11b	ENSDARG00000043907	0.923333333	2.473333333	0.373315	-1.4215332	0.023168	1.635115869
fgf13b	ENSDARG00000056633	20.92666667	35.55333333	0.588599	-0.7646423	0.045213	1.344738783
fgfr2	ENSDARG00000058115	37.60666667	29.59	1.270925	0.3458787	0.002942	2.531424288
FGL1	ENSDARG00000057004	2.223333333	0.776666667	2.862661	1.5173568	0.010536	1.977319356
fhl1b	ENSDARG00000056653	9.803333333	5.206666667	1.882843	0.9129123	0.02466	1.608014818
fkbp16	ENSDARG00000001976	6.09	12.03333333	0.506094	-0.9825222	0.008418	2.07481291
fkbp1ab	ENSDARG00000033567	26.59	10.38	2.561657	1.3570773	0.034618	1.460691764
flnbl	ENSDARG00000097351	26.59	14.31333333	1.857708	0.8935241	0.020035	1.698214641
flot1a	ENSDARG00000001710	55.06333333	40.03333333	1.375437	0.4598902	0.049616	1.304377736
flot2b	ENSDARG00000069774	21.99	26.87666667	0.818182	-0.2895066	0.030426	1.516758132
flrt2	ENSDARG00000079355	1.613333333	2.446666667	0.659401	-0.600773	0.024664	1.607934569
fmnl3	ENSDARG00000004372	9.956666667	6.356666667	1.566335	0.6473924	0.014174	1.848514705
FO704863.2	ENSDARG00000101523	0.976666667	2.343333333	0.416785	-1.262624	0.01825	1.738737948
fosl2	ENSDARG00000040623	4.613333333	8.873333333	0.51991	-0.9436666	0.017453	1.75812783
foxd1	ENSDARG00000029179	11.66666667	7.38	1.580849	0.6606997	0.021145	1.674795889
foxg1a	ENSDARG00000070769	7.54	15.34666667	0.491312	-1.0252889	0.00841	2.075225092
foxg1d	ENSDARG00000070053	1.343333333	3.503333333	0.383444	-1.3829109	0.028624	1.54326358
foxk2	ENSDARG00000030583	16.78333333	28.17	0.595787	-0.7471303	0.022484	1.648133639
FP103009.1	ENSDARG00000116341	10.73333333	14.21	0.755337	-0.4048084	0.006082	2.215931265
FQ311894.1	ENSDARG00000101858	5.18	9.25	0.56	-0.8365013	0.036599	1.436531839
FQ377629.1	ENSDARG00000104339	1.506666667	3.08	0.489177	-1.0315701	0.023514	1.628670581
FRG1	ENSDARG00000056504	43.67	28.57	1.528526	0.6121415	0.012119	1.916540327
FRMD1 (1 of 2)	ENSDARG00000078741	11.16666667	7.14	1.563959	0.6452026	0.031467	1.502138551
fstl1a	ENSDARG00000015559	8.786666667	3.816666667	2.302183	1.2030028	0.024443	1.611845696
fstl5	ENSDARG00000040198	15.78666667	31.03666667	0.508646	-0.975267	0.00397	2.401184685
fut8a	ENSDARG00000015449	15.63333333	24.62	0.634985	-0.6552053	0.047003	1.32787732

fut9a	ENSDARG00000016460	3.603333333	5.983333333	0.602228	-0.7316173	0.028266	1.548742814
fyna	ENSDARG00000011370	26.80666667	37.23666667	0.7199	-0.4741321	0.046762	1.330110777
fzd8a	ENSDARG00000045444	11.99333333	9.813333333	1.222147	0.2894175	0.02211	1.655412512
fzr1a	ENSDARG00000015254	37.02	28.51333333	1.29834	0.3766682	0.024783	1.605844373
fzr1b	ENSDARG00000022810	16.97666667	23.09666667	0.735027	-0.4441314	0.01666	1.778319909
GABARAPL1	ENSDARG00000040971	232.3733333	122.0866667	1.903347	0.9285389	0.002784	2.55537088
gabpb2a	ENSDARG00000007943	8.536666667	6.216666667	1.37319	0.4575316	0.023973	1.620271904
gabra6a	ENSDARG00000075058	1.073333333	4.263333333	0.251759	-1.9898837	0.007743	2.111092198
gabrb3	ENSDARG00000023771	6.756666667	18.1	0.373297	-1.4216061	0.009437	2.025185178
gabrr1	ENSDARG00000043902	0.756666667	2.853333333	0.265187	-1.9149185	0.023672	1.625765212
GABRR1 (2 of 2)	ENSDARG00000098081	2.44	5.726666667	0.426077	-1.2308145	0.018592	1.73066647
gad1b	ENSDARG00000027419	37.74	54.89333333	0.687515	-0.5405365	0.018333	1.736770713
gadd45ga	ENSDARG00000019417	33.01666667	42.96333333	0.768485	-0.3799115	0.015798	1.801392429
gapvd1	ENSDARG00000090183	2.636666667	3.896666667	0.676647	-0.5635253	0.048372	1.315403835
gas1b	ENSDARG00000067984	17.66333333	9.58	1.843772	0.8826601	0.000607	3.217137194
GATC	ENSDARG00000056855	31.78666667	21.81	1.457435	0.543432	0.02732	1.56352078
gbas		49.44666667	67.79333333	0.729374	-0.4552701	0.014762	1.830850556
gbf1	ENSDARG00000027016	4.56	6.263333333	0.728047	-0.4578968	0.001944	2.71125729
gcfc2	ENSDARG00000079020	4.61	2.983333333	1.545251	0.6278416	0.028971	1.538031982
gdap1	ENSDARG00000058601	7.9	11.56333333	0.683194	-0.5496328	0.011037	1.957150596
gdf3	ENSDARG00000037995	0.856666667	3.133333333	0.273404	-1.8708924	0.000335	3.475559534
gdpd5b	ENSDARG00000076962	7.973333333	10.99333333	0.725288	-0.463374	0.006267	2.202912208
gem	ENSDARG00000029112	3.47	7.84	0.442602	-1.175918	0.038176	1.418207205
gemin2	ENSDARG00000015638	37.34	21.62666667	1.726572	0.7879106	0.020081	1.69721082
GEN1	ENSDARG00000012325	13.36666667	7.343333333	1.820245	0.8641327	0.043032	1.366207789
gfap	ENSDARG00000025301	103.3333333	75.75	1.364136	0.4479879	0.003809	2.4191962
gfra4a	ENSDARG00000056651	0.93	2.006666667	0.463455	-1.1094984	0.03044	1.516549846
gga1	ENSDARG00000038537	12.11333333	14.16333333	0.85526	-0.2255649	0.035366	1.451412299
ggps1	ENSDARG00000023627	7.55	12.20333333	0.618683	-0.6927267	0.040006	1.397877134
gins1	ENSDARG00000007624	11.44333333	7.123333333	1.606458	0.6838829	0.044599	1.3506788
gkap1	ENSDARG00000068123	17.82333333	24.39666667	0.730564	-0.4529169	0.000478	3.32065878
glo1	ENSDARG00000068978	105.28	57.78666667	1.821874	0.8654228	0.037833	1.422132447
glod4	ENSDARG00000042509	6.276666667	4.77	1.315863	0.3960093	0.026311	1.579860132
glra4b	ENSDARG00000058103	2.673333333	5.266666667	0.507595	-0.9782504	0.043637	1.360144141
glrx3	ENSDARG00000098785	50.66333333	62.7	0.808028	-0.3075234	0.019846	1.702321402
gltscr1	ENSDARG00000061159	4.586666667	6.783333333	0.676167	-0.5645483	0.026706	1.57338989
gmppb	ENSDARG00000017658	28.28333333	24.35666667	1.161215	0.2156355	0.008842	2.053435949
gnai1	ENSDARG00000021647	32.65666667	42.3	0.772025	-0.3732801	0.02591	1.586533779
gnai2b	ENSDARG00000017294	17.01	27.19333333	0.625521	-0.6768699	0.002243	2.649183518
gnaia	ENSDARG00000044760	25.60666667	15.64666667	1.636557	0.7106641	0.006244	2.204549649
gnao1a	ENSDARG00000016676	45.37	82.57666667	0.549429	-0.8639955	0.018364	1.736042271
gnao1b	ENSDARG00000036058	56.68	119.22	0.475424	-1.0727146	0.007716	2.11259775
gnat2	ENSDARG00000042529	71.57333333	273.6	0.261598	-1.9345742	0.006246	2.204386528
gnb2	ENSDARG00000035357	14.85	30.53333333	0.486354	-1.0399222	0.016138	1.792154411
gnb2l1		2617.763333	2018.656667	1.296785	0.3749391	0.024483	1.611134856
gnb3a	ENSDARG00000004358	114.22	143.92	0.793635	-0.3334518	0.009782	2.009590174
gnb3b	ENSDARG00000002696	103.25	317.32	0.325381	-1.6197967	0.024877	1.604204727
gne	ENSDARG00000099771	10.60666667	8.62	1.230472	0.2992116	0.022492	1.64798008
gng13b	ENSDARG00000037921	94.43333333	139.75	0.67573	-0.5654802	0.004335	2.36298664
gng5	ENSDARG00000039830	158.6833333	130.5633333	1.215374	0.2814008	0.003362	2.47338583
gngt2b	ENSDARG00000103543	383.22	1558.94	0.245821	-2.0243206	0.00712	2.147525768
golga4	ENSDARG00000075331	2.886666667	4.356666667	0.662586	-0.5938202	0.019071	1.719633061
gorasp2	ENSDARG00000015126	27.77333333	40.14666667	0.691797	-0.5315799	0.007424	2.129377988
gosr1	ENSDARG00000026654	16.94	20.64666667	0.820471	-0.285475	0.005726	2.242173495

GPC6 (2 of 3)	ENSDARG00000103156	0.92	4.026666667	0.228477	-2.1298803	0.006127	2.212744791
gphnb	ENSDARG00000100851	16.99666667	29.06666667	0.584748	-0.7741138	0.028394	1.546769785
gpr107	ENSDARG00000103200	19.86333333	23.19	0.856547	-0.2233951	0.001618	2.791013938
gpr146	ENSDARG00000059610	12.78666667	19.29666667	0.662636	-0.5937114	0.040557	1.391930536
gpr63	ENSDARG00000074540	7.413333333	9.353333333	0.792587	-0.3353582	0.008507	2.070232789
gpr75	ENSDARG00000098526	7.023333333	13.86	0.506734	-0.9806994	0.012603	1.899515751
gpx1b	ENSDARG00000006207	15.5	11.16666667	1.38806	0.4730696	0.045936	1.33784645
gria1a	ENSDARG00000021352	1.913333333	3.556666667	0.537957	-0.8944375	0.041693	1.379941347
gria3b	ENSDARG00000037498	6.64	12.27666667	0.540863	-0.8866638	0.041309	1.383958459
grid1a	ENSDARG00000074583	2.283333333	3.656666667	0.62443	-0.6793876	0.042708	1.369489231
grik1a	ENSDARG00000069139	1.966666667	3.886666667	0.506003	-0.9827809	0.000794	3.100252968
grik4	ENSDARG00000026753	1.3	3.183333333	0.408377	-1.2920266	0.000881	3.055127357
grin2aa	ENSDARG00000034493	1.766666667	3.303333333	0.534813	-0.9028927	0.026704	1.573417934
grinab	ENSDARG00000052746	19.65333333	30.63666667	0.641497	-0.6404853	5.23E-05	4.28191382
grk1a	ENSDARG00000058803	1.946666667	4.616666667	0.421661	-1.2458457	0.022881	1.640523861
grk4	ENSDARG00000070448	3.736666667	6.606666667	0.56559	-0.8221707	0.026788	1.572060383
grm2a	ENSDARG00000004150	4.616666667	7.813333333	0.59087	-0.7590866	0.036991	1.431899741
grm8b	ENSDARG00000076508	2.24	5.103333333	0.438929	-1.1879411	0.025612	1.591559384
gskip	ENSDARG00000038099	53.19666667	39.02666667	1.363085	0.4468756	0.033914	1.469625193
gsna	ENSDARG00000011459	5.406666667	3.17	1.705573	0.7702566	0.043773	1.358797547
gstm	ENSDARG00000042533	101.5466667	58.78	1.727572	0.7887456	0.002419	2.616365603
gtf2h1	ENSDARG00000026701	6.606666667	9.813333333	0.673234	-0.5708207	0.023495	1.629021379
gtf2h3	ENSDARG00000067560	23.45666667	12.54666667	1.869554	0.9026939	0.006011	2.221075022
gtf2h5	ENSDARG00000056099	61.61666667	47.27	1.303505	0.3823958	0.028379	1.546998282
guca1b	ENSDARG00000013393	1.076666667	2.733333333	0.393902	-1.3440897	0.019856	1.702098495
gusb	ENSDARG00000063126	2.733333333	5.276666667	0.518004	-0.9489654	0.045377	1.343161337
h3f3a	ENSDARG00000020504	3099.346667	1833.663333	1.690248	0.7572354	0.026454	1.577515106
hacd2	ENSDARG00000014806	52.5	77.36	0.678645	-0.5592704	0.001191	2.923934352
hagh	ENSDARG00000025338	6.683333333	11.42666667	0.584889	-0.7737649	0.019543	1.708999063
hamp	ENSDARG00000102175	0.343333333	3.72	0.092294	-3.4376208	0.006597	2.180681393
HAP1	ENSDARG00000074508	3.82	8.956666667	0.426498	-1.2293893	0.001346	2.871085687
haus5	ENSDARG00000019156	1.626666667	2.34	0.695157	-0.5245899	0.000315	3.501741294
hbae1	ENSDARG00000089475	48.13	3.503333333	13.73834	3.7801363	0.000758	3.120207303
hbae1	ENSDARG00000089475	478.0833333	170.7866667	2.799301	1.4850668	0.01209	1.917579087
hbbe1.1	ENSDARG00000087390	3694.85	1298.66	2.845125	1.508492	0.001499	2.824244455
hddc3	ENSDARG00000020473	27.11333333	17.67333333	1.534138	0.6174283	0.003264	2.486314829
her15.2	ENSDARG00000054560	51.59	12.30666667	4.192037	2.0676514	0.010967	1.959898688
her4.2	ENSDARG00000056729	34.5	13.02333333	2.649091	1.4054976	0.037404	1.427081681
herc1	ENSDARG00000077901	5.096666667	6.76	0.753945	-0.4074692	0.034924	1.456881758
hexb	ENSDARG00000034368	19.52	25.92333333	0.75299	-0.4092982	0.026025	1.584601742
hhatlb	ENSDARG00000005139	16.93333333	4.196666667	4.034948	2.0125502	0.001722	2.764087712
higd1a	ENSDARG00000022303	19.98666667	32.51666667	0.614659	-0.7021415	0.000229	3.639429985
hinfp	ENSDARG00000004851	5.186666667	3.963333333	1.308663	0.3880933	0.046109	1.336215523
hip1	ENSDARG00000062972	4.943333333	7.89	0.626531	-0.6745411	0.013647	1.86497464
hip1ra	ENSDARG00000077445	1.046666667	2.393333333	0.437326	-1.1932193	0.020333	1.691806061
hk1	ENSDARG00000039452	16.69333333	35.72666667	0.467251	-1.0977293	0.008547	2.068192619
hlfa	ENSDARG00000074752	4.69	8.593333333	0.545772	-0.8736299	0.041347	1.38355532
hmbsa	ENSDARG00000008840	11.49333333	5.41	2.124461	1.0870968	0.021229	1.673068268
hmga1b	ENSDARG00000070951	24.33	50.63	0.480545	-1.0572562	0.046393	1.33354957
hmgb3b	ENSDARG00000006408	58.68	40.99333333	1.431452	0.5174796	0.039233	1.406353843
hmgcra	ENSDARG00000052734	3.193333333	7.213333333	0.442699	-1.1756029	0.029967	1.523351232
hmx2	ENSDARG00000070954	15.11333333	7.89	1.915505	0.9377247	0.017284	1.762364816
hmx4	ENSDARG00000007941	17.68	25.94333333	0.681485	-0.5532456	0.032286	1.490983041
hnrnpk	ENSDARG00000018914	33.16	54.41	0.609447	-0.7144278	0.012149	1.915447689

hnrnpul1	ENSDARG00000040564	9.43	30.55666667	0.308607	-1.6961575	0.000292	3.53523624
hnrnpul1l	ENSDARG00000079780	12.97333333	84.63333333	0.153289	-2.7056768	8.12E-05	4.090227134
homer1b	ENSDARG00000101759	14.27333333	19.37	0.736878	-0.4405017	0.030895	1.510116914
hoxa2b	ENSDARG00000023031	7.466666667	4.003333333	1.865112	0.8992626	0.002685	2.571012498
hoxa9b	ENSDARG00000056819	12.54333333	7.363333333	1.703486	0.7684899	0.025634	1.591181742
hoxb6a	ENSDARG00000010630	12.03333333	9.643333333	1.24784	0.3194325	0.04596	1.337620668
hoxb6b	ENSDARG00000026513	51.22	28.76	1.780946	0.8326436	0.029114	1.535898859
hoxb9a	ENSDARG00000056023	22.13	13.20333333	1.676092	0.7451013	0.042933	1.36720443
hoxc3a	ENSDARG00000070339	24.02	16.57	1.449608	0.5356625	0.049463	1.305716272
hpda	ENSDARG00000018351	1.143333333	3.993333333	0.286311	-1.8043474	0.003441	2.463271921
hsbp1l1	ENSDARG00000075984	4.213333333	0.66	6.383838	2.6744241	0.028249	1.548994596
hsf2	ENSDARG00000053097	19.98	24.82	0.804996	-0.3129465	0.023867	1.622211115
hsp70.2	ENSDARG00000092362	0.136666667	11.76333333	0.011618	-6.4274917	0.008779	2.056560195
hsp70.3	ENSDARG00000021924	2.146666667	3.716666667	0.577578	-0.7919111	0.00324	2.489475514
hsp90aa1.2	ENSDARG00000024746	43.42	89.27	0.48639	-1.0398157	0.023829	1.622898328
hspa4l	ENSDARG00000053544	6.34	15.49	0.409296	-1.2887824	0.029745	1.526588947
htr2cl1	ENSDARG00000018228	1.343333333	2.46	0.54607	-0.872841	0.032245	1.491535608
htt	ENSDARG00000052866	2.86	4.553333333	0.628111	-0.6709079	0.025432	1.594613219
icmt	ENSDARG00000020241	14.06333333	20.39666667	0.689492	-0.5363948	0.045728	1.339817974
id2a	ENSDARG00000055283	171.0766667	216.0566667	0.791814	-0.3367667	0.029025	1.537220554
ier5l	ENSDARG00000054906	33.73	48.63	0.693605	-0.5278143	0.028264	1.548764854
iffo1b	ENSDARG00000062108	1.743333333	6.323333333	0.275698	-1.8588368	0.03077	1.511871742
igbp1	ENSDARG00000002184	31.03	21.91333333	1.416033	0.5018547	0.031842	1.497001092
igf2b	ENSDARG00000033307	12.24666667	10.18666667	1.202225	0.2657071	0.024817	1.605248329
igf2bp3	ENSDARG00000010266	17.97	23.53666667	0.76349	-0.3893196	0.020319	1.692093398
igsf11	ENSDARG00000017217	2.526666667	4.3	0.587597	-0.7671013	0.007737	2.111453662
igsf21a	ENSDARG00000031049	4.186666667	10.91333333	0.383629	-1.3822179	0.025191	1.598752917
igsf21b	ENSDARG00000056084	5.79	11.40666667	0.507598	-0.978242	0.030405	1.517061102
igsf5a	ENSDARG00000087983	0.23	2.843333333	0.080891	-3.6278775	0.038025	1.419935818
illr1	ENSDARG00000059254	0.806666667	2.976666667	0.270997	-1.8836531	0.013197	1.879523439
impdh1a	ENSDARG00000042336	3.22	6.7	0.480597	-1.0571004	0.023268	1.633244675
inaa	ENSDARG00000011862	3.316666667	7.803333333	0.425032	-1.2343565	0.022645	1.645032341
inhbb	ENSDARG00000040777	12.83	6.766666667	1.896059	0.9230039	0.009157	2.03824363
ino80e	ENSDARG00000022939	2.716666667	1.983333333	1.369748	0.4539104	0.002375	2.624310714
ins	ENSDARG00000035350	0.703333333	3.49	0.201528	-2.3109465	0.030182	1.520253406
insm1b	ENSDARG00000053301	14.6	22.36333333	0.652854	-0.6151669	0.034984	1.456128632
insra	ENSDARG00000011948	4.906666667	6.986666667	0.70229	-0.509861	0.015234	1.817194936
ip6k2a	ENSDARG00000008310	28.86333333	44.21	0.652869	-0.6151348	0.003238	2.489719719
ip6k2b	ENSDARG00000019613	29.14333333	42.01	0.693724	-0.5275669	0.041374	1.383271585
ipmkb	ENSDARG00000029291	4.966666667	7.456666667	0.666071	-0.5862529	0.018394	1.735332807
irbp	ENSDARG00000059163	9.446666667	25.06333333	0.376912	-1.407701	0.007046	2.152031812
irf2bpl	ENSDARG00000004297	36.95	60.62666667	0.609468	-0.7143781	0.005597	2.252067587
irx4b	ENSDARG00000036051	4.82	7.32	0.65847	-0.6028105	0.022211	1.653424874
irx6a	ENSDARG00000034420	5.33	9.896666667	0.538565	-0.8928072	0.005099	2.292498714
ISCU (2 of 2)	ENSDARG00000035596	88.83666667	57.42666667	1.546958	0.6294344	0.048959	1.310165008
isl1	ENSDARG00000004023	70.59666667	94.58	0.746423	-0.4219351	0.014271	1.845530697
itfg1	ENSDARG00000103905	23.6	36.93666667	0.638932	-0.6462668	0.0478	1.320572711
itfg2	ENSDARG00000002174	3.606666667	5.256666667	0.686113	-0.5434822	0.017036	1.768625731
itm2ca	ENSDARG00000043448	44.14666667	85.49	0.516396	-0.9534512	0.008386	2.076455621
itm2cb	ENSDARG00000039650	17.82666667	23.84	0.747763	-0.4193473	0.025941	1.586010247
itpa	ENSDARG00000057529	24.80333333	14.19333333	1.747534	0.8053206	0.019231	1.715990969
itpkca	ENSDARG00000002994	11.57333333	9.663333333	1.197654	0.2602116	0.048573	1.31360863
ivns1abpa	ENSDARG00000031100	28.06333333	39.56666667	0.709267	-0.4955991	0.002965	2.527942773
JAKMIP3	ENSDARG00000100708	1.246666667	3.396666667	0.367026	-1.4460439	0.01647	1.78330398

JMJD1C (2 of 2)	ENSDARG00000100022	4.566666667	6.683333333	0.683292	-0.5494263	0.016951	1.770800055
jund	ENSDARG00000067850	4.573333333	6.736666667	0.678872	-0.5587888	0.019907	1.700995872
jupa	ENSDARG00000070787	40.03666667	23.95	1.671677	0.7412962	0.026495	1.576837013
katnal1	ENSDARG00000099156	12.57	18.39666667	0.683276	-0.5494597	0.036186	1.441458146
kcnc2	ENSDARG00000057468	1.323333333	2.613333333	0.506378	-0.9817146	0.046759	1.330132264
kcnma1a	ENSDARG00000079840	2.903333333	6.086666667	0.476999	-1.0679421	0.02978	1.526076203
kcnv2b	ENSDARG00000062906	0.97	3.103333333	0.312567	-1.677762	0.005738	2.241206039
kctd12.2	ENSDARG00000053542	10.68666667	16.78333333	0.636743	-0.6512174	0.044598	1.350683404
kdelc2	ENSDARG00000021408	8.026666667	5.463333333	1.469189	0.5550195	0.029664	1.527767033
kdm5ba	ENSDARG00000057093	14.95	19.81	0.754669	-0.4060834	0.001373	2.862465425
kdm6al	ENSDARG00000059794	4.253333333	8.13	0.523165	-0.9346614	0.015175	1.818870372
kdm7ab	ENSDARG00000018559	8.716666667	10.80666667	0.806601	-0.3100731	0.026829	1.571398201
khk	ENSDARG00000029874	3.123333333	4.15	0.75261	-0.4100248	0.024789	1.605737089
kidins220b	ENSDARG00000017338	34.95	59.26	0.589774	-0.7617662	0.02353	1.628379486
kif14	ENSDARG00000062187	3.42	1.976666667	1.730185	0.7909267	0.024538	1.610162968
kif21a	ENSDARG00000061131	1.463333333	2.78	0.526379	-0.9258264	0.042424	1.372390779
kif3a	ENSDARG00000087538	19.88333333	28.24333333	0.704001	-0.5063507	0.033699	1.472383574
kifap3b	ENSDARG00000006862	17.60666667	23.81333333	0.739362	-0.4356478	0.017182	1.764928503
kifc3	ENSDARG00000054978	16.71333333	25.14333333	0.664722	-0.5891764	0.027046	1.567901451
klc2	ENSDARG00000099740	27.83	45.4	0.612996	-0.7060514	0.02347	1.629492248
klhl17	ENSDARG00000037324	5.8	8.096666667	0.716344	-0.4812752	0.001276	2.894062567
KLHL25	ENSDARG00000102418	1.356666667	2.943333333	0.460929	-1.1173846	0.00861	2.065015456
KLHL29 (2 of 2)	ENSDARG00000087688	3.31	7.906666667	0.418634	-1.2562384	0.013244	1.877989816
krt96	ENSDARG00000095147	11.44666667	3.806666667	3.007005	1.5883274	0.030446	1.516463979
kxd1	ENSDARG00000051934	9.036666667	5.883333333	1.535977	0.6191569	0.045746	1.339643358
l2hgdh	ENSDARG00000060500	2.426666667	3.32	0.730924	-0.4522073	0.023035	1.637609736
l3mbtl2	ENSDARG00000056079	4.48	6.696666667	0.66899	-0.5799444	0.03568	1.447577257
lactbl1a	ENSDARG00000089063	3.373333333	10.46	0.322498	-1.6326361	0.009724	2.012149731
lamb2l	ENSDARG00000033950	6.373333333	5.31	1.200251	0.2633363	0.014404	1.841508505
lancl2	ENSDARG00000045465	6.73	11.25	0.598222	-0.7412466	0.049735	1.303337292
larp4b	ENSDARG00000062146	2.753333333	4.07	0.676495	-0.5638495	0.024199	1.616197472
lbh	ENSDARG00000087377	1.016666667	2.21	0.46003	-1.1201996	0.032893	1.482900091
lbr	ENSDARG00000014013	68.86666667	37.77666667	1.822995	0.8663104	0.020054	1.697788573
lcn15	ENSDARG00000067851	1.246666667	2.876666667	0.433372	-1.2063223	0.037145	1.430096598
ldhbb	ENSDARG00000071076	6.893333333	16.09333333	0.428335	-1.2231895	0.001644	2.78406376
leng8	ENSDARG00000076805	3.863333333	5.6	0.689881	-0.5355807	0.01919	1.716917103
LEPROTL1	ENSDARG00000104871	42.71666667	63.12	0.676753	-0.5632981	0.035891	1.445020483
letm2	ENSDARG00000077515	16.02333333	21.67333333	0.739311	-0.4357468	0.002974	2.526628367
lgals3	ENSDARG00000077850	31.49	19.36666667	1.62599	0.7013181	0.04906	1.309270527
lgals3bpb	ENSDARG00000040528	3.79	1.13	3.353982	1.7458751	0.009976	2.001033258
lgr4	ENSDARG00000060542	3.59	5.12	0.701172	-0.51216	0.011006	1.958374778
lhpp	ENSDARG00000060196	0.35	2.413333333	0.145028	-2.7856004	0.024417	1.612301004
lin7a	ENSDARG00000013414	38.15333333	64.94333333	0.587487	-0.7673723	0.038174	1.418227098
lingo1b	ENSDARG00000035899	9.536666667	11.62	0.820711	-0.2850531	0.001858	2.730982149
lingo2a	ENSDARG00000074535	5.95	10.45	0.569378	-0.8125414	0.021571	1.66613871
lipf	ENSDARG00000018529	17.06333333	10.40333333	1.640179	0.7138536	0.036935	1.432566924
lman2	ENSDARG00000061854	54.74333333	45.22333333	1.210511	0.2756159	0.002939	2.531840148
lmod1a	ENSDARG00000056111	2.516666667	1.076666667	2.337461	1.2249425	0.027479	1.561002969
lmx1bb	ENSDARG00000068365	8.306666667	6.366666667	1.304712	0.3837314	0.04389	1.357636865
lnx2a	ENSDARG00000029177	9.436666667	16.22	0.581792	-0.7814246	0.019178	1.717205891
lpcat1	ENSDARG00000011506	14.67666667	10.3	1.424919	0.51088	0.005806	2.236121411
lpgat1	ENSDARG00000013542	6.45	9.63	0.669782	-0.5782366	0.001764	2.753591167
lppr5b	ENSDARG00000101348	0.536666667	2.4	0.223611	-2.1609362	0.044771	1.348999965
lrit1b	ENSDARG00000099406	3.823333333	11.71666667	0.326316	-1.6156593	0.042695	1.369620567

lrit3a	ENSDARG00000055463	1.513333333	4.246666667	0.356358	-1.4886011	0.024505	1.610752639
LRR1	ENSDARG00000104448	6.8	2.526666667	2.691293	1.4282994	0.032051	1.494151907
lrrc73	ENSDARG00000063411	5.323333333	8.586666667	0.619953	-0.6897683	0.037404	1.427086866
LRRN2	ENSDARG00000105302	10.33666667	15.02666667	0.687888	-0.539754	0.037632	1.424445907
lrrtm1	ENSDARG00000052713	17.90333333	30.06	0.595587	-0.7476168	0.028481	1.545439109
lrtm2b	ENSDARG00000045811	1.633333333	4.583333333	0.356364	-1.488578	0.032184	1.492359299
LSM3	ENSDARG00000089663	95.14	71.57666667	1.329204	0.4105627	0.006092	2.21523633
lsm6	ENSDARG00000036995	365.5266667	213.8033333	1.70964	0.7736923	0.011836	1.926809962
luc7l	ENSDARG00000055903	36	53.11666667	0.677753	-0.5611677	0.002476	2.606334858
luc7l3	ENSDARG00000014366	16.78666667	25.54	0.65727	-0.6054427	0.016298	1.787857448
lyrm5b	ENSDARG00000045827	2.386666667	0.306666667	7.782609	2.9602538	0.007621	2.117990337
m6pr	ENSDARG00000018432	20.07333333	14.41	1.393014	0.4782099	0.000112	3.950279838
magi1a	ENSDARG00000070145	13.87333333	19.07333333	0.727368	-0.4592425	0.042911	1.367428347
man1b1a	ENSDARG00000073792	2.62	5.783333333	0.453026	-1.1423344	0.030009	1.522755099
man2b1	ENSDARG00000001897	6.563333333	3.72	1.764337	0.8191261	0.010513	1.978270833
MAN2B2	ENSDARG00000079463	1.203333333	2.196666667	0.5478	-0.8682796	0.019788	1.703593955
mansc1	ENSDARG00000104839	6.99	16.51333333	0.423294	-1.240267	0.021806	1.661426965
map1sb	ENSDARG00000060326	2.35	3.74	0.628342	-0.6703775	0.009161	2.038067734
map2k5	ENSDARG00000011851	2.353333333	4.32	0.544753	-0.8763256	0.039145	1.407327195
map6b	ENSDARG00000074073	2.363333333	3.826666667	0.617596	-0.6952651	0.032033	1.494405837
MAP7 (2 of 2)	ENSDARG00000104435	1.46	3.193333333	0.457203	-1.1290948	0.035515	1.449582245
mapk8ip3	ENSDARG00000062531	8.793333333	17.89333333	0.491431	-1.0249401	0.039935	1.398641913
mapre3b	ENSDARG00000102878	11.42666667	18.6	0.614337	-0.702898	0.024679	1.607665742
march5l	ENSDARG00000028559	12.96333333	16.41666667	0.789645	-0.3407245	0.040568	1.391812383
mark3b	ENSDARG00000026630	9.48	14.39666667	0.658486	-0.6027759	0.002099	2.678051012
masp1	ENSDARG00000068726	4.093333333	1.583333333	2.585263	1.3703111	0.037736	1.42324435
max	ENSDARG00000024844	77.29333333	123.0966667	0.627908	-0.6713758	0.002102	2.677285379
mbpb	ENSDARG00000089413	27.55333333	22.51333333	1.223867	0.2914472	0.026313	1.579832476
mcat	ENSDARG00000016613	3.24	1.25	2.592	1.3740657	0.001867	2.728844416
mcm3	ENSDARG00000024204	20.86333333	13.57	1.53746	0.620549	0.00666	2.176532661
mcm4	ENSDARG00000040041	38.22666667	28.15	1.357963	0.4414445	0.025112	1.600115888
mcm5	ENSDARG00000019507	38.99	21.52333333	1.811522	0.8572026	0.000612	3.212998105
mcoln1a	ENSDARG00000002285	3.156666667	4.556666667	0.692758	-0.5295769	0.035316	1.452028372
mdkb	ENSDARG00000020708	291.54	473.23	0.616064	-0.6988477	0.016268	1.788659803
med18	ENSDARG00000041237	12.70666667	6.76	1.879684	0.9104905	0.01771	1.751776865
mef2aa	ENSDARG00000031756	4.69	6.406666667	0.73205	-0.449986	0.014209	1.847435975
meox1	ENSDARG00000007891	5.88	2.796666667	2.102503	1.0721078	0.020785	1.682244746
mettl2a	ENSDARG00000008105	23.47666667	15.79333333	1.486492	0.5719119	0.018705	1.728048485
mettl5	ENSDARG00000068893	18.12666667	9.513333333	1.905396	0.9300908	0.018935	1.722740224
mettl6	ENSDARG00000070085	5.66	1.953333333	2.897611	1.5348639	0.043646	1.36005108
mettl7a	ENSDARG00000056726	2.383333333	4.436666667	0.53719	-0.8964954	0.042259	1.374083128
mfge8b	ENSDARG00000045803	2.756666667	4.293333333	0.642081	-0.6391734	0.020374	1.69091583
mfn2	ENSDARG00000079504	9.086666667	13.13	0.692054	-0.5310439	0.045139	1.3454462
mfsd5	ENSDARG00000015997	9.36	6.873333333	1.361785	0.4454986	0.042666	1.369916366
mgme1	ENSDARG00000016656	7.88	4.28	1.841121	0.8805848	0.035715	1.447153364
mgrn1b	ENSDARG00000004796	15.39	18.98	0.810854	-0.3024868	0.030194	1.520081264
mgst1.1	ENSDARG00000032618	10.10666667	13.71	0.737175	-0.4399213	0.048811	1.31148217
mibp	ENSDARG00000101362	40.38333333	28.84666667	1.399931	0.4853554	0.031471	1.502085512
mier1a	ENSDARG00000041072	16.35333333	9.613333333	1.70111	0.7664761	0.047095	1.327022144
mllt1b	ENSDARG00000031709	2.563333333	4.32	0.593364	-0.7530102	0.026215	1.581445762
mmab	ENSDARG00000068344	14.6	6.44	2.267081	1.1808358	0.021472	1.668121716
MMP23B	ENSDARG00000043079	2.293333333	3.873333333	0.592083	-0.7561296	0.007753	2.11053178
mob1bb	ENSDARG00000012953	5.55	10.07666667	0.550777	-0.8604588	0.034839	1.457932423
mpc2	ENSDARG00000024478	141.62	108.05	1.310689	0.3903259	0.03261	1.486647585

mpdu1a	ENSDARG00000035562	6	3.7	1.621622	0.6974372	0.028013	1.552643797
mpeg1.1	ENSDARG00000055290	2.073333333	0.826666667	2.508065	1.3265745	0.040171	1.396087371
mpi	ENSDARG00000030786	1.763333333	4.016666667	0.439004	-1.1876935	0.009721	2.012294284
mpp2b	ENSDARG00000010957	11.14	16.59	0.671489	-0.5745647	0.001284	2.891549467
MPP4 (2 of 2)	ENSDARG00000076872	1.036666667	23.10333333	0.044871	-4.478077	0.003922	2.406530943
mpv17l2	ENSDARG00000056367	3.503333333	6.286666667	0.557264	-0.843567	0.026549	1.575953338
mrpl1	ENSDARG00000054606	29.42666667	15.25666667	1.928774	0.9476843	0.007648	2.116435281
mrpl10	ENSDARG00000045091	23.07333333	25.54333333	0.903302	-0.1467204	0.034378	1.463718072
mrpl3	ENSDARG00000103318	55.41	35.39666667	1.565402	0.6465329	0.041448	1.382496413
mrpl44	ENSDARG00000094277	23.40666667	16.93333333	1.382283	0.4670535	0.04044	1.393192551
mrps2	ENSDARG00000103198	31.87666667	23.74666667	1.342364	0.4247758	0.033045	1.480893385
mrps24	ENSDARG00000100116	88.61333333	61.85	1.432714	0.5187502	0.043806	1.358469719
msrb1a	ENSDARG00000025436	1.026666667	3.11	0.330118	-1.5989467	0.040738	1.389995626
mt-atp6	ENSDARG00000063911	8376.62	12481.31667	0.671133	-0.57533	0.032769	1.484534778
mt-atp8	ENSDARG00000063910	2437.64	3991.076667	0.610773	-0.7112929	0.014154	1.849127499
mtch2	ENSDARG00000019732	43.89	65.14333333	0.673745	-0.5697253	0.007423	2.129446225
mtfr1	ENSDARG00000045304	4.99	2.476666667	2.014805	1.0106401	0.030912	1.509876432
mtfr2	ENSDARG00000060394	6.32	3.7	1.708108	0.7723993	0.015248	1.816790327
mthfd2	ENSDARG00000098646	11.19333333	18.16666667	0.616147	-0.698654	0.041262	1.384453998
mtmr6	ENSDARG00000016794	5.446666667	8.686666667	0.627015	-0.6734291	0.017919	1.746685401
mtmr8	ENSDARG00000008592	2.82	1.65	1.709091	0.7732291	0.042622	1.370361441
mt-nd1	ENSDARG00000063895	2213.38	3155.88	0.701351	-0.5117912	0.015284	1.815766243
mt-nd2	ENSDARG00000063899	1442.823333	2287.34	0.630787	-0.6647762	0.016612	1.779589104
mtus1b	ENSDARG00000034105	8.693333333	15.08666667	0.576226	-0.7952927	0.027662	1.558124094
mtx1b	ENSDARG00000036109	2.623333333	5.333333333	0.491875	-1.0236364	0.036557	1.437032545
mul1a	ENSDARG00000021398	3.45	2.523333333	1.367239	0.4512656	0.022824	1.641612551
mvdb		13.08	17.57333333	0.74431	-0.4260253	0.036436	1.438464898
mybpc2b	ENSDARG00000021265	12.72	6.506666667	1.954918	0.9671081	0.049631	1.304247577
mycbp2	ENSDARG00000001220	10.04	14.69666667	0.683148	-0.5497297	0.031043	1.508038591
myef2	ENSDARG00000059398	35.35333333	57.06666667	0.619509	-0.690802	0.029281	1.533419173
myhz1.1	ENSDARG00000067990	5013.203333	3515.986667	1.425831	0.5118032	0.014187	1.848102123
mylipa	ENSDARG00000008859	3.8	5.083333333	0.747541	-0.4197754	0.012016	1.920230909
myo3a	ENSDARG00000010186	1.9	4.593333333	0.413643	-1.2735421	0.001136	2.944758719
myo5aa	ENSDARG00000061635	3.356666667	5.37	0.625078	-0.6778928	0.003226	2.491355005
n4bp2l2	ENSDARG00000103507	14.17666667	11.02	1.286449	0.3633941	0.034145	1.466669612
naa25	ENSDARG00000075446	10.64333333	17.18666667	0.619279	-0.6913397	0.010705	1.970421339
nbeaa	ENSDARG00000070080	4.373333333	7.206666667	0.606846	-0.7205988	0.047664	1.321812258
NBR1 (2 of 2)	ENSDARG00000078772	6.46	9.24	0.699134	-0.5163587	0.007859	2.104616098
ncaph2	ENSDARG00000033757	13.84333333	8.786666667	1.575493	0.6558035	0.041814	1.378681682
nckap1	ENSDARG00000060853	26.75	34.72333333	0.770375	-0.3763666	0.032719	1.485194983
nckipsd	ENSDARG00000079475	3.836666667	6.183333333	0.620485	-0.6885314	0.036689	1.435460535
ncoa4	ENSDARG00000021439	34.07666667	23.38333333	1.457306	0.5433036	0.001521	2.817876801
ndnl2	ENSDARG00000058212	17.81666667	10.24666667	1.738777	0.7980728	0.008186	2.086953134
ndrg1a	ENSDARG00000032849	25.08666667	57.56333333	0.43581	-1.1982293	0.012655	1.897721806
ndrg3b	ENSDARG00000010052	25.06	39.88666667	0.62828	-0.6705201	0.005066	2.295322611
ndrg4	ENSDARG00000103937	57.04333333	122.3666667	0.466167	-1.1010804	0.002008	2.697206235
NDUFAF3	ENSDARG00000112931	72.62	58.63	1.238615	0.3087279	0.026928	1.569788763
necab2	ENSDARG00000056745	28.78	50.53	0.569563	-0.8120736	0.029948	1.52362494
nefma	ENSDARG00000021351	13.87666667	24.85	0.558417	-0.8405848	0.024101	1.617973886
nelfb	ENSDARG00000035505	6.836666667	10.10333333	0.676674	-0.5634664	0.01972	1.70508789
nelfcd	ENSDARG00000021097	15.19	8.42	1.804038	0.8512297	0.000553	3.25762322
nfasca	ENSDARG00000061099	6.166666667	8.466666667	0.728346	-0.4573032	0.029021	1.537293284
nfkbib	ENSDARG00000030087	56.57666667	41.68	1.357406	0.4408519	0.029739	1.526679502
nfybb	ENSDARG00000045611	16.68666667	14.02333333	1.189922	0.2508665	0.004121	2.385016838

nhsb	ENSDARG00000079977	6.213333333	10.00333333	0.621126	-0.6870415	0.026163	1.582320294
nhsl1a	ENSDARG00000054537	4.453333333	7.65	0.582135	-0.7805741	0.000426	3.370995714
nipa2	ENSDARG00000055912	14.72666667	10.96	1.343674	0.4261831	0.012477	1.903905464
nisch	ENSDARG00000043077	4.26	6.193333333	0.687836	-0.5398627	0.037236	1.429041231
nkx2.4a	ENSDARG00000075107	2.64	3.683333333	0.716742	-0.480474	0.034263	1.465172266
nln	ENSDARG00000019233	4.173333333	2.58	1.617571	0.6938291	0.023567	1.627704236
npas4a	ENSDARG00000055752	1.46	4.886666667	0.298772	-1.7428823	0.001243	2.905434707
NPBWR2 (1 of 2)	ENSDARG00000033393	1.736666667	3.793333333	0.457821	-1.1271453	0.027825	1.555560783
npepps	ENSDARG00000044943	16.59	24.55	0.675764	-0.5654091	0.026619	1.574800696
npm3	ENSDARG00000103594	28.93666667	17.39333333	1.663664	0.7343643	0.020317	1.692142549
nptnb	ENSDARG00000043864	28.53	58.07666667	0.491247	-1.0254789	0.003779	2.422650093
nptx1l	ENSDARG00000074671	11.44333333	16.84	0.679533	-0.5573848	0.023448	1.629892079
nrg2b	ENSDARG00000086585	3.106666667	5.96	0.521253	-0.9399449	0.027737	1.556942644
nrgna	ENSDARG00000039626	32.36	70.92	0.456289	-1.1319809	0.031136	1.506735358
nrip2	ENSDARG00000079985	0.8	3.44	0.232558	-2.1043367	0.026193	1.581813732
nrm	ENSDARG00000063690	4.093333333	2.13	1.921753	0.9424227	0.040528	1.392239876
nrxn3b	ENSDARG00000062693	0.243333333	2.78	0.08753	-3.514079	0.037635	1.424410975
nsfa	ENSDARG00000007654	50.95666667	81.17	0.627777	-0.6716757	0.012611	1.899241322
nsmce2	ENSDARG00000076379	7.34	4.553333333	1.612006	0.688857	0.028119	1.551001898
nt5c1ba	ENSDARG00000018153	3.833333333	7.87	0.487082	-1.0377642	0.035166	1.453877412
nt5dc2	ENSDARG00000059646	24.79666667	21.78666667	1.138158	0.1867007	0.028234	1.549232535
ntmt1	ENSDARG00000022399	20.79	12.68	1.63959	0.713335	0.012467	1.904224028
ntng1a	ENSDARG00000014973	2.613333333	7.48	0.349376	-1.5171471	0.005647	2.248200746
ntrk3a	ENSDARG00000077228	1.636666667	3.213333333	0.509336	-0.9733101	0.045612	1.340921379
nucb2b	ENSDARG00000036291	11.63	6.906666667	1.68388	0.7517896	0.004771	2.321434016
nudt15	ENSDARG00000022461	6.213333333	3.06	2.030501	1.0218358	0.022749	1.643046303
nudt22	ENSDARG00000071671	5.72	3.456666667	1.654773	0.7266337	0.018263	1.738431143
nudt5	ENSDARG00000078073	36.07	16.45333333	2.192261	1.1324195	0.004374	2.359133743
nup107	ENSDARG00000016010	19.42333333	13.20666667	1.470722	0.5565244	0.026363	1.579006707
nxf1	ENSDARG00000055076	16.22666667	24.98	0.649586	-0.6224068	0.012664	1.897435527
nyx	ENSDARG00000061791	1.026666667	4.45	0.230712	-2.1158375	0.002869	2.542206033
ogfod2	ENSDARG00000056211	2.593333333	1.396666667	1.856802	0.8928199	0.005117	2.291026124
oip5	ENSDARG00000093597	13.34666667	6.796666667	1.963708	0.9735802	0.045845	1.338710435
ola1	ENSDARG00000044565	124.0166667	105.7266667	1.172993	0.2301947	0.038182	1.418136305
olah	ENSDARG00000058502	7.383333333	1.936666667	3.812392	1.9306966	0.000372	3.429526025
olfm1a	ENSDARG00000018270	14.05	21.33666667	0.658491	-0.6027647	0.036766	1.434558985
olfm3a	ENSDARG00000071493	3.913333333	8.253333333	0.474152	-1.0765789	0.022356	1.650611916
opn1mw1	ENSDARG00000097008	148.57	493.6566667	0.300958	-1.7323652	0.025507	1.59333741
opn1mw2	ENSDARG00000044280	4.44	22.16666667	0.200301	-2.3197603	0.000271	3.567338452
opn1sw1	ENSDARG00000045677	242.7366667	939.0866667	0.258482	-1.9518663	0.0088	2.055522369
opn1sw2	ENSDARG00000017274	109.8266667	364.18	0.301572	-1.7294233	0.016223	1.789871223
opn4.1	ENSDARG00000007553	2.713333333	7.9	0.34346	-1.5417864	0.018551	1.731633792
orc4	ENSDARG00000101161	13.47666667	9.57	1.40822	0.4938729	0.025926	1.586262353
orc6	ENSDARG00000075682	8.666666667	5.786666667	1.497696	0.5827447	0.024388	1.612819729
osbp2	ENSDARG00000053487	4.403333333	6.463333333	0.681279	-0.5536823	0.01658	1.780410557
OSBPL10 (2 of 2)	ENSDARG00000099747	1.68	4.006666667	0.419301	-1.2539413	0.000961	3.017349267
OSCP1 (1 of 2)	ENSDARG00000101888	5.996666667	9.47	0.633228	-0.6592036	0.039239	1.406282102
otud4	ENSDARG00000104790	100.8233333	427.7433333	0.23571	-2.0849158	0.001373	2.862359972
otud5a	ENSDARG00000059006	15.97666667	19.57	0.816386	-0.2926773	0.045824	1.338911029
oxr1b	ENSDARG00000063310	5.52	9.09	0.607261	-0.719612	0.000974	3.011575758
oxsr1b	ENSDARG00000027500	8.103333333	4.046666667	2.002471	1.0017815	0.020557	1.687034262
p2rx3b	ENSDARG00000029718	4.326666667	2.19	1.975647	0.9823251	0.022103	1.655551069
p3h1	ENSDARG00000071212	13.57	9.63	1.409138	0.494813	0.004636	2.333836593
p4ha1b	ENSDARG00000071082	12.16	6.193333333	1.963402	0.9733552	0.006706	2.173526991

pabpn1	ENSDARG00000045373	91.11666667	130.62	0.697571	-0.5195889	0.003108	2.507562438
pacsin1b	ENSDARG00000042128	13.92	22.60333333	0.615838	-0.6993763	0.031497	1.501730712
pafah1b1b	ENSDARG00000026595	26.19	41.57666667	0.629921	-0.666758	0.033686	1.472555585
pak1	ENSDARG00000103959	21.61333333	31.99	0.675628	-0.5656994	0.021792	1.661702399
pak1ip1	ENSDARG00000044488	19.03333333	14.37333333	1.324212	0.4051336	0.029763	1.526318033
pak7	ENSDARG00000030154	9.293333333	12.07666667	0.769528	-0.3779542	0.038276	1.41706979
parp6b	ENSDARG00000070473	11.4	14.85	0.767677	-0.3814291	0.01833	1.736838211
PAXBP1	ENSDARG00000062361	5.736666667	6.95	0.82542	-0.2768003	0.01653	1.781724108
pbxip1a	ENSDARG00000071015	15.85333333	13.47333333	1.176645	0.2346794	0.040723	1.390156872
pcbp4	ENSDARG00000024276	11.77333333	20.58666667	0.571891	-0.8061874	0.009985	2.00065467
pcdh18a	ENSDARG00000089805	6.673333333	8.923333333	0.747852	-0.4191752	0.032834	1.483681868
pcdh7a	ENSDARG00000078898	8.58	11.66666667	0.735429	-0.4433429	0.014907	1.826600263
pcdh7b	ENSDARG00000060610	3.763333333	8.58	0.438617	-1.1889666	0.016753	1.775912151
pcm1	ENSDARG00000062198	7.246666667	9.223333333	0.785688	-0.3479707	0.015568	1.807774253
pcnx	ENSDARG00000011819	3.986666667	5.68	0.701878	-0.5107079	0.049512	1.305290873
pcp4a	ENSDARG00000053130	100.1033333	176.2366667	0.568005	-0.8160241	0.029347	1.532439649
pcyt1ab	ENSDARG00000004492	5.923333333	8.55	0.692788	-0.5295151	0.043142	1.365098963
pdcb	ENSDARG00000017634	17.41	66.96666667	0.25998	-1.943527	0.001473	2.831920368
pdcd2l	ENSDARG00000002754	1.45	2.733333333	0.530488	-0.9146085	0.020483	1.688597884
pddc1	ENSDARG00000053609	42.78333333	26.52666667	1.612842	0.6896055	0.001536	2.813665068
pde4ba	ENSDARG00000032868	28.88666667	37.1	0.778616	-0.3610155	0.009066	2.042590922
pde6b	ENSDARG00000011671	1.09	2.093333333	0.520701	-0.9414739	0.033413	1.476078783
pde6c	ENSDARG00000100397	11.55333333	47.81333333	0.241634	-2.0491038	0.00374	2.427113082
pde6h	ENSDARG00000102558	53.98	218.6766667	0.246848	-2.0183024	0.040688	1.390536977
pdgfaa	ENSDARG00000055505	7.373333333	10.46333333	0.704683	-0.5049536	0.025765	1.588975682
pdha1a	ENSDARG00000012387	76.52333333	99.27333333	0.770835	-0.3755065	0.028873	1.539508557
pdhb	ENSDARG00000021346	109.79	123.8966667	0.886142	-0.1743907	0.034981	1.456170812
pdhx	ENSDARG00000051756	17.70666667	26.82333333	0.660122	-0.5991959	0.029619	1.528436679
pdia4	ENSDARG00000018491	47.01	33.82333333	1.389869	0.4749488	0.023696	1.625319687
pdxka	ENSDARG00000088959	3.726666667	7.253333333	0.513787	-0.9607584	0.002489	2.604002624
pef1	ENSDARG00000023989	9.106666667	6.356666667	1.432617	0.5186526	0.018264	1.738394505
PELI3	ENSDARG00000087452	8.31	13.40666667	0.619841	-0.6900302	0.025882	1.586995701
pelo	ENSDARG00000055477	34.41666667	24.15666667	1.424727	0.510686	0.003303	2.481117367
per2	ENSDARG00000034503	4.763333333	10.83666667	0.439557	-1.1858776	0.033983	1.46873239
pes	ENSDARG00000018902	42.46333333	30.80333333	1.37853	0.4631311	0.018374	1.735801418
pfdn4	ENSDARG00000061228	87.29666667	64.27333333	1.35821	0.4417063	0.017796	1.749669886
pfkpa	ENSDARG00000028000	14.41333333	25.90333333	0.556428	-0.8457337	0.046214	1.335224679
pgam1b	ENSDARG00000014068	44.41666667	85.74333333	0.518019	-0.9489234	0.009277	2.032609455
pgls	ENSDARG00000021233	4.093333333	12.56	0.325902	-1.6174884	0.017693	1.752186547
pgm3	ENSDARG00000024654	5.67	3.203333333	1.770031	0.8237748	0.025392	1.595310051
PHC3	ENSDARG00000079167	4.5	7.67	0.586701	-0.7693016	0.005198	2.284187844
phf5a	ENSDARG00000045155	154.5766667	91.18333333	1.695229	0.7614805	0.025295	1.596966267
phtf2	ENSDARG00000102123	13.01	20.20666667	0.643847	-0.6352104	0.040117	1.396675233
phyh	ENSDARG00000086740	9.646666667	13.57333333	0.710707	-0.4926726	0.028686	1.542333241
PHYHIP	ENSDARG00000078176	3.09	6.44	0.479814	-1.0594539	0.009421	2.025884436
pi4k2b	ENSDARG00000013881	6.256666667	8.14	0.768632	-0.3796346	0.047489	1.323410134
picalma	ENSDARG00000012866	8.516666667	12.20666667	0.697706	-0.5193085	0.03209	1.493632856
pif1	ENSDARG00000020289	9.233333333	4.27	2.162373	1.1126155	0.037481	1.426184223
pigl	ENSDARG00000067752	6.506666667	4.47	1.45563	0.5416438	0.017938	1.746217725
pign	ENSDARG00000077532	0.913333333	2.116666667	0.431496	-1.2125807	0.02161	1.665341053
pih1d1	ENSDARG00000037955	7.213333333	3.73	1.93387	0.9514905	0.024131	1.617429137
pik3r1	ENSDARG00000038524	22.37333333	30.23666667	0.73994	-0.4345189	0.008126	2.09010093
pik3r4	ENSDARG00000060469	4.893333333	7.016666667	0.697387	-0.5199683	0.015215	1.817729946
pikfyve	ENSDARG00000056112	5.106666667	7.056666667	0.723666	-0.466605	0.003313	2.479832816

pim3	ENSDARG00000055129	26.98666667	19.23666667	1.402876	0.488388	0.030791	1.511580969
pin4	ENSDARG00000004527	79.39666667	49.95	1.589523	0.6685938	0.001981	2.703101713
pkdccb	ENSDARG00000038235	3.306666667	4.556666667	0.725677	-0.4626012	0.022554	1.646769039
PKIB (2 of 2)	ENSDARG00000095152	3.993333333	8.823333333	0.452588	-1.1437303	0.001162	2.934712096
PLCXD2	ENSDARG00000058200	0.866666667	4.673333333	0.185449	-2.4309028	0.037815	1.422338673
plcxd3	ENSDARG00000054794	10.97333333	14.77333333	0.74278	-0.4289935	0.014429	1.840762644
PLEKHB1	ENSDARG00000063519	1.94	9.27	0.209277	-2.2565127	0.00188	2.725908468
plekho1a	ENSDARG00000061191	9.263333333	13.54666667	0.683809	-0.5483346	7.63E-05	4.117347523
plk2a	ENSDARG00000090752.	5.023333333	6.766666667	0.742365	-0.4298003	0.035197	1.453497325
plrg1	ENSDARG00000037283	31.63333333	20.46	1.546106	0.6286394	0.026999	1.568655296
podxl2		5.936666667	8.14	0.72932	-0.4553757	0.028719	1.54183046
polb	ENSDARG00000003749	73.98333333	48.85333333	1.514397	0.5987433	0.007674	2.114955116
pole3	ENSDARG00000008551	17.77666667	29.67666667	0.599012	-0.7393442	0.035573	1.448884817
polg2	ENSDARG00000074933	5.953333333	3.496666667	1.702574	0.7677174	0.022694	1.644096438
polr2gl	ENSDARG00000056127	87.94	57.77333333	1.522156	0.6061158	0.007703	2.113355195
polr2i	ENSDARG00000040443	68.76	40.65333333	1.691374	0.7581959	0.015241	1.816995164
pom121	ENSDARG00000055113	2.296666667	5.423333333	0.423479	-1.2396384	0.005704	2.243787754
pomk	ENSDARG00000003208	5.01	3.623333333	1.382705	0.4674931	0.041253	1.384546823
pomt1	ENSDARG00000067670	2.11	4.4	0.479545	-1.0602605	0.015134	1.820059181
pop5	ENSDARG00000053452	15.33333333	10.73333333	1.428571	0.5145732	0.031088	1.507413649
pou3f3a	ENSDARG00000042032	11.7	17.85666667	0.655217	-0.6099543	0.020725	1.683504112
pou4f1	ENSDARG00000005559	13.13	19.15	0.68564	-0.5444775	0.04221	1.374580452
ppa1a	ENSDARG00000099933	1.34	8.126666667	0.164889	-2.6004307	0.024492	1.610982661
ppa2	ENSDARG00000009685	8.633333333	6.616666667	1.304786	0.3838131	0.031024	1.508303035
ppapdc1b		15.60666667	21.07666667	0.740471	-0.4334843	0.031803	1.497533319
ppcdc	ENSDARG00000033020	6.163333333	2.62	2.352417	1.234144	0.036196	1.441335978
ppdpfa	ENSDARG00000007682	30.15666667	101.38	0.297462	-1.7492242	0.013792	1.860377418
PPFIBP1 (2 of 2)	ENSDARG00000100057	7.76	5.043333333	1.538665	0.6216791	0.005146	2.288567717
ppp1r10	ENSDARG00000032651	18.57333333	21.48	0.86468	-0.2097612	0.014964	1.824941555
ppp1r14bb	ENSDARG00000030161	23.2	15.99	1.450907	0.5369549	0.024824	1.605128661
ppp1r18	ENSDARG00000071251	1.91	5.51	0.346642	-1.5284797	0.001152	2.938442754
ppp1r37	ENSDARG00000078458	5.48	8.206666667	0.66775	-0.5826205	0.024509	1.610679069
ppp1r7	ENSDARG00000009740	70.2	48.74	1.440295	0.5263648	0.021746	1.662622963
ppp2r2cb	ENSDARG00000062277	4.066666667	7.78	0.522708	-0.9359234	0.047233	1.325756782
ppp2r5cb	ENSDARG00000031200	15.53	17.84666667	0.870191	-0.2005968	0.004726	2.325512272
ppp2r5d	ENSDARG00000014428	10.19	16.55	0.61571	-0.6996772	0.011446	1.941343532
ppp2r5ea	ENSDARG00000015474	12.82333333	20.75333333	0.617893	-0.6945717	0.019392	1.712384714
ppp3cb	ENSDARG00000025106	23.54333333	41.82666667	0.562879	-0.8291044	0.023264	1.633315225
ppp4cb	ENSDARG00000076439	34.9	46.51333333	0.750322	-0.4144173	0.012943	1.887957968
pqlc2	ENSDARG00000043624	12.51666667	7.253333333	1.725643	0.7871344	0.022948	1.639248679
praf2	ENSDARG00000032535	34.32333333	23.87	1.437928	0.5239911	0.027287	1.564039894
prcc	ENSDARG00000063252	10.76666667	15.64666667	0.688112	-0.5392837	0.002561	2.591635332
prdm10	ENSDARG00000104251	3.446666667	5.013333333	0.6875	-0.5405684	0.004148	2.382155177
prdm8	ENSDARG00000025017	6.693333333	3.346666667	2	1	0.043348	1.363035024
prkacaa	ENSDARG00000100349	24.45	30.65333333	0.797629	-0.3262095	0.022869	1.64075648
prkag2a	ENSDARG00000012625	4.96	8.096666667	0.612598	-0.706988	0.01295	1.887725866
prkar2aa	ENSDARG00000033184	28.15666667	56.16666667	0.501306	-0.9962376	3.79E-05	4.421848691
prkcbb	ENSDARG00000022254	1.376666667	3.43	0.401361	-1.3170293	0.026111	1.583181945
prkcea	ENSDARG00000003008	6.916666667	10.15	0.681445	-0.5533309	0.02501	1.601892045
prkcsh	ENSDARG00000004470	47.81666667	40.02333333	1.19472	0.2566722	0.025827	1.587917748
prkx	ENSDARG00000060716	10.67	6.536666667	1.63233	0.7069331	0.034722	1.459392146
prmt1	ENSDARG00000010246	165.32	145.1966667	1.138594	0.1872529	0.035867	1.445299078
prmt8b	ENSDARG00000045760	5.82	9.19	0.633297	-0.6590457	0.031848	1.496915893
prnprs3	ENSDARG00000003705	18.70333333	30.57333333	0.611753	-0.7089784	0.02897	1.538047845

prom1a	ENSDARG00000039966	4.15	7.51	0.552597	-0.8557016	0.042125	1.375457561
prph	ENSDARG00000028306	28.62333333	12.38333333	2.31144	1.208792	0.000725	3.139905266
prph2a	ENSDARG00000038018	9.623333333	49.39666667	0.194817	-2.3598051	0.004498	2.346955751
prph2l	ENSDARG00000021345	1.743333333	5.853333333	0.297836	-1.74741	0.007578	2.120465956
prrc1	ENSDARG00000044304	23.01333333	14.93	1.541415	0.6242558	0.000981	3.008183758
prrc2a	ENSDARG00000031494	25.42333333	31.31333333	0.811901	-0.3006239	0.040737	1.390013332
prss35	ENSDARG00000100691	51.36	27.62666667	1.859073	0.8945837	0.048545	1.313852452
prune2	ENSDARG00000059423	12.20666667	16.09333333	0.758492	-0.3987939	0.037573	1.425129137
psap	ENSDARG00000013968	75.67	107.7933333	0.701991	-0.5104746	0.005789	2.237398054
psma2	ENSDARG00000040121	85.53	60.38666667	1.416372	0.5022005	0.029281	1.533415282
psma6a	ENSDARG00000019398	123.0233333	65.16333333	1.887923	0.9167997	0.041837	1.378435147
psmc1a	ENSDARG00000030537	87.33	59.12	1.477165	0.5628311	0.00565	2.247963045
psmc4	ENSDARG00000027099	108.9133333	79.8	1.364829	0.4487199	0.030456	1.516327733
psmd4a	ENSDARG00000101295	90.04666667	59.55	1.512119	0.5965714	0.015472	1.81045704
psmd7	ENSDARG00000102417	128.2433333	82.42333333	1.555911	0.6377591	0.048986	1.309926615
ptenb	ENSDARG00000056623	13.15666667	17.02666667	0.772709	-0.372002	0.040985	1.38737429
ptmaa	ENSDARG00000021113	3057.656667	1984.64	1.540661	0.6235491	0.031364	1.503565819
ptprna	ENSDARG00000058646	16.11666667	23.30666667	0.691505	-0.5321893	0.003505	2.455305142
pus10	ENSDARG00000079973	3.373333333	5.253333333	0.642132	-0.6390582	0.043177	1.364745538
pvalb6	ENSDARG00000009311	36.00333333	91.15	0.39499	-1.3401122	0.018443	1.734175062
pwp2h	ENSDARG00000037109	10.93666667	8.06	1.356907	0.4403213	0.000758	3.120420615
pycr1a	ENSDARG00000102254	4.46	8.866666667	0.503008	-0.9913481	0.012151	1.915396127
r3hdm1	ENSDARG00000101458	48.76666667	83	0.58755	-0.767216	0.029086	1.536308819
rab11fip1a	ENSDARG00000046124	4.84	2.71	1.785978	0.8367142	0.002519	2.598796125
rab2a	ENSDARG00000020261	139.7766667	155.3266667	0.899888	-0.152182	0.010582	1.975414004
rab3c	ENSDARG00000014462	24.54666667	36.59333333	0.670796	-0.5760537	0.008065	2.093371956
rab3gap2	ENSDARG00000044136	3.213333333	5.383333333	0.596904	-0.7444291	0.02985	1.525062086
rab41	ENSDARG00000029885	43.28	59.94666667	0.721975	-0.469979	0.022474	1.648316177
rab6ba	ENSDARG00000034522	21.99	38.26	0.574752	-0.7989893	0.024039	1.619086807
rab6bb	ENSDARG00000031343	32.65	70.87	0.460703	-1.1180921	0.005708	2.243498486
rac3b	ENSDARG00000020795	35.49666667	59.82	0.593391	-0.7529444	0.025236	1.597973732
rad50	ENSDARG00000038917	2.713333333	1.906666667	1.423077	0.5090136	0.025494	1.593556911
rad51ap1	ENSDARG00000045853	7.346666667	3.736666667	1.966102	0.9753379	0.03229	1.490927482
RAI1	ENSDARG00000076679	4.433333333	8.21	0.539992	-0.8889904	0.020658	1.684916324
ralgps1	ENSDARG00000068370	2.463333333	4.343333333	0.567153	-0.8181908	0.029815	1.525568772
rangrf	ENSDARG00000098786	9.113333333	6.723333333	1.355478	0.4388022	0.026258	1.580732513
RAPGEF4 (1 of 2)	ENSDARG00000075924	4.723333333	8.74	0.540427	-0.8878279	0.02112	1.675311068
rassf2a	ENSDARG00000029865	17.38666667	11.59666667	1.499281	0.5842712	0.023492	1.62908601
rassf6	ENSDARG00000000804	11.16	7.466666667	1.494643	0.5798008	0.00229	2.640091096
rbl2	ENSDARG00000045636	1.896666667	4.303333333	0.440744	-1.1819884	0.001391	2.856647706
rbm12b	ENSDARG00000079717	12.11	15.02666667	0.805901	-0.3113262	0.040628	1.391174191
rbm22	ENSDARG00000010238	20.06333333	26.31	0.762574	-0.3910499	0.042733	1.369237531
rbm39b	ENSDARG00000041853	19.4	26.55	0.730697	-0.4526552	0.043879	1.357742097
rbm6	ENSDARG00000077060	8.35	12.12	0.688944	-0.5375416	0.047433	1.323919927
RBP1 (2 of 2)	ENSDARG00000038742	6.723333333	23.43	0.286954	-1.8011084	0.043222	1.364292612
rbp4l	ENSDARG00000044684	49.02666667	134.98	0.363214	-1.4611071	0.028413	1.546486555
rcan3	ENSDARG00000032623	11.22	18.22666667	0.615582	-0.6999781	0.048612	1.313258787
rccd1	ENSDARG00000104505	11.28333333	0.39	28.93162	4.8545754	0.01601	1.795618793
rchy1	ENSDARG00000016710	12.64	18.65666667	0.677506	-0.5616948	0.042532	1.371283536
RDH13 (3 of 3)	ENSDARG00000054797	4.626666667	12.78333333	0.36193	-1.466219	0.020303	1.692436772
rdh8a	ENSDARG00000028048	18.52333333	44.19	0.419175	-1.2543762	0.027603	1.559038644
rdh8b	ENSDARG00000105060	2.696666667	6.346666667	0.424895	-1.2348219	0.030547	1.51502965
rfc5	ENSDARG00000035634	36.99	25.98666667	1.423422	0.5093637	0.01047	1.980041489
rgl1	ENSDARG00000005989	9.67	6.006666667	1.609878	0.6869513	0.026887	1.570456783

rgs20	ENSDARG00000038859	3.273333333	10.00333333	0.327224	-1.6116484	0.001071	2.970359271
rgs7bpa	ENSDARG00000060601	9.166666667	15.63333333	0.586354	-0.7701563	0.032024	1.494530478
rgs8	ENSDARG00000070037	18.64333333	28.80333333	0.647263	-0.627576	0.045433	1.34263109
rhbdl3	ENSDARG00000105190	7.223333333	5.456666667	1.323763	0.4046448	0.031953	1.495489569
rheb	ENSDARG00000090213	23.66	34.99666667	0.676064	-0.5647674	0.043169	1.364828694
ric8a	ENSDARG00000007247	9.356666667	17.56666667	0.532638	-0.9087739	0.004702	2.327714892
rims2a	ENSDARG00000101606	12.63	23.88666667	0.528747	-0.9193509	0.013939	1.85576507
rims2b	ENSDARG00000102690	8.196666667	17.16666667	0.477476	-1.0665007	0.046817	1.329591826
riok1	ENSDARG00000016399	13.64	9.493333333	1.436798	0.522857	0.010854	1.964394116
rlbp1a	ENSDARG00000012504	42.58333333	81.26	0.524038	-0.9322565	0.048207	1.31689245
rlf	ENSDARG00000063553	3.38	4.846666667	0.697387	-0.5199696	0.020417	1.68999889
rltgr	ENSDARG00000096533	5.85	13.55333333	0.431628	-1.2121392	0.035226	1.453131927
rltpr	ENSDARG00000056639	9.533333333	16.40666667	0.581065	-0.7832295	0.030401	1.517117646
rmnd5b	ENSDARG00000043359	15.84666667	18.64666667	0.849839	-0.2347384	0.009936	2.002779706
rnasekb	ENSDARG00000104458	186.6133333	280.22	0.665953	-0.5865079	0.00791	2.101801424
rnd1l		17.21666667	22.09666667	0.779152	-0.3600229	0.003197	2.495324776
rnf11a	ENSDARG00000010442	8.436666667	10.1	0.835314	-0.2596103	0.023823	1.622995866
rock2a	ENSDARG00000017500	3.973333333	3.023333333	1.314223	0.3942098	0.043189	1.364631554
romo1	ENSDARG00000038076	300.3866667	224.3333333	1.339019	0.4211768	0.030733	1.512394148
roraa	ENSDARG00000031768	6.046666667	11.21	0.539399	-0.8905743	0.026258	1.580733416
rorab	ENSDARG00000001910	24.38333333	38.74666667	0.629301	-0.6681768	0.022308	1.651533871
rorcb	ENSDARG00000017780	2.306666667	3.796666667	0.60755	-0.7189238	0.037941	1.420891766
rp1l1a	ENSDARG00000089458	1.35	2.356666667	0.572843	-0.8037883	0.007956	2.099291719
rpe	ENSDARG00000005251	25.33333333	16.78	1.509734	0.5942942	0.034978	1.456201187
rpgrip1	ENSDARG00000076055	2.076666667	4.22	0.492101	-1.0229733	0.009989	2.000480225
rpia	ENSDARG00000056640	8.546666667	12.47666667	0.685012	-0.5457988	0.031061	1.507780277
rpl11	ENSDARG00000043509	5316.053333	3027.506667	1.755918	0.8122254	0.023246	1.633648971
rpl12	ENSDARG00000006691	2847.146667	2167.37	1.313641	0.3935714	0.000157	3.802725711
rpl14	ENSDARG00000103433	1800.653333	1450.55	1.241359	0.3119204	0.017789	1.749854586
rpl22	ENSDARG00000070437	2117.206667	1460.02	1.450122	0.536174	0.003823	2.417630996
rpl22l1	ENSDARG00000010244	1410.44	740.7166667	1.904156	0.9291516	0.004502	2.346563924
rpl23	ENSDARG00000053457	3010.21	2221.81	1.354846	0.4381287	0.000976	3.010507525
rpl23a	ENSDARG00000006316	3159.723333	2468.62	1.279955	0.3560935	0.039729	1.400895104
rpl27	ENSDARG00000015128	4384.763333	3112.523333	1.408749	0.4944143	0.036946	1.43242722
rpl30	ENSDARG00000035871	5562.916667	3301.283333	1.685077	0.7528145	0.002599	2.585223104
rpl32	ENSDARG00000054818	5860.373333	4031.683333	1.45358	0.5396102	0.00197	2.705613998
rpl36	ENSDARG00000100588	4082.226667	2021.693333	2.019212	1.0137921	0.035166	1.453877798
rpl36a	ENSDARG00000058105	2425.053333	1843.083333	1.315759	0.3958952	0.012644	1.89813098
rpl37	ENSDARG00000034291	5285.37	3633.023333	1.454813	0.5408338	0.03003	1.52245006
rpl38	ENSDARG00000006413	1354.243333	1111.113333	1.218817	0.285481	0.023611	1.626879885
rpl5a	ENSDARG00000020197	2798.236667	2015.666667	1.388244	0.4732609	0.008386	2.076449333
rpl7	ENSDARG00000007320	4269.62	2729.576667	1.564206	0.6454305	0.001639	2.785300087
rpl9	ENSDARG00000037350	3506.79	2232.8	1.57058	0.651297	0.003436	2.464005805
rps11	ENSDARG00000053058	2556.776667	1644.95	1.554319	0.6362824	0.005857	2.232306931
rps15a	ENSDARG00000010160	3658.13	2339.123333	1.563889	0.6451384	0.004281	2.368492744
rps20	ENSDARG00000036044	7870.7	5739.823333	1.371244	0.4554856	0.010007	1.999708976
rps24	ENSDARG00000039347	2528.75	1469.05	1.721351	0.7835409	0.00069	3.160910295
rps27.1	ENSDARG00000023298	5739.846667	2693.236667	2.131208	1.0916712	0.00141	2.850880422
rps27.2	ENSDARG00000055475	3753.9	2290.896667	1.638616	0.7124778	0.005551	2.255627627
rps29	ENSDARG00000041232	5933.29	4773.236667	1.243033	0.3138644	0.048021	1.318571968
rps7	ENSDARG00000042566	4689.72	3081.913333	1.521691	0.6056755	0.018503	1.732753673
rpz5	ENSDARG00000075718	24.00666667	14.69333333	1.633848	0.7082734	0.035909	1.444792893
rrad	ENSDARG00000052011	4.306666667	8.61	0.500194	-0.9994416	0.008038	2.094838142
rragca	ENSDARG00000069829	7.9	11.80333333	0.669302	-0.5792698	0.048583	1.31351981

rrm2	ENSDARG00000078069	118.26	51.29	2.305713	1.2052127	0.023651	1.626156941
rrp36	ENSDARG00000032516	4.673333333	8.18	0.571312	-0.8076489	0.016247	1.78923764
rs1a	ENSDARG00000027236	132.84	281.3933333	0.472079	-1.0828985	0.024837	1.604908549
rsl24d1	ENSDARG00000040439	411.3066667	259.7266667	1.583614	0.6632203	0.009383	2.02766264
rsrp1	ENSDARG00000030440	10.45	30.01	0.348217	-1.5219404	0.001986	2.701935701
rtcb	ENSDARG00000101047	7.386666667	12.95666667	0.570105	-0.8106992	0.040662	1.390813023
rufy2	ENSDARG00000062019	9.38	12.07333333	0.776919	-0.3641642	0.037574	1.42511115
ruvbl1	ENSDARG00000002591	46.45	32.03333333	1.450052	0.5361047	0.01265	1.897902708
rwdd	ENSDARG00000068256	34.61	21.27666667	1.626665	0.7019168	0.01431	1.844358454
rwdd1	ENSDARG00000015930	46.15333333	41.94	1.100461	0.138108	0.02013	1.696159317
rx1	ENSDARG00000071684	16.18333333	28.29	0.572051	-0.8057834	0.023464	1.629591208
sall1a	ENSDARG00000074319	13.49	22.53	0.598757	-0.739957	0.001703	2.768712047
SAMD4B	ENSDARG00000086735	2.66	8.71	0.305396	-1.7112465	0.04248	1.371810718
samsn1b	ENSDARG00000078647	0.98	4.253333333	0.230408	-2.1177403	0.014891	1.827069739
sap18	ENSDARG00000057854	186.6733333	147.0033333	1.269858	0.344667	0.031852	1.496859765
sars	ENSDARG00000008237	91.74666667	72.43	1.266694	0.3410684	0.049494	1.305449229
sbds	ENSDARG00000027803	12.24666667	16.73	0.732018	-0.4500483	0.044863	1.348111108
sbf1	ENSDARG00000062968	11.68333333	15.47333333	0.755062	-0.4053321	0.003703	2.431476804
sc:d217	ENSDARG00000079645	8.146666667	3.986666667	2.043478	1.0310269	0.03252	1.487851585
scamp4	ENSDARG00000010531	6.85	4.853333333	1.411401	0.497128	0.011163	1.952233794
SCARNA13	ENSDARG00000083738	4.466666667	0.73	6.118721	2.6132302	0.00411	2.386150048
scg3	ENSDARG00000086288	45.45666667	68.96333333	0.659143	-0.6013376	0.009478	2.023286771
scp2a	ENSDARG00000012194	8.026666667	12.17333333	0.659365	-0.6008514	0.025394	1.59527212
scrn2	ENSDARG00000043497	9.17	23.57333333	0.388999	-1.3621621	0.004256	2.370978988
sdc4	ENSDARG00000059906	66.51	41.67666667	1.595857	0.6743314	0.016246	1.789243152
sdf2l1	ENSDARG00000035631	74.63666667	54.78333333	1.362397	0.4461475	0.034561	1.461419557
sec11a	ENSDARG00000008936	134.3866667	93.65	1.434988	0.5210391	0.033226	1.478521412
sec24c	ENSDARG00000103516	7.306666667	11.54666667	0.632794	-0.6601911	0.027971	1.553297708
sec61a1	ENSDARG00000021669	177.9666667	127.48	1.396036	0.4813361	0.023864	1.622253643
sec61b	ENSDARG00000076568	349.4666667	258.1466667	1.353752	0.4369639	0.003149	2.501771683
sel1l	ENSDARG00000004581	17.62333333	15.05	1.170986	0.2277233	0.014572	1.836478067
selt1b		33.33666667	25.22666667	1.321485	0.4021603	0.002741	2.562162821
senp7b	ENSDARG00000061138	2.68	4.31	0.62181	-0.6854549	0.033502	1.474927014
sepn1	ENSDARG00000033616	46.14333333	24.25666667	1.902295	0.9277409	0.003725	2.428856114
sepw1	ENSDARG00000035136	370.1933333	259.2333333	1.428031	0.5140277	0.01944	1.711294623
sepw2a		155.0133333	114.1933333	1.357464	0.4409139	0.032294	1.490872681
serp1	ENSDARG00000099439	136.2166667	101.9633333	1.335938	0.4178528	0.041508	1.381867581
setd2	ENSDARG00000062244	4.816666667	6.836666667	0.704534	-0.505258	0.039375	1.40477918
sez6a	ENSDARG00000100876	2.52	5.896666667	0.42736	-1.2264759	0.008683	2.061329291
sf1	ENSDARG00000008188	39.63666667	58.90333333	0.67291	-0.5715136	0.048169	1.317235073
sf3b5	ENSDARG00000016855	147.27	89.64333333	1.642844	0.7161954	0.003303	2.48113944
sf3b6	ENSDARG00000009753	195.01	109.8766667	1.774808	0.8276631	0.018918	1.723130545
sgip1a	ENSDARG00000097897	7.633333333	12.53333333	0.609043	-0.7153851	0.040037	1.397541498
sh3bgrl2	ENSDARG00000036878	8.296666667	16.14	0.514044	-0.9600368	0.029662	1.527806311
sh3bp5b	ENSDARG00000103978	12.79	16.76333333	0.762975	-0.3902928	0.018835	1.725038737
sh3gl2	ENSDARG00000023600	22.65	41.17666667	0.550069	-0.862316	0.047676	1.321695702
sh3kbp1	ENSDARG00000075853	3.483333333	6.026666667	0.577987	-0.7908917	0.03388	1.470052201
shha	ENSDARG00000068567	14.94333333	13.08333333	1.142166	0.1917718	0.013429	1.871948985
shmt1	ENSDARG00000052816	21.94333333	18.00666667	1.218623	0.2852516	0.029246	1.533935685
shroom4	ENSDARG00000079900	5.526666667	3.543333333	1.559737	0.6413024	0.014438	1.84048827
si:busm1-234g15.3	ENSDARG00000098051	0.34	2.28	0.149123	-2.7454272	0.007413	2.129984068
si:cabz01016011.1	ENSDARG00000098459	0.74	4.823333333	0.153421	-2.7044333	0.034169	1.466368826
si:ch1073-147e4.1	ENSDARG00000096978	5.373333333	2.476666667	2.169583	1.1174176	0.03069	1.513002338
si:ch1073-174d20.[illegible]	ENSDARG00000079651	7.753333333	8.806666667	0.880394	-0.1837794	0.038831	1.410822113

si:ch1073-190k2.1	ENSDARG00000097973	23.41	4.363333333	5.365164	2.4236223	9.30E-05	4.031464399
si:ch1073-235i16.2	ENSDARG00000097189	2.616666667	1.14	2.295322	1.1986963	0.012648	1.897975777
si:ch1073-303d10.1	ENSDARG00000103006	1.526666667	7.463333333	0.204556	-2.289435	0.044424	1.352385944
si:ch1073-303k11.2	ENSDARG00000088247	15.05666667	11.59666667	1.298362	0.3766922	0.014805	1.829599812
si:ch1073-322p19.1	ENSDARG00000075173	5.593333333	9.423333333	0.593562	-0.7525292	0.01626	1.788866945
si:ch1073-385i9.2	ENSDARG00000063300	2.913333333	5.263333333	0.553515	-0.853306	0.048965	1.310111128
si:ch1073-39g8.1	ENSDARG00000096174	2.323333333	0.353333333	6.575472	2.7170944	0.006111	2.213919367
si:ch1073-469d17.2	ENSDARG00000101485	9.453333333	32.26	0.293036	-1.7708514	0.001086	2.964074224
si:ch211-105c13.3	ENSDARG00000089441	108.2033333	27.26	3.969308	1.9888875	0.017599	1.754511158
si:ch211-106m9.2	ENSDARG00000105163	1.413333333	8.506666667	0.166144	-2.5894922	0.014222	1.847024314
si:ch211-107p11.3	ENSDARG00000103000	16.73	9.916666667	1.687059	0.7545103	0.047878	1.319859665
si:ch211-113g11.6	ENSDARG00000070442	6.713333333	9.966666667	0.673579	-0.5700818	0.031917	1.495976872
si:ch211-114l13.11	ENSDARG00000092358	0.046666667	12.63	0.003695	-8.0802465	0.005666	2.24675543
si:ch211-114n24.7		7.65	10.76333333	0.710746	-0.4925933	0.033478	1.475234364
si:ch211-117c9.5	ENSDARG00000071235	12.30666667	17.75666667	0.693073	-0.5289207	0.034304	1.464653255
si:ch211-119e14.1	ENSDARG00000099518	2.503333333	1.126666667	2.221893	1.1517897	0.006761	2.16998458
si:ch211-11k18.4	ENSDARG00000087869	29.29	21.76666667	1.345636	0.4282877	0.03902	1.40870758
si:ch211-12c3.3	ENSDARG00000104154	2.933333333	4.62	0.634921	-0.6553518	0.036991	1.431898299
si:ch211-132g1.3	ENSDARG00000089477	8.036666667	12.02	0.668608	-0.5807677	0.011125	1.953691438
si:ch211-145b13.6	ENSDARG00000069998	3.47	1.42	2.443662	1.2890447	0.003004	2.52228572
si:ch211-147a11.3	ENSDARG00000069009	18.87	13.59333333	1.38818	0.4731951	0.007485	2.125826916
si:ch211-150o23.3	ENSDARG00000078502	11.75666667	9.763333333	1.204165	0.2680334	0.027572	1.559525618
si:ch211-152c2.3	ENSDARG00000045898	35.27333333	18.90333333	1.865985	0.8999373	0.035643	1.448021052
si:ch211-160d14.9	ENSDARG00000096599	3.18	0.42	7.571429	2.9205655	0.003922	2.406509281
si:ch211-167m1.5	ENSDARG00000097670	1.103333333	4.246666667	0.259812	-1.9444622	0.037528	1.42564669
si:ch211-175f12.2	ENSDARG00000095831	1.476666667	4.323333333	0.341557	-1.5497999	0.001028	2.988035174
si:ch211-195b15.8	ENSDARG00000094836	15.71333333	21.01	0.747898	-0.4190869	0.002511	2.60007529
si:ch211-198p11.6	ENSDARG00000097765	8.103333333	4.86	1.667353	0.7375592	0.025753	1.589176052
si:ch211-202a12.4	ENSDARG00000002369	325.8833333	191.9966667	1.697338	0.7632743	0.017483	1.757379658
si:ch211-202e12.3	ENSDARG00000095715	44.17666667	29.87333333	1.478799	0.5644263	0.0366	1.436513182
si:ch211-207l14.1	ENSDARG00000098376	9.073333333	26.03	0.348572	-1.5204707	0.048309	1.315974247
si:ch211-209a2.1	ENSDARG00000094097	19.87666667	8.646666667	2.298766	1.2008599	0.003168	2.499195034
si:ch211-210c8.6	ENSDARG00000017811	8.816666667	7.11	1.240038	0.3103838	0.032607	1.48668879
si:ch211-221g5.2	ENSDARG00000095646	2.993333333	0.213333333	14.03125	3.8105716	0.043193	1.364582712
si:ch211-223a21.1	ENSDARG00000098892	0.52	4.936666667	0.105334	-3.2469537	0.046274	1.334664401
si:ch211-229l10.12	ENSDARG00000101760	11.18	0.733333333	15.24545	3.9303073	0.048741	1.312109788
si:ch211-232m10.6	ENSDARG00000070931	0.996666667	5.08	0.196194	-2.3496455	0.001441	2.841239712
si:ch211-233a24.2	ENSDARG00000062330	2.856666667	4.45	0.641948	-0.6394726	0.004653	2.332226954
si:ch211-235i11.4	ENSDARG00000088298	2.52	3.72	0.677419	-0.5618789	0.024689	1.607495698
si:ch211-236d3.4	ENSDARG00000086300	2.11	0.806666667	2.615702	1.3871985	0.03009	1.521583679
si:ch211-239d6.4	ENSDARG00000100012	13.37666667	28.02	0.477397	-1.0667383	0.030831	1.511016059
si:ch211-241e1.5	ENSDARG00000008906	2.92	2.11	1.383886	0.4687254	0.025271	1.597382797
si:ch211-244c8.4	ENSDARG00000103071	12.87333333	19.58666667	0.65725	-0.6054862	0.015446	1.811192412
si:ch211-248g18.6	ENSDARG00000097211	17.49666667	9.693333333	1.805021	0.8520153	0.044028	1.356272701
si:ch211-254n4.3	ENSDARG00000020292	6.356666667	11.68333333	0.54408	-0.8781096	0.00585	2.232837477
si:ch211-259g3.4	ENSDARG00000030945	2.396666667	2.973333333	0.806054	-0.3110519	0.040272	1.394998519
si:ch211-260e23.9	ENSDARG00000102572	70.32	44.36333333	1.585093	0.6645673	0.021532	1.666922669
si:ch211-265m20.8	ENSDARG00000093535	6.543333333	2.473333333	2.645553	1.4035691	0.027979	1.553165747
si:ch211-274k16.4	ENSDARG00000095786	1.016666667	2.1	0.484127	-1.0465426	0.010235	1.989931668
si:ch211-286b5.5	ENSDARG00000096661	67.06	45.40666667	1.476876	0.5625484	0.026836	1.571277507
si:ch211-30g3.8	ENSDARG00000099019	0.826666667	4.593333333	0.179971	-2.4741639	0.021361	1.670376928
si:ch211-3o3.9	ENSDARG00000097855	1.933333333	4.42	0.437406	-1.192956	0.005883	2.230418818
si:ch211-5k11.2	ENSDARG00000088330	31.76	82.42	0.385343	-1.3757836	0.039878	1.399262621
si:ch211-63o20.7	ENSDARG00000042000	5.096666667	2.016666667	2.527273	1.3375814	0.004787	2.319905059

si:ch73-119p20.1	ENSDARG00000099257	24.02	51.79333333	0.463766	-1.1085303	0.020267	1.693215871
si:ch73-126m5.4	ENSDARG00000096857	5.743333333	2.996666667	1.916574	0.9385297	0.047531	1.323020268
si:ch73-144d13.4	ENSDARG00000086223	6.836666667	12.39666667	0.551492	-0.8585873	0.020349	1.691465906
si:ch73-166c6.2	ENSDARG00000098395	10.39666667	5.07	2.050625	1.0360634	0.021847	1.660599659
si:ch73-215f7.1	ENSDARG00000098512	29.64666667	8.903333333	3.329839	1.7354524	0.001017	2.992507185
si:ch73-21g5.7	ENSDARG00000093156	97.9	20.17	4.853743	2.2790978	0.001745	2.758147169
si:ch73-21k16.6	ENSDARG00000096770	16.98666667	24.48666667	0.693711	-0.5275936	0.047494	1.323361814
si:ch73-233f7.1	ENSDARG00000061909	6.3	11.11666667	0.566717	-0.8193005	0.016243	1.78933445
si:ch73-234b20.6	ENSDARG00000092880	1.536666667	5.6	0.274405	-1.8656226	0.036707	1.435255529
si:ch73-234i15.7	ENSDARG00000103059	5.336666667	1.743333333	3.061185	1.6140905	0.043852	1.358009154
si:ch73-23l24.1	ENSDARG00000096905	120.1566667	80.77	1.48764	0.5730253	0.031948	1.495556436
si:ch73-274k23.3	ENSDARG00000097279	40.05333333	96.82	0.413689	-1.2733828	0.019462	1.710820424
si:ch73-28h20.1	ENSDARG00000098392	25.22	44.55	0.566105	-0.8208572	0.002897	2.53809903
si:ch73-29l19.1	ENSDARG00000100255	5.146666667	9.533333333	0.53986	-0.8893424	0.017992	1.744928874
si:ch73-305o9.3	ENSDARG00000101501	1.17	2.49	0.46988	-1.0896372	0.020358	1.691274902
si:ch73-386o14.1	ENSDARG00000091917	0.866666667	3.39	0.255654	-1.9677362	0.00785	2.105120036
si:ch73-389b16.2	ENSDARG00000103802	3.65	8.426666667	0.433149	-1.2070656	0.024264	1.615035803
si:ch73-44m9.2	ENSDARG00000094821	2.41	0.573333333	4.203488	2.0715871	0.014421	1.84100433
si:ch73-44m9.3	ENSDARG00000100357	8.03	11.60333333	0.692043	-0.5310674	0.008119	2.090510379
si:dkey-100n10.2	ENSDARG00000062672	1.736666667	2.733333333	0.635366	-0.6543405	0.040952	1.387720915
si:dkey-109j17.5	ENSDARG00000104891	2.393333333	3.45	0.69372	-0.527575	0.017267	1.762778593
si:dkey-111e8.5	ENSDARG00000093960	3.073333333	0.243333333	12.63014	3.6587984	0.01877	1.726540383
si:dkey-111k8.6	ENSDARG00000096851	8.98	4.676666667	1.920171	0.9412348	0.017292	1.762156539
si:dkey-121a11.3	ENSDARG00000071458	1.983333333	3.513333333	0.564516	-0.8249133	0.006376	2.195465377
si:dkey-12n3.1	ENSDARG00000098721	0	2.223333333	0.310238	-1.6885534	0.015451	1.811051271
si:dkey-145c18.5	ENSDARG00000093764	3.233333333	0.903333333	3.579336	1.8396919	0.01399	1.854194924
si:dkey-14d8.23	ENSDARG00000097507	7.703333333	2.26	3.408555	1.7691601	0.028211	1.549581076
si:dkey-153k10.9	ENSDARG00000004386	11.10333333	17.14333333	0.647676	-0.6266548	0.046822	1.329553559
si:dkey-153m14.1	ENSDARG00000096403	1586.226667	6437.856667	0.24639	-2.0209815	0.001918	2.717101896
si:dkey-165g20.5	ENSDARG00000094933	2.5	24.60333333	0.101612	-3.2988538	0.039589	1.402427456
si:dkey-165n16.1	ENSDARG00000087048	11.02	5.43	2.029466	1.0211001	0.018785	1.726187409
si:dkey-16n15.3	ENSDARG00000092373	3.116666667	1.703333333	1.829746	0.8716431	0.017857	1.74820038
si:dkey-177p2.6	ENSDARG00000042322	9.11	15.09666667	0.603444	-0.7287071	0.040982	1.387411093
si:dkey-181d19.3	ENSDARG00000097761	0.556666667	3.456666667	0.161041	-2.6344959	0.022297	1.651757733
si:dkey-182i3.10	ENSDARG00000096614	4.403333333	2.793333333	1.576372	0.6566083	0.026365	1.578974148
si:dkey-184p9.7	ENSDARG00000092726	4.78	2.093333333	2.283439	1.1912086	0.020679	1.68447122
si:dkey-187a12.4	ENSDARG00000091897	0.773333333	3.633333333	0.212844	-2.2321314	0.013209	1.879120619
si:dkey-193c22.1	ENSDARG00000093687	4.84	7.216666667	0.67067	-0.5763256	0.009763	2.0104178
si:dkey-193h7.5	ENSDARG00000092633	0.66	10.89	0.060606	-4.0443941	1.08E-05	4.965374115
si:dkey-201g16.1	ENSDARG00000101292	0	2.633333333	0.275229	-1.8612937	0.016593	1.780080815
si:dkey-206f10.1	ENSDARG00000032838	1.363333333	4.776666667	0.285415	-1.8088659	0.003115	2.506520417
si:dkey-21e13.3	ENSDARG00000096701	1.413333333	3.093333333	0.456897	-1.1300605	0.024592	1.609203132
si:dkey-222p3.1	ENSDARG00000071103	4.186666667	0.53	7.899371	2.9817378	0.005486	2.260737513
si:dkey-224k5.13	ENSDARG00000105162	10.18333333	6.573333333	1.549189	0.6315128	0.022602	1.645852172
si:dkey-237m9.3	ENSDARG00000098071	24.05	86.84	0.276946	-1.8523228	0.009238	2.034424707
si:dkey-238c7.12	ENSDARG00000095556	335.0866667	1203.033333	0.278535	-1.8440704	0.048202	1.316939341
si:dkey-239h2.3	ENSDARG00000092033	28.29666667	10.51666667	2.69065	1.4279546	0.002697	2.569160541
si:dkey-23i12.5	ENSDARG00000103682	40.33	26.18666667	1.540097	0.623021	0.001928	2.714859631
si:dkey-242k1.4	ENSDARG00000104899	0	2.71	0.269542	-1.8914192	0.016225	1.789807508
si:dkey-244a7.1	ENSDARG00000097368	8.583333333	2.736666667	3.136419	1.6491183	0.020227	1.694077586
si:dkey-253i9.4	ENSDARG00000061459	5.42	11.5	0.471304	-1.0852691	0.003965	2.401777891
si:dkey-270b7.4	ENSDARG00000100537	0.63	2.13	0.295775	-1.7574297	0.040849	1.388813821
si:dkey-285e18.8	ENSDARG00000090116	30.13666667	17.99333333	1.67488	0.7440574	0.01301	1.885715906
si:dkey-28b4.8	ENSDARG00000002840	27.71666667	36	0.769907	-0.3772431	0.021007	1.677627078

si:dkey-30c15.10	ENSDARG00000088130	5.36	3.636666667	1.473877	0.5596163	0.009437	2.025146744
si:dkey-31c13.1	ENSDARG00000104058	14.45333333	7.906666667	1.827993	0.8702607	0.049541	1.305031789
si:dkey-42i9.4	ENSDARG00000008049	206.8566667	85.02333333	2.43294	1.2827007	0.002536	2.59588418
si:dkey-45l12.2	ENSDARG00000097129	6.726666667	0.64	10.51042	3.393748	0.028979	1.537911325
si:dkey-4c15.15	ENSDARG00000071819	0.823333333	3.813333333	0.215909	-2.2115041	0.043814	1.358389234
si:dkey-58f10.7	ENSDARG00000091972	3.14	1.053333333	2.981013	1.5758025	0.026125	1.582951806
si:dkey-60a16.1	ENSDARG00000037073	5.286666667	3.52	1.501894	0.5867829	0.013018	1.885443029
si:dkey-61o18.2	ENSDARG00000096876	0.616666667	4.28	0.144081	-2.795048	0.023506	1.628819624
si:dkey-71b5.3	ENSDARG00000095522	0.013333333	2.11	0.006319	-7.3060617	0.008875	2.05182483
si:dkey-71b5.7	ENSDARG00000080675	0.49	4.066666667	0.120492	-3.0529931	0.011896	1.92460058
si:dkey-77f5.3	ENSDARG00000087331	21.08666667	12.96	1.627058	0.7022653	0.015983	1.796335908
si:dkey-77p23.3	ENSDARG00000103031	2.083333333	0	3.083333	1.6244909	0.0023	2.638283454
si:dkey-81l17.6	ENSDARG00000097322	36.77666667	51.17	0.718715	-0.4765075	0.016584	1.780316793
si:dkey-9c18.3	ENSDARG00000096579	13.76	4.413333333	3.117825	1.6405398	0.030463	1.516231924
si:dkeyp-116a7.2	ENSDARG00000097137	21.23	61.02333333	0.3479	-1.5232566	0.012702	1.896119128
si:dkeyp-51b9.3	ENSDARG00000092379	4.756666667	1.276666667	3.725849	1.897569	0.038103	1.419042898
si:dkeyp-52c3.5	ENSDARG00000038392	2.483333333	1.046666667	2.372611	1.2464759	0.013574	1.867302409
si:dkeyp-72e1.9	ENSDARG00000103743	1.09	3.103333333	0.351235	-1.5094905	0.007903	2.102185988
si:dkeyp-74f1.2	ENSDARG00000097512	2.313333333	4.3	0.537984	-0.8943635	0.013043	1.884635447
si:dkeyp-79b7.13	ENSDARG00000102619	7.086666667	10.42	0.680102	-0.5561762	0.033035	1.481020818
si:dkeyp-89c11.2	ENSDARG00000089159	3.946666667	2.096666667	1.882353	0.9125372	0.017149	1.765757246
si:dkeyp-93h6.4	ENSDARG00000096638	0.246666667	2.426666667	0.101648	-3.2983413	0.034322	1.464430676
si:rp71-36d5.4	ENSDARG00000012581	3.776666667	6.076666667	0.621503	-0.6861667	0.048892	1.310763942
si:zfos-128g4.2	ENSDARG00000098889	5.17	10.76666667	0.480186	-1.0583355	0.014183	1.84823237
si:zfos-411a11.3	ENSDARG00000099299	7.22	22.01333333	0.327983	-1.6083069	0.000455	3.342446603
SIKE1	ENSDARG00000102199	17.16	13.29333333	1.290873	0.3683466	0.033049	1.480842281
sipa1l1	ENSDARG00000020134	2.406666667	3.8	0.633333	-0.6589631	0.025336	1.596254552
six1b	ENSDARG00000026473	8.513333333	6.41	1.328133	0.4093998	0.045507	1.341920735
six3a	ENSDARG00000058008	29.42333333	48.23	0.610063	-0.7129701	0.020578	1.686604148
six3b	ENSDARG00000054879	37.98	65.6	0.578963	-0.7884559	0.016495	1.782658431
ska1	ENSDARG00000039354	7.24	5.536666667	1.307646	0.386972	0.047097	1.327002957
SLC16A1 (1 of 2)	ENSDARG00000016963	3	4.61	0.650759	-0.6198043	0.046921	1.328629514
slc16a3	ENSDARG00000045051	8.086666667	17.02666667	0.474941	-1.074179	0.019849	1.702254653
slc1a8a	ENSDARG00000104204	1.35	4.563333333	0.295836	-1.7571286	0.016912	1.771810806
slc24a2	ENSDARG00000042988	3.47	10.03333333	0.345847	-1.5317934	0.026668	1.574006191
slc25a12	ENSDARG00000102362	26.82	30.73333333	0.872668	-0.196495	0.047543	1.322914271
slc25a40	ENSDARG00000015856	3.48	4.823333333	0.721493	-0.4709432	0.043363	1.362876029
slc25a43	ENSDARG00000102048	7.713333333	5.323333333	1.448967	0.5350246	0.048943	1.310312257
slc25a44b	ENSDARG00000035905	4.696666667	6.933333333	0.677404	-0.5619119	0.007087	2.149519031
slc27a1a	ENSDARG00000006240	20.45666667	16.80666667	1.217176	0.2835375	0.015937	1.797591693
slc2a3a	ENSDARG00000013295	17.41333333	30.36333333	0.573499	-0.8021378	0.02051	1.688033464
slc30a9	ENSDARG00000057272	15.54333333	20.47333333	0.759199	-0.3974501	0.003357	2.474085007
slc35a4	ENSDARG00000062379	3.876666667	7.146666667	0.542444	-0.8824538	0.035809	1.446006143
slc35e1	ENSDARG00000011945	7.94	11.24	0.706406	-0.5014311	0.030264	1.519073887
slc35f2	ENSDARG00000069745	5.1	2.766666667	1.843373	0.8823484	0.003213	2.493131307
slc37a4b	ENSDARG00000077180	2.42	11.96	0.202341	-2.3051384	0.002651	2.576596174
slc38a2	ENSDARG00000045886	14.55	28.87333333	0.503925	-0.9887185	0.006781	2.168710872
slc38a3b	ENSDARG00000091061	7.396666667	4.623333333	1.599856	0.6779419	0.005455	2.263240169
slc38a7	ENSDARG00000012002	7.59	10	0.759	-0.3978282	0.013163	1.880630012
slc4a8	ENSDARG00000015531	1.846666667	4.38	0.421613	-1.2460074	0.040525	1.392280928
slc6a17	ENSDARG00000068787	2.636666667	16.03	0.164483	-2.6039873	0.011446	1.941363162
slc7a8b	ENSDARG00000075831	17.53333333	25.4	0.690289	-0.5347282	0.00463	2.334431759
slc9a7	ENSDARG00000076754	7.856666667	12.59333333	0.623875	-0.6806709	0.027694	1.557616544
slc9a8	ENSDARG00000020699	2.413333333	6.09	0.396278	-1.335415	0.001559	2.80706025

slco5a1	ENSDARG00000071685	0.936666667	4.536666667	0.206466	-2.276025	0.004969	2.303700325
slirp	ENSDARG00000097753	131.73	64.17666667	2.052615	1.0374632	0.012025	1.919904991
slitrk2	ENSDARG00000006636	3.373333333	7.326666667	0.460419	-1.1189821	0.014541	1.837399494
slmapb	ENSDARG00000020764	6.756666667	12.41	0.544453	-0.8771195	0.039223	1.406461036
smim12	ENSDARG00000070358	17.80666667	20.95333333	0.849825	-0.2347623	0.026763	1.572472217
smim7	ENSDARG00000074848	14.30666667	11.54	1.239746	0.3100444	0.008534	2.068855854
smox	ENSDARG00000036967	40.69333333	54.51666667	0.746438	-0.4219049	0.004783	2.320283363
smtnb	ENSDARG00000059442	5.823333333	11.78	0.494341	-1.0164224	0.011752	1.929898862
smtnl1	ENSDARG00000041257	29.39333333	12.83666667	2.289795	1.1952184	0.042475	1.371869753
smurf1	ENSDARG00000016086	4.156666667	6.133333333	0.677717	-0.5612443	0.021844	1.660659523
smx5	ENSDARG00000070867	155.5966667	92.18	1.687966	0.7552855	0.038644	1.412913054
snap25b	ENSDARG00000058117	76.50333333	206.12	0.371159	-1.42989	0.002084	2.681101299
snap29	ENSDARG00000038518	7.61	9.273333333	0.820633	-0.2851916	0.019454	1.710982572
snoU85	ENSDARG00000104320	0.446666667	3.33	0.134134	-2.8982517	0.009244	2.034162842
snrnp48	ENSDARG00000039989	9.66	7.79	1.240051	0.3103999	0.004289	2.367687032
snrpd2	ENSDARG00000040440	332.7233333	215.0166667	1.54743	0.6298746	0.0018	2.744616554
snrpg	ENSDARG00000099667	374.1566667	248.9133333	1.50316	0.587999	0.023054	1.637252584
snw1	ENSDARG00000091563	94.49	58.68666667	1.610076	0.6871289	0.045615	1.34089181
snx25	ENSDARG00000099545	8.233333333	13.17333333	0.625	-0.6780719	0.018128	1.741645734
snx27b	ENSDARG00000016977	5.073333333	9.036666667	0.561416	-0.8328568	0.046737	1.330339326
snx4	ENSDARG00000022659	15.37666667	17.31	0.888311	-0.1708629	0.041655	1.380333606
snx8b	ENSDARG00000078057	3.086666667	7.733333333	0.399138	-1.3250407	0.02285	1.641120924
socs6b	ENSDARG00000015863	5.52	8.596666667	0.642109	-0.6391091	0.028925	1.53873374
SOGA1 (2 of 2)	ENSDARG00000078244	0.336666667	2.603333333	0.129321	-2.9509673	0.000971	3.012640829
sort1b	ENSDARG00000056252	12.68666667	8.913333333	1.423336	0.5092761	0.031093	1.507342006
sos2	ENSDARG00000060506	3.273333333	5.593333333	0.585221	-0.7729478	0.025808	1.588246681
SOWAHA (2 of 2)	ENSDARG00000079125	3.313333333	1.856666667	1.78456	0.8355685	0.018338	1.736642199
sox13	ENSDARG00000030297	10.62	6.623333333	1.603422	0.6811544	0.031852	1.496869348
spata20	ENSDARG00000013880	2.09	2.95	0.708475	-0.497212	0.040198	1.395798617
sphk2	ENSDARG00000069893	4.37	5.72	0.763986	-0.3883819	0.015218	1.817645135
SPINK2	ENSDARG00000102076	5.533333333	1.94	2.852234	1.5120922	0.016105	1.793042473
spire1a	ENSDARG00000035868	5.193333333	6.84	0.759259	-0.3973355	0.010199	1.991448371
spock3	ENSDARG00000070266	52.67666667	79.19333333	0.665165	-0.5882149	0.01724	1.76346638
spop	ENSDARG00000100519	18.7	28.45666667	0.65714	-0.6057284	0.002118	2.67413365
spred1	ENSDARG00000041449	14.50666667	22.61666667	0.641415	-0.6406703	0.020857	1.680739314
spred3	ENSDARG00000040358	6.773333333	13.44	0.503968	-0.9885952	0.02928	1.53342158
spryd4	ENSDARG00000023309	38.11333333	23.19333333	1.643288	0.7165856	0.040338	1.394289771
spryd7b	ENSDARG00000069485	8.056666667	13.47666667	0.597823	-0.7422087	0.044262	1.353966451
sptssa	ENSDARG00000071203	21.06333333	16.12	1.306658	0.385882	0.030271	1.518966838
spty2d1	ENSDARG00000033889	29.47	19.15666667	1.538368	0.6214005	0.01082	1.965761628
srpk1a	ENSDARG00000073997	9.256666667	18.22333333	0.507957	-0.9772222	0.003617	2.441694976
srpk2	ENSDARG00000029368	9.44	16.14333333	0.584762	-0.7740797	0.005212	2.282984124
SRR (1 of 2)	ENSDARG00000096045	12.75	5.466666667	2.332317	1.2217639	0.007644	2.116680504
srsf1b	ENSDARG00000017843	20.9	31.47666667	0.663984	-0.5907798	0.025192	1.598740004
ss18l2	ENSDARG00000101085	49.09666667	31.78	1.544892	0.627506	0.042028	1.37645893
ssr4	ENSDARG00000019444	328.01	174.8333333	1.87613	0.9077595	0.003101	2.50847592
ssu72	ENSDARG00000031216	38.91333333	55.71333333	0.698456	-0.5177581	0.035717	1.447129716
st3gal2	ENSDARG00000102369	8.463333333	12.23	0.692014	-0.5311265	0.037921	1.421118116
st3gal3a	ENSDARG00000015374	3.33	7.15	0.465734	-1.1024211	0.021939	1.658791818
stag2a	ENSDARG00000011783	6.55	8.026666667	0.81603	-0.2933061	0.048306	1.315998453
stag2b	ENSDARG00000053668	15.41333333	23.88333333	0.645359	-0.6318253	0.006263	2.203199407
stard3nl	ENSDARG00000045421	17.49333333	12.60666667	1.387626	0.4726184	0.036425	1.438602341
stk25b	ENSDARG00000039022	19.23	34.49666667	0.557445	-0.8430982	0.026526	1.57632807
stmn1a	ENSDARG00000004169	219.35	126.16	1.738665	0.7979801	0.013165	1.880574301

STRADB	ENSDARG00000088225	2.343333333	6.216666667	0.376944	-1.407579	0.014797	1.829817316
stx12l	ENSDARG00000044605	7.376666667	13.26666667	0.55603	-0.846765	0.045986	1.337377616
stx16	ENSDARG00000003307	6.356666667	8.916666667	0.712897	-0.488234	0.00178	2.749582848
stx4	ENSDARG00000052518	27.15666667	16.44	1.651865	0.7240961	0.028642	1.542991378
stx8	ENSDARG00000103173	4.536666667	9.003333333	0.503887	-0.9888266	0.000627	3.202880771
stxbp1a	ENSDARG00000001994	83.98666667	169.8066667	0.494602	-1.0156609	0.020672	1.684626585
stxbp1b	ENSDARG00000056036	20.98	53.46	0.392443	-1.3494452	0.005014	2.299819066
stxbp6l	ENSDARG00000028354	0.743333333	2.25	0.33037	-1.5978438	0.001035	2.984981771
suco	ENSDARG00000016532	5.426666667	8.176666667	0.663677	-0.5914465	0.02801	1.55267931
supt6h	ENSDARG00000006524	11.17	17	0.657059	-0.6059056	0.018486	1.733146663
SUSD3 (2 of 2)	ENSDARG00000069607	0.98	4.893333333	0.200272	-2.3199639	0.01316	1.880749229
susd4	ENSDARG00000026335	3.843333333	5.313333333	0.723338	-0.4672591	0.012283	1.910697446
susd6	ENSDARG00000030116	9.15	12.91333333	0.70857	-0.4970178	0.02391	1.621412521
swap70b	ENSDARG00000057286	6.213333333	4.91	1.265445	0.3396444	0.008755	2.057761279
sybl1	ENSDARG00000030775	5.96	8.81	0.676504	-0.5638297	0.025282	1.597188354
syk	ENSDARG00000008186	1.583333333	2.88	0.549769	-0.8631038	0.038595	1.413472067
syne1a	ENSDARG00000009499	2.716666667	4.026666667	0.674669	-0.5677485	0.022632	1.645283597
syne1b	ENSDARG00000063068	2.11	3.213333333	0.656639	-0.6068276	0.008028	2.095390184
SYNPR (1 of 2)	ENSDARG00000037587	2.666666667	5.86	0.455063	-1.1358632	0.014315	1.844209631
syt10	ENSDARG00000045750	1.68	2.803333333	0.599287	-0.7386821	0.035175	1.453771682
syt5a	ENSDARG00000037941	5.11	17.15333333	0.297901	-1.7470938	0.015961	1.796949877
syt5b	ENSDARG00000011640	65.43666667	128.6133333	0.508786	-0.9748691	0.009036	2.044044813
szrd1	ENSDARG00000056338	19.14666667	27.65	0.692465	-0.5301862	0.0223	1.651686983
tab3	ENSDARG00000062063	7.663333333	13.70666667	0.559095	-0.8388338	0.020078	1.697285839
tacc1	ENSDARG00000073753	5.556666667	7.383333333	0.752596	-0.4100526	0.047948	1.319225343
TACC2	ENSDARG00000062790	17.30333333	24.50666667	0.706066	-0.5021243	0.001651	2.782183064
taf1	ENSDARG00000035330	4.663333333	6.88	0.67781	-0.561047	0.021872	1.660110201
taf10	ENSDARG00000068641	107.6533333	73.65666667	1.461556	0.547505	0.022504	1.64773389
TAF9	ENSDARG00000077870	29.27666667	20.72	1.412967	0.4987273	0.022371	1.650324051
tagln	ENSDARG00000045408	2.316666667	6.366666667	0.363874	-1.4584878	0.04812	1.317678206
TARS2	ENSDARG00000075385	7.996666667	14.35666667	0.557	-0.8442502	0.002676	2.57254159
tarsl2	ENSDARG00000092774	12.72	22.32	0.569892	-0.8112384	0.004885	2.311150426
tbc1d24	ENSDARG00000069339	11.32333333	20.74	0.545966	-0.8731172	0.035383	1.451206934
tbc1d25	ENSDARG00000075333	4.583333333	6.57	0.697615	-0.5194962	0.007923	2.101097194
TBC1D8B	ENSDARG00000062192	3.523333333	5.266666667	0.668987	-0.5799492	0.005993	2.222374378
tbcb	ENSDARG00000068404	60.73	48.73333333	1.24617	0.3175005	0.020555	1.687088393
tbck	ENSDARG00000013667	5.15	6.5	0.792308	-0.3358673	0.035215	1.453268975
tbk1	ENSDARG00000103095	5.326666667	9.416666667	0.565664	-0.8219835	0.0499	1.301899138
tbx2a	ENSDARG00000018025	15.22	19.97666667	0.761889	-0.3923475	0.044172	1.354855238
tcf7l1a	ENSDARG00000038159	6.806666667	8.706666667	0.781776	-0.355172	0.042643	1.370155409
tdp2b	ENSDARG00000035954	7.85	4.493333333	1.747033	0.8049066	0.012841	1.891414116
TEAD4		3.083333333	2.24	1.376488	0.4609921	0.049961	1.301366809
tefa	ENSDARG00000039117	21.59	36.09	0.598227	-0.7412359	0.031427	1.5026992
ten1	ENSDARG00000097346	13.06	7.906666667	1.651771	0.7240134	0.028716	1.541878563
tex2	ENSDARG00000078186	1.453333333	3.526666667	0.412098	-1.2789396	0.003316	2.479420398
tex9	ENSDARG00000010586	2.36	0.843333333	2.798419	1.484612	0.049471	1.305650752
tfap2b	ENSDARG00000012667	46.68333333	65.65	0.711094	-0.4918874	0.033165	1.479325107
tfg	ENSDARG00000003641	34.81333333	53.12333333	0.65533	-0.6097057	0.030694	1.512941932
tfip11	ENSDARG00000056932	12.74333333	9.103333333	1.399854	0.4852759	0.007522	2.123652564
tfpt	ENSDARG00000089572	9.726666667	13.65666667	0.712228	-0.489588	0.037109	1.430522784
thoc7	ENSDARG00000015394	64.68	43.24	1.495837	0.5809532	0.041276	1.384298993
tiam1a	ENSDARG00000078430	8.083333333	11.37666667	0.710519	-0.4930557	0.048662	1.312809057
timd4	ENSDARG00000077257	2.346666667	1.063333333	2.206897	1.142019	0.041462	1.382348039
timm10	ENSDARG00000069116	48.86666667	65.73666667	0.74337	-0.4278476	0.036022	1.443427038

timm13	ENSDARG00000058297	57.90333333	74.39666667	0.778305	-0.3615916	0.028475	1.545539033
tkfc	ENSDARG00000062085	1.863333333	4.42	0.421569	-1.2461606	0.031794	1.497651787
tkta	ENSDARG00000029689	15.21333333	26.92	0.565131	-0.8233421	0.00981	2.008338675
tma16	ENSDARG00000008068	9.096666667	6.383333333	1.425065	0.511028	0.029062	1.536667105
TMEM102	ENSDARG00000087162	1.946666667	3.646666667	0.533821	-0.9055725	0.00021	3.67869294
tmem136b	ENSDARG00000035163	5.44	11.64	0.467354	-1.0974125	0.006977	2.156309763
tmem141	ENSDARG00000101963	20.00666667	9.016666667	2.218854	1.1498147	0.010151	1.993479274
tmem178	ENSDARG00000020758	5.826666667	13.65333333	0.426758	-1.2285105	0.019762	1.704175165
tmem237a	ENSDARG00000041735	7.716666667	26.93	0.286545	-1.8031645	0.044489	1.351750651
tmem240b	ENSDARG00000090145	5.15	11.84333333	0.434844	-1.2014309	0.008191	2.086682297
tmem260	ENSDARG00000076362	2.38	3.386666667	0.702756	-0.5089044	0.024747	1.606482777
tmem59l	ENSDARG00000003655	85.36333333	108.2833333	0.788333	-0.3431228	0.047161	1.326412935
tmem60	ENSDARG00000021589	6.433333333	11.31333333	0.568651	-0.8143857	0.042016	1.37658187
tmem63c	ENSDARG00000004158	3.723333333	5.393333333	0.690358	-0.5345824	0.043938	1.357155219
tmem9b	ENSDARG00000025693	37.84333333	50.82333333	0.744605	-0.4254518	0.007248	2.139811073
tmsb4x	ENSDARG00000077777	6014.103333	4882.39	1.231795	0.3007621	0.008916	2.049820971
TNFRSF14		4.853333333	0.01	485.3333	8.9228321	0.002373	2.624712182
tnfrsf19	ENSDARG00000041869	6.513333333	4.246666667	1.533752	0.6170652	0.023379	1.63118063
tnnt3a	ENSDARG00000030270	412.0066667	193.98	2.123965	1.0867598	0.001083	2.96556589
tnw	ENSDARG00000024829	68.65	37.04	1.853402	0.8901756	0.045775	1.339370807
tollip	ENSDARG00000098486	15.39	20.76333333	0.74121	-0.4320448	0.03645	1.43830799
tomm40l	ENSDARG00000036721	50.07333333	31.97666667	1.565933	0.6470229	0.021705	1.663430999
tp53bp1	ENSDARG00000079000	6.543333333	10.21666667	0.640457	-0.6428269	0.008958	2.047784485
tp53bp2a	ENSDARG00000009136	10.61	7.39	1.435724	0.5217784	0.036899	1.432984182
tpbga	ENSDARG00000099609	3.71	1.503333333	2.467849	1.3032543	0.009398	2.02698015
tpd52l2a	ENSDARG00000027154	13.90666667	30.36	0.458059	-1.1263951	0.003652	2.437418373
tpk1	ENSDARG00000038040	1.66	3.543333333	0.468485	-1.0939239	0.046365	1.33381311
tra2b	ENSDARG00000002168	24.89333333	38.28333333	0.650239	-0.620957	0.005403	2.26736797
trak2	ENSDARG00000102471	3.48	6.49	0.53621	-0.8991312	0.009065	2.042623002
tram1	ENSDARG00000019137	41.79	28.1	1.487189	0.5725876	0.002368	2.625614068
trappc13	ENSDARG00000033768	8.67	12.87666667	0.673311	-0.5706553	0.038062	1.419512013
trappc3	ENSDARG00000045364	59.36333333	71.38666667	0.831575	-0.2660825	0.024697	1.607354617
trib2	ENSDARG00000068179	7.173333333	12.72333333	0.563794	-0.8267611	0.009548	2.020082279
TRIL	ENSDARG00000100791	3.966666667	2.246666667	1.765579	0.8201411	0.02204	1.656795931
trim13	ENSDARG00000010010	10.96666667	17.08333333	0.641951	-0.6394644	0.025989	1.585204094
trim3b	ENSDARG00000005397	11.77	18.35666667	0.641184	-0.6411898	0.044022	1.356331818
trim8a	ENSDARG00000090512	9.613333333	12.47333333	0.770711	-0.3757384	0.006202	2.207449374
trmt10c	ENSDARG00000041575	10.85666667	6.55	1.657506	0.7290144	0.034339	1.464218429
trmt61b	ENSDARG00000097465	4.663333333	3.496666667	1.333651	0.4153813	0.033027	1.481128727
trnau1apa	ENSDARG00000101323	10.29333333	5.55	1.854655	0.8911506	0.007672	2.115094237
trpm7	ENSDARG00000036232	7.61	12.58	0.604928	-0.7251636	0.009834	2.007251966
try	ENSDARG00000042993	1.32	4.046666667	0.326194	-1.6161961	0.036891	1.433082589
tsc22d1	ENSDARG00000038306	73.96	100.7633333	0.733997	-0.4461536	0.011095	1.95486322
TSTD1	ENSDARG00000071567	16.98	7.76	2.188144	1.1297079	0.024913	1.603579472
TSTD2	ENSDARG00000098660	0.873333333	2.1	0.415873	-1.265785	0.016254	1.789043192
TTBK2 (2 of 2)	ENSDARG00000043026	3.493333333	6.8	0.513725	-0.9609304	0.047022	1.327698591
ttc13	ENSDARG00000063242	7.613333333	11.34666667	0.670975	-0.5756684	0.040234	1.395408276
ttc17	ENSDARG00000044812	2.336666667	3.936666667	0.593565	-0.7525226	0.001666	2.778325285
ttc3	ENSDARG00000086075	3.54	5.843333333	0.605819	-0.7230422	0.047039	1.327543846
ttc32	ENSDARG00000069241	41.43333333	26.54	1.561166	0.6426235	0.0463	1.334423022
ttc7a	ENSDARG00000074760	1.033333333	2.636666667	0.391909	-1.3514095	0.018858	1.724502759
ttc7b	ENSDARG00000061207	6.64	10.05666667	0.660259	-0.598897	0.021191	1.6738491
ttc8	ENSDARG00000099481	0.656666667	3.023333333	0.2172	-2.2029069	0.035102	1.454673129
tti2	ENSDARG00000073773	2.83	1.38	2.050725	1.0361338	0.005983	2.223058148

tuba8l3	ENSDARG00000070155	109.46	81.73666667	1.339179	0.4213484	0.010112	1.995163713
TULP1 (3 of 3)	ENSDARG00000100478	0.896666667	12.37	0.072487	-3.7861299	0.022332	1.651064375
tulp1a	ENSDARG00000075295	45.45	95.21	0.477366	-1.0668328	0.036559	1.437010326
TULP2	ENSDARG00000062902	10.64666667	18.87333333	0.564112	-0.8259474	0.022534	1.647154512
tulp4b	ENSDARG00000063056	4.083333333	7.32	0.557832	-0.8420963	0.045371	1.343224705
txndc12	ENSDARG00000038980	48.19666667	42.05666667	1.145994	0.1965989	0.006097	2.214876514
U3	ENSDARG00000101840	30.06666667	46.28	0.649669	-0.6222239	0.001334	2.874868389
uba3	ENSDARG00000057987	22.48	28.15666667	0.79839	-0.3248345	0.007207	2.14222317
ubap2l	ENSDARG00000063219	7.933333333	13.33666667	0.594851	-0.7493991	0.029017	1.537351135
ube2a	ENSDARG00000098466	46.65	74.82333333	0.623469	-0.6816112	0.003135	2.503693291
ube2d3	ENSDARG00000038473	39.06333333	60.40333333	0.646708	-0.6288131	0.02367	1.625807454
ube2d4	ENSDARG00000015057	4.74	7.403333333	0.640252	-0.6432879	0.008248	2.083630433
ube2s	ENSDARG00000031775	28.33666667	44.26	0.640232	-0.6433334	0.025803	1.588335614
ube2t	ENSDARG00000063285	18.10333333	10.03333333	1.804319	0.8514544	0.020391	1.690562073
ube2v1	ENSDARG00000041875	73.06333333	91.15666667	0.801514	-0.3192006	0.00804	2.094752314
ube3a	ENSDARG00000055737	12.20333333	16.27333333	0.749898	-0.4152345	0.031308	1.504350764
ubl3a	ENSDARG00000099532	26.19	39.9	0.656391	-0.6073727	0.028329	1.547775444
ubl3b	ENSDARG00000021556	23.76	41.57	0.571566	-0.8070079	0.02169	1.66374882
UBL4A	ENSDARG00000007359	46.24333333	30.31333333	1.525511	0.6092929	0.002345	2.629783594
ubxn7	ENSDARG00000009436	9.646666667	13.76	0.701066	-0.512378	0.045621	1.340833279
uchl3	ENSDARG00000030177	89.11333333	64.01666667	1.392033	0.4771937	0.047999	1.318767634
uchl5	ENSDARG00000103404	62.79	46.55333333	1.348776	0.4316503	0.007277	2.13801785
uckl1b	ENSDARG00000078973	2.666666667	4.503333333	0.592154	-0.7559558	0.009638	2.016032129
uggt2	ENSDARG00000062089	10.38	7.163333333	1.449046	0.5351035	0.009023	2.044652077
UIMC1	ENSDARG00000091187	8.416666667	12.21	0.689326	-0.5367423	0.017423	1.758871149
ulk2	ENSDARG00000097205	5.366666667	10.5	0.511111	-0.9682911	0.018207	1.739764886
unc119b	ENSDARG00000044362	48.74666667	67.28666667	0.724462	-0.4650171	0.021249	1.67266194
UNC13B (2 of 2)	ENSDARG00000099113	0.643333333	3.216666667	0.2	-2.3219281	0.012916	1.888883486
unga	ENSDARG00000042527	12.57666667	7.043333333	1.785613	0.8364193	0.008745	2.058231974
uo:ion006	ENSDARG00000034321	7.346666667	19.52333333	0.376302	-1.4100377	0.044553	1.351121522
upk1a	ENSDARG00000021866	0.85	2.006666667	0.423588	-1.2392662	0.02796	1.553461802
usf1	ENSDARG00000041689	50.66333333	36.13	1.402251	0.4877447	0.040944	1.387813494
usp12a	ENSDARG00000078109	12.69333333	20.74333333	0.611924	-0.7085768	0.007781	2.108982526
usp7	ENSDARG00000073710	19.6	24.44333333	0.801855	-0.3185874	0.016708	1.777067193
uxt	ENSDARG00000019339	13.81	5.443333333	2.537048	1.343151	0.00273	2.56377815
vamp1	ENSDARG00000031283	36.47333333	71.27666667	0.511715	-0.9665878	0.010089	1.996137004
vapal	ENSDARG00000115862	43.62333333	61.64	0.707711	-0.4987668	0.000313	3.504699789
vbp1	ENSDARG00000030241	57.44333333	80.17	0.716519	-0.480923	0.031532	1.501254018
vdac1	ENSDARG00000045132	165.5666667	257.0833333	0.644019	-0.6348238	0.029246	1.533932507
vegfaa	ENSDARG00000103542	8.366666667	5.573333333	1.501196	0.5861125	0.015098	1.821084491
vldlr	ENSDARG00000006257	18.81666667	29.78333333	0.631785	-0.6624941	0.043943	1.357113141
vps11	ENSDARG00000036338	5.596666667	8.633333333	0.648263	-0.6253499	0.002696	2.569234081
vps18	ENSDARG00000070433	12.10333333	9.103333333	1.32955	0.4109376	0.033705	1.472307352
vps35	ENSDARG00000008224	13.37666667	23.92666667	0.559069	-0.8389008	0.011225	1.94982053
waca	ENSDARG00000012577	21.48	26.39333333	0.813842	-0.2971796	0.047447	1.323790438
wbp2	ENSDARG00000056605	3.856666667	10.57	0.364869	-1.454549	0.012938	1.888142615
wdr11	ENSDARG00000075245	6.553333333	9.433333333	0.6947	-0.5255387	0.001403	2.852922082
wdr12	ENSDARG00000003287	16.40333333	11.97666667	1.369608	0.4537626	0.002415	2.617070539
wdr13	ENSDARG00000022974	14.28	24.72666667	0.577514	-0.7920718	0.00012	3.922356695
wdr18	ENSDARG00000041113	20.65	13.11666667	1.574333	0.6547406	0.040394	1.393683598
wdr20a	ENSDARG00000079220	2.61	4.346666667	0.60046	-0.7358597	0.001107	2.955685499
wdr37	ENSDARG00000074611	14.15	21.16666667	0.668504	-0.580992	0.025238	1.597937429
wdr45	ENSDARG00000090219	7.95	9.213333333	0.86288	-0.2127683	0.021396	1.669676697
wdr82	ENSDARG00000014083	43.51	34.51333333	1.260672	0.3341932	0.028981	1.537883621

wls	ENSDARG00000009534	56.19333333	44.09666667	1.274322	0.3497294	0.032196	1.492191469
wu:fb92b05	ENSDARG00000044695	7.643333333	4.33	1.765204	0.8198349	0.005534	2.25692776
wu:fc46h12	ENSDARG00000104814	785.32	383.6866667	2.046774	1.033352	0.026054	1.584124818
xkr7	ENSDARG00000026333	2.686666667	5.293333333	0.507557	-0.9783592	0.036258	1.4405987
ybx1	ENSDARG00000004757	1301.933333	961.33	1.354304	0.4375519	0.049494	1.305445514
ypel3	ENSDARG00000055510	18.15333333	22.11	0.821046	-0.2844645	0.010541	1.977112015
ythdc1	ENSDARG00000051953	7.99	10.17333333	0.785387	-0.3485251	0.017471	1.757683182
ythdf1	ENSDARG00000016447	17.74	23.73	0.747577	-0.4197061	0.021015	1.677461879
zbtb11	ENSDARG00000017886	10.16	12.38	0.820679	-0.2851109	0.045286	1.34403919
zc2hc1a	ENSDARG00000027915	20.79666667	34.94666667	0.595097	-0.7488025	0.038003	1.420182581
zcchc10	ENSDARG00000091092	20.72333333	13.80666667	1.500966	0.585891	0.012907	1.889175744
zcchc24	ENSDARG00000078026	8.643333333	6.286666667	1.374867	0.4592925	0.022836	1.641378233
zdhhc16a	ENSDARG00000007808	8.28	16.76333333	0.493935	-1.0176064	0.000503	3.298245825
zfand5b	ENSDARG00000002271	36.49666667	52.07	0.700915	-0.5126877	0.0264	1.578395397
ZFOS-932H1.3	ENSDARG00000098311	2.616666667	4.043333333	0.647156	-0.627815	0.012053	1.918914185
ZFP41	ENSDARG00000101105	3.1	2.016666667	1.53719	0.6202956	0.02075	1.682976024
zfyve9a	ENSDARG00000101903	1.96	3.623333333	0.540938	-0.8864639	0.034942	1.456648208
zgc:101731	ENSDARG00000040965	3.063333333	1.87	1.638146	0.7120641	0.002849	2.545360492
zgc:109744	ENSDARG00000070800	4.66	2.923333333	1.594071	0.6727156	0.001736	2.760349915
zgc:109965	ENSDARG00000012126	12.92333333	31.37	0.411965	-1.2794073	0.043062	1.365905902
zgc:110540	ENSDARG00000054929	60.54666667	28.85	2.098671	1.0694762	0.009891	2.004774818
zgc:112970	ENSDARG00000098332	0.17	2.04	0.083333	-3.5849625	0.034323	1.464413231
zgc:113098	ENSDARG00000076442	1.053333333	2.783333333	0.378443	-1.4018516	0.004325	2.364041889
zgc:113314	ENSDARG00000069453	5.51	1	5.51	2.4620523	0.007567	2.121104967
zgc:123105	ENSDARG00000003127	23.88	36.09	0.661679	-0.5957963	0.018446	1.734087821
zgc:136971	ENSDARG00000055970	1.776666667	3.266666667	0.543878	-0.8786462	0.042399	1.372646722
zgc:152863	ENSDARG00000099969	3.106666667	4.893333333	0.634877	-0.6554501	0.047739	1.321125924
zgc:153426	ENSDARG00000104436	161.3733333	313.7933333	0.514266	-0.9594125	0.005907	2.228637623
zgc:153675	ENSDARG00000071555	35.70666667	16.42	2.174584	1.1207393	0.049197	1.308058515
zgc:154093	ENSDARG00000055897	3.243333333	8.356666667	0.388113	-1.3654503	0.010859	1.964211769
zgc:158254	ENSDARG00000071233	2.843333333	7.31	0.388965	-1.3622882	0.002281	2.641785883
zgc:158263	ENSDARG00000037267	4.34	1.11	3.90991	1.9671354	0.026245	1.580949451
zgc:158463	ENSDARG00000089382	780.4566667	4096.353333	0.190525	-2.3919497	0.003277	2.484459349
zgc:158785	ENSDARG00000062959	1.15	2.553333333	0.450392	-1.150748	0.036696	1.435384785
zgc:162356	ENSDARG00000042620	37.17	18.91666667	1.964934	0.9744808	0.007293	2.137112867
zgc:162509	ENSDARG00000070604	2.446666667	0.2	12.23333	3.6127457	0.003477	2.458748461
zgc:163143	ENSDARG00000076437	2.11	1.283333333	1.644156	0.7173471	0.009896	2.004542819
zgc:171242	ENSDARG00000078551	0.14	3.636666667	0.038497	-4.699118	0.002307	2.636890457
zgc:173994	ENSDARG00000029473	1.333333333	5.17	0.257898	-1.9551268	0.00017	3.770502191
zgc:174263	ENSDARG00000097244	7.64	5.933333333	1.28764	0.3647298	0.049323	1.306952441
zgc:174648	ENSDARG00000100961	6.296666667	3.703333333	1.70027	0.7657639	0.022164	1.654344793
zgc:174906	ENSDARG00000075314	31.1	18.65333333	1.667262	0.7374811	0.007812	2.10725514
zgc:193593	ENSDARG00000078130	3.546666667	18.72333333	0.189425	-2.4003017	0.007473	2.126532588
zgc:194207	ENSDARG00000097799	19.06	6.533333333	2.917347	1.544657	0.008857	2.052726871
zgc:63587	ENSDARG00000017140	41.23333333	27.42	1.503769	0.5885825	0.023672	1.62575714
zgc:64189	ENSDARG00000033501	0.883333333	3.58	0.246741	-2.0189297	0.036185	1.441471246
zgc:64201	ENSDARG00000101636	0.763333333	2.26	0.337758	-1.5659377	0.028193	1.549856141
zgc:66475	ENSDARG00000099519	9.446666667	6.516666667	1.449616	0.5356712	0.029599	1.528726474
zgc:77158	ENSDARG00000013784	3.81	2.133333333	1.785938	0.8366816	0.041115	1.386000248
zgc:77816	ENSDARG00000011376	3.94	2.47	1.595142	0.6736846	0.016291	1.788043101
zgc:91860	ENSDARG00000041599	16.06	28.47333333	0.564037	-0.8261395	0.01194	1.922983484
zgc:92107	ENSDARG00000044874	7.473333333	5.62	1.329775	0.4111817	0.042008	1.37666731
zgc:92664	ENSDARG00000015161	63.64333333	38.28666667	1.662285	0.7331673	0.008338	2.07894959
zgpat	ENSDARG00000027403	15.55	10.86	1.43186	0.5178905	0.043201	1.36450479

zic4	ENSDARG00000031307	8.09	10.49666667	0.770721	-0.3757196	0.018734	1.727379706
znf330	ENSDARG00000020494	30.26333333	20.62	1.467669	0.5535266	0.000727	3.138756388
znf410	ENSDARG00000008218	13.25333333	17.64333333	0.751181	-0.4127679	0.049304	1.307115252
znf414	ENSDARG00000079229	19.69666667	13.03333333	1.511253	0.5957454	0.000764	3.116671524
znf622	ENSDARG00000014692	18.8	12.8	1.46875	0.5545889	0.010515	1.978210362
ZNF638	ENSDARG00000104169	11.21333333	16.67333333	0.672531	-0.5723274	0.013118	1.882138575
ZNF784 (3 of 6)	ENSDARG00000092327	8.93	2.393333333	3.731198	1.8996388	0.046569	1.331899212
znf839	ENSDARG00000076988	8.896666667	13.77	0.646091	-0.6301918	0.001818	2.740472572
ZNF865 (1 of 2)		11.98333333	16.15	0.742002	-0.4305049	0.029424	1.531299567
ZNHIT6	ENSDARG00000095817	8.966666667	5.353333333	1.674969	0.7441343	0.049997	1.301060293
zwilch	ENSDARG00000057100	5.606666667	2.953333333	1.89842	0.9247991	0.027435	1.561697987
