# Supplementary material for: Hnrnpul1 controls transcription, splicing, and modulates skeletal and limb development in vivo
Source: G3 (Bethesda). 2022 Mar 23;12(5):jkac067. doi: 10.1093/g3journal/jkac067 (PMC9073674; doi:10.1093/g3journal/jkac067)
Supplement: jkac067_Supplementary_Table_S3 [file jkac067_supplementary_table_s3.pdf]

Blackwell et al., Table S3 IPA pathways

Symbol	Entrez Gene Name	Ensembl	Expr Fold	Expr Log	Expr p-val	Expected	Location	Type(s)	Entrez Gene ID for Human	Entrez Gene ID for Mouse	Entrez Gene ID for Rat
EIF1AX	eukaryotic translation initiation factor 1A X-linked	ENSDARG00000057912	1.233	0.303	1.09E-02		Cytoplasm	translation	1964	13664	317163
EIF3B	eukaryotic translation initiation factor 3 subunit B	ENSDARG00000059654	1.36	0.444	3.02E-03		Cytoplasm	translation	8662	27979	288516
EIF3K	eukaryotic translation initiation factor 3 subunit K	ENSDARG00000068289	1.758	0.814	2.15E-03		Cytoplasm	translation	27335	73830	292762
FAU	FAU ubiquitin like and ribosomal protein S30 fusion	ENSDARG00000043663	1.924	0.944	1.51E-02		Cytoplasm	other	2197	14109	29752|687780|100360647
RPL5	ribosomal protein L5	ENSDARG00000020197	1.388	0.473	8.39E-03	Up	Cytoplasm	other	6125	100503670	81763
RPL7	ribosomal protein L7	ENSDARG00000007320	1.564	0.645	1.64E-03	Up	Nucleus	transcriptic	6129	19989	297755
RPL9	ribosomal protein L9	ENSDARG00000037350	1.571	0.651	3.44E-03	Up	Nucleus	other	6133	20005	29257|100360449|100364457
RPL11	ribosomal protein L11	ENSDARG00000043509	1.756	0.812	2.32E-02	Up	Cytoplasm	other	6135	67025	362631
RPL12	ribosomal protein L12	ENSDARG00000006691	1.314	0.394	1.57E-04	Up	Nucleus	other	6136	269261	499782|102555453
RPL14	ribosomal protein L14	ENSDARG00000103433	1.241	0.312	1.78E-02	Up	Cytoplasm	other	9045	67115	65043
RPL23	ribosomal protein L23	ENSDARG00000053457	1.355	0.438	9.76E-04	Up	Cytoplasm	other	9349	65019	29282
RPL27	ribosomal protein L27	ENSDARG00000015128	1.409	0.494	3.69E-02	Up	Cytoplasm	other	6155	19942	64306
RPL30	ribosomal protein L30	ENSDARG00000035871	1.685	0.753	2.60E-03	Up	Cytoplasm	other	6156	19946	64640|100362027
RPL32	ribosomal protein L32	ENSDARG00000054818	1.454	0.54	1.97E-03	Up	Cytoplasm	other	6161	621697	
RPL37	ribosomal protein L37	ENSDARG00000034291	1.455	0.541	3.00E-02	Up	Cytoplasm	other	6167	67281	81770|100360781|100360841
RPL38	ribosomal protein L38	ENSDARG00000006413	1.219	0.285	2.36E-02	Up	Cytoplasm	other	6169	67671	
Rpl22l1	ribosomal protein L22 like 1	ENSDARG00000010244	1.904	0.929	4.50E-03	Up	Other	other		68028	361923
RPL36A	ribosomal protein L36a	ENSDARG00000058105	1.316	0.396	1.26E-02	Up	Cytoplasm	other	6173		100912182
RPS7	ribosomal protein S7	ENSDARG00000042566	1.522	0.606	1.85E-02		Cytoplasm	other	6201	20115	29258|100362830
RPS11	ribosomal protein S11	ENSDARG00000053058	1.554	0.636	5.86E-03		Cytoplasm	other	6205	27207	81774
RPS20	ribosomal protein S20	ENSDARG00000036044	1.371	0.455	1.00E-02		Cytoplasm	other	6224	67427	122772
RPS24	ribosomal protein S24	ENSDARG00000039347	1.721	0.784	6.90E-04		Cytoplasm	other	6229	20088	81776
RPS27	ribosomal protein S27	ENSDARG00000023298	2.131	1.092	1.41E-03		Cytoplasm	other	6232		94266
RPS15A	ribosomal protein S15a	ENSDARG00000010160	1.564	0.645	4.28E-03		Cytoplasm	other	6210	267019	117053

Blackwell et al., Table S3 IPA pathways

Symbol	Entrez Gene Name	Ensembl	Expr Fold Change	Expr Log Ratio	Expr p-value	Expected	Location	Type(s)	Entrez Gene ID for Human	Entrez Gene ID for Mouse	Entrez Gene ID for Rat
ANAPC11	anaphase promoting c	ENSDARG(	1.704	0.769	6.94E-03	Down	Cytoplasm	enzyme	51529	66156	498030
BIRC5	baculoviral IAP repeat	ENSDARG(	1.73	0.79	1.95E-02	Up	Cytoplasm	other	332	11799	64041
CDC20	cell division cycle 20	ENSDARG(	1.548	0.63	4.98E-02	Down	Nucleus	other	991	107995	64515
CENPK	centromere protein K	ENSDARG(	1.702	0.767	1.58E-02		Nucleus	other	64105	60411	294712
CCNB1	cyclin B1	ENSDARG(	1.509	0.593	2.82E-02	Up	Cytoplasm	kinase	891	268697|434175	25203
CDK1	cyclin dependent kina:	ENSDARG(	1.331	0.413	3.43E-02	Up	Nucleus	kinase	983	12534	54237
PPP1R14I	protein phosphatase 1	ENSDARG(	1.451	0.537	2.48E-02	Down	Cytoplasm	phosphata:	26472	18938	259225
PPP1R7	protein phosphatase 1	ENSDARG(	1.44	0.526	2.17E-02	Down	Nucleus	phosphata:	5510	66385	301618
SKA1	spindle and kinetocho	ENSDARG(	1.308	0.387	4.71E-02	Down	Nucleus	other	220134	66468	291441
ZWILCH	zwilch kinetochore prc	ENSDARG(	1.898	0.925	2.74E-02	Up	Nucleus	other	55055	68014	691493

Symbol	Entrez Gene Name	Ensembl	Expr Fold Change	Expr Log Ratio	Expr p-value	Expected	Location	Type(s)	Entrez Gene ID for Human	Entrez Gene ID for Mouse	Entrez Gene ID for Rat
ANAPC11	anaphase promoting c	ENSDARG	1.704	0.769	6.94E-03		Cytoplasm	enzyme	51529	66156	498030
CDC20	cell division cycle 20	ENSDARG	1.548	0.63	4.98E-02		Nucleus	other	991	107995	64515
DNAJB6	DnaJ heat shock prote	ENSDARG	1.344	0.426	8.23E-03		Nucleus	transcriptic	10049	23950	362293
DNAJB12	DnaJ heat shock prote	ENSDARG	1.306	0.385	2.26E-02		Cytoplasm	other	54788	56709	294513
DNAJC9	DnaJ heat shock prote	ENSDARG	1.534	0.617	4.48E-03		Nucleus	other	23234	108671	364240
DNAJC16	DnaJ heat shock prote	ENSDARG	1.607	0.684	4.39E-02		Other	other	23341	214063	362652
DNAJC19	DnaJ heat shock prote	ENSDARG	1.403	0.489	1.99E-02		Cytoplasm	other	131118	67713|100503724	
FZR1	fizzy and cell division c	ENSDARG	1.298	0.377	2.48E-02		Nucleus	kinase	51343	56371	314642
PSMA2	proteasome 20S subu	ENSDARG	1.416	0.502	2.93E-02		Cytoplasm	peptidase	5683	19166	29669
PSMC1	proteasome 26S subu	ENSDARG	1.477	0.563	5.65E-03		Nucleus	peptidase	5700	19179	117263
PSMC4	proteasome 26S subu	ENSDARG	1.365	0.449	3.05E-02		Nucleus	peptidase	5704	23996	117262
PSMD4	proteasome 26S subu	ENSDARG	1.512	0.597	1.55E-02		Cytoplasm	other	5710	19185	83499
PSMD7	proteasome 26S subu	ENSDARG	1.556	0.638	4.90E-02		Cytoplasm	other	5713	17463	307821
UCHL3	ubiquitin C-terminal hy	ENSDARG	1.392	0.477	4.80E-02		Cytoplasm	peptidase	7347	50933	498560
UCHL5	ubiquitin C-terminal hy	ENSDARG	1.349	0.432	7.28E-03		Cytoplasm	peptidase	51377	56207	360853
UBE2T	ubiquitin conjugating e	ENSDARG	1.804	0.851	2.04E-02		Nucleus	enzyme	29089	67196	360847

Symbol	Entrez Gene Name	Ensembl	Expr Fold Change	Expr Log Ratio	Expr p-value	Expected	Location	Type(s)	Entrez Gene ID for Human	Entrez Gene ID for Mouse	Entrez Gene ID for Rat
CCNB1	cyclin B1	ENSDARG(	1.509	0.593	2.82E-02		Cytoplasm	kinase	891	268697|434175	25203
CCNB2	cyclin B2	ENSDARG(	1.416	0.502	1.10E-02		Cytoplasm	other	9133	12442	363088
CDK1	cyclin dependent kina:	ENSDARG(	1.331	0.413	3.43E-02		Nucleus	kinase	983	12534	54237
CDK2	cyclin dependent kina:	ENSDARG(	1.502	0.586	2.63E-02		Nucleus	kinase	1017	12566	362817
CCNE2	cyclin E2	ENSDARG(	1.557	0.639	1.22E-02		Nucleus	other	9134	12448	362485

Symbol	Entrez Gene Name	Ensembl	Expr Fold Change	Expr Log Ratio	Expr p-value	Expected	Location	Type(s)	Entrez Gene ID for Human	Entrez Gene ID for Mouse	Entrez Gene ID for Rat
EIF1AX	eukaryotic translation	ENSDARG	1.233	0.303	1.09E-02		Cytoplasm	translation	1964	13664	317163
EIF3B	eukaryotic translation	ENSDARG	1.36	0.444	3.02E-03		Cytoplasm	translation	8662	27979	288516
EIF3K	eukaryotic translation	ENSDARG	1.758	0.814	2.15E-03		Cytoplasm	translation	27335	73830	292762
EIF4EBP1	eukaryotic translation	ENSDARG	1.396	0.481	1.13E-02		Cytoplasm	translation	1978	13685	116636
EIF4EBP2	eukaryotic translation	ENSDARG	1.355	0.438	1.97E-03		Cytoplasm	translation	1979	13688	361845
FAU	FAU ubiquitin like and	ENSDARG	1.924	0.944	1.51E-02		Cytoplasm	other	2197	14109	29752|687780|100360647
RPS7	ribosomal protein S7	ENSDARG	1.522	0.606	1.85E-02		Cytoplasm	other	6201	20115	29258|100362830
RPS11	ribosomal protein S11	ENSDARG	1.554	0.636	5.86E-03		Cytoplasm	other	6205	27207	81774
RPS20	ribosomal protein S20	ENSDARG	1.371	0.455	1.00E-02		Cytoplasm	other	6224	67427	122772
RPS24	ribosomal protein S24	ENSDARG	1.721	0.784	6.90E-04		Cytoplasm	other	6229	20088	81776
RPS27	ribosomal protein S27	ENSDARG	2.131	1.092	1.41E-03		Cytoplasm	other	6232		94266
RPS15A	ribosomal protein S15	ENSDARG	1.564	0.645	4.28E-03		Cytoplasm	other	6210	267019	117053
