# Supplementary material for: Hnrnpul1 controls transcription, splicing, and modulates skeletal and limb development in vivo
Source: G3 (Bethesda). 2022 Mar 23;12(5):jkac067. doi: 10.1093/g3journal/jkac067 (PMC9073674; doi:10.1093/g3journal/jkac067)
Supplement: jkac067_Supplementary_Material [file jkac067_supplementary_material.pdf]

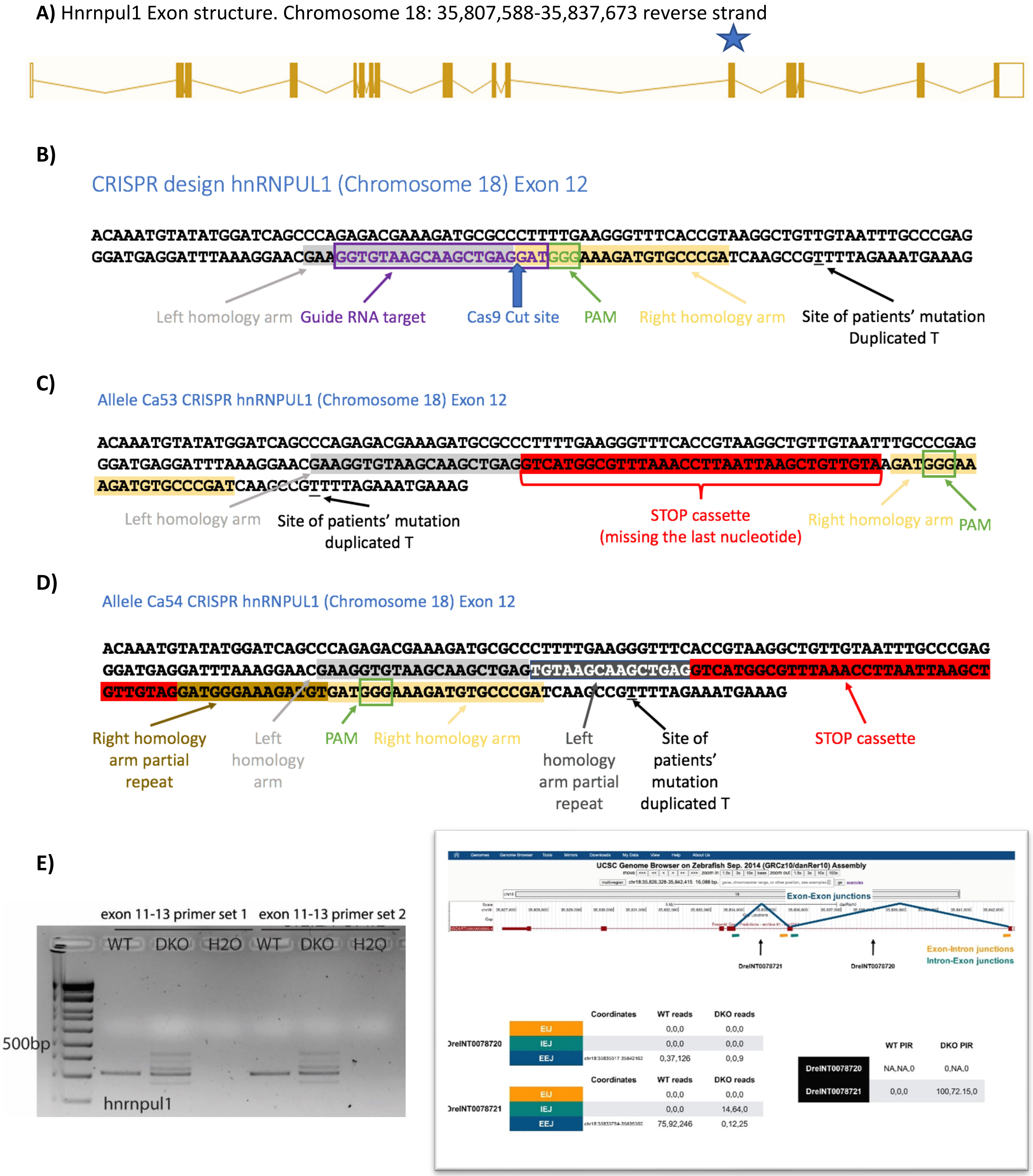

Figure S1 ***hnrnpul1*** **CRISPR design and mutation analysis:** A) Schematic from ENSEMBL of the hnrnpul1 exon-intron structure. The targeted exon is marked with a star. B) Details of the CRISPR design, homology directed repair oligo. C-D) details of mutations in alleles CA53 and CA54. E) Gel showing multiple splice products in mutants. Analysis showing low exon-exon splicing in mutants.

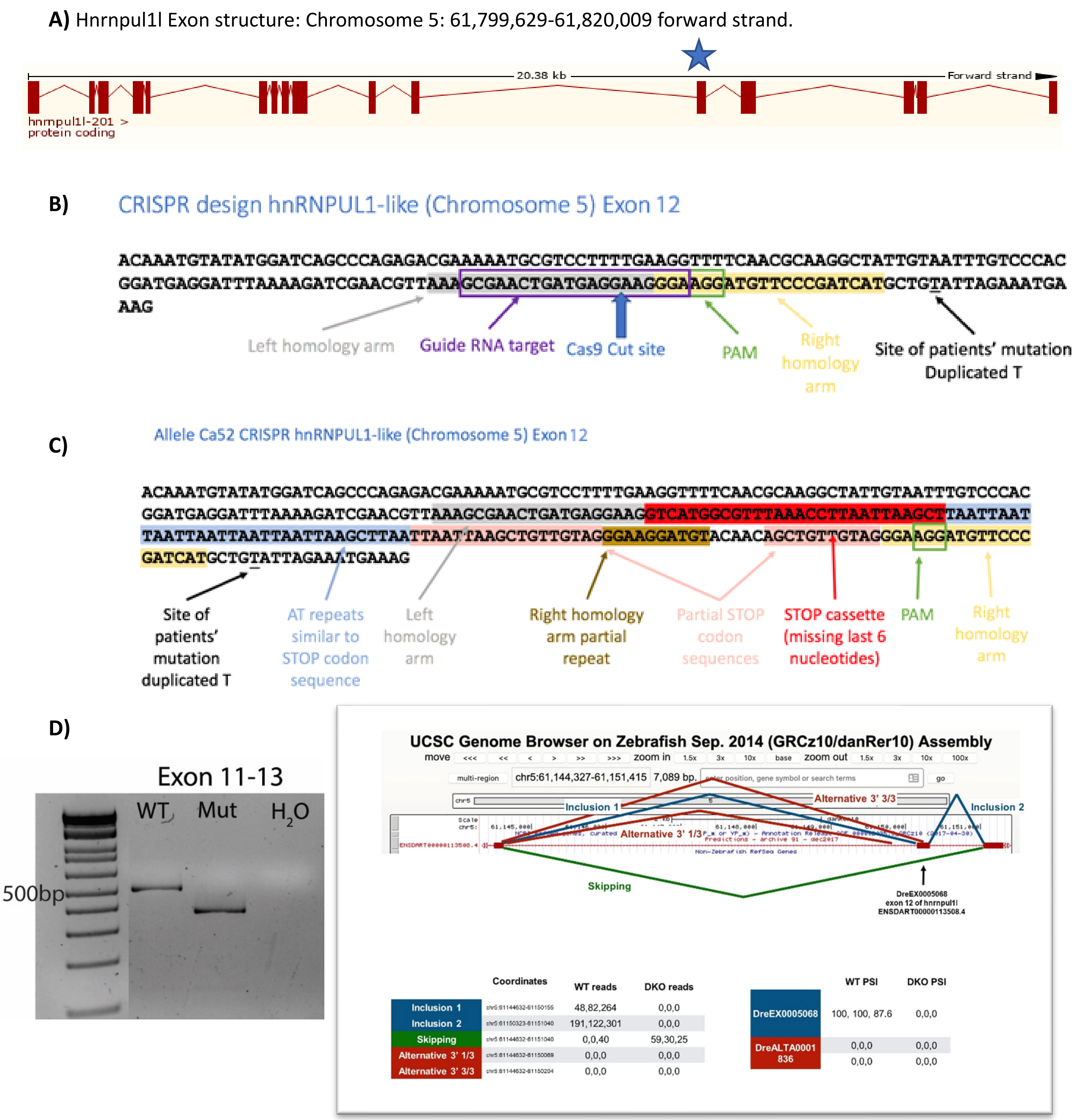

Figure S2 – *hnrnpul1l* CRISPR design and mutation analysis: A) Schematic from ENSEMBL of the hnrnpul1l exon-intron structure with the targeted exon marked with a star. B) Details of the CRISPR design and homology directed repair oligo. C) details of the mutation found in allele CA52. D) Gel showing exon 12 deletion (PCR between exon 11 and exon 13). E) RNAseq analysis of exon skipping in wildtype (no occurrences) and in double mutants (all detected transcripts, 59, 30 and 25 occurrences in 3 replicates respectively).

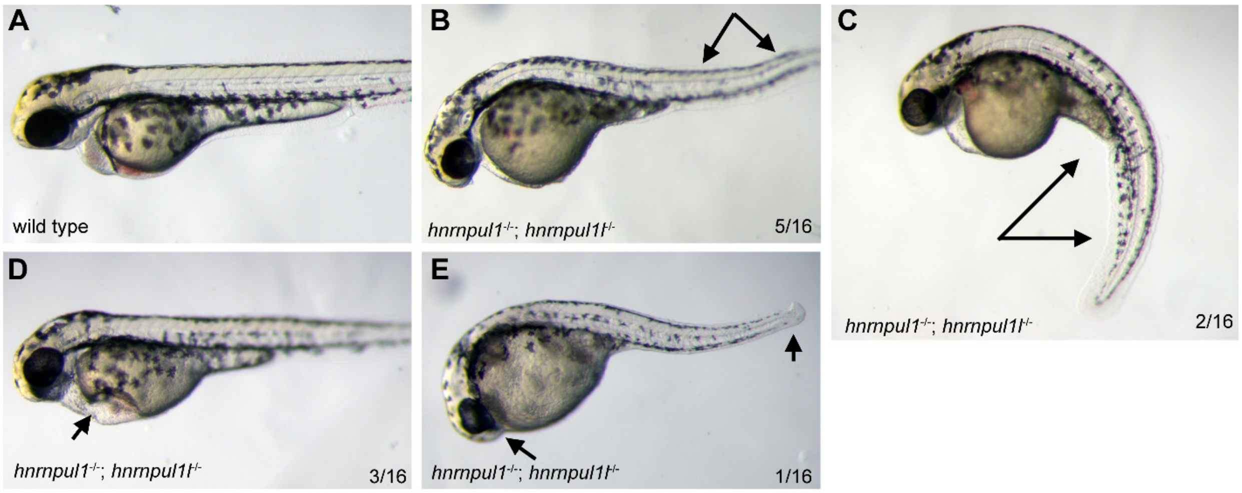

Figure S3– Low frequency severe developmental phenotypes in *hnrnpul1/1l* mutants at 48hpf

Gross morphology of wild type (A) and *hnrnpul1*$^{-/-}$;*hnrnpul1l*$^{-/-}$ double mutants at 48hpf (B-E). While the majority of embryos are viable and survived to adulthood, a minority of embryos show developmental phenotypes including B) Dorsal curvature C) Ventral curvature D) Edema E) Missing heart and caudal finfold. Arrows highlight developmental defect.

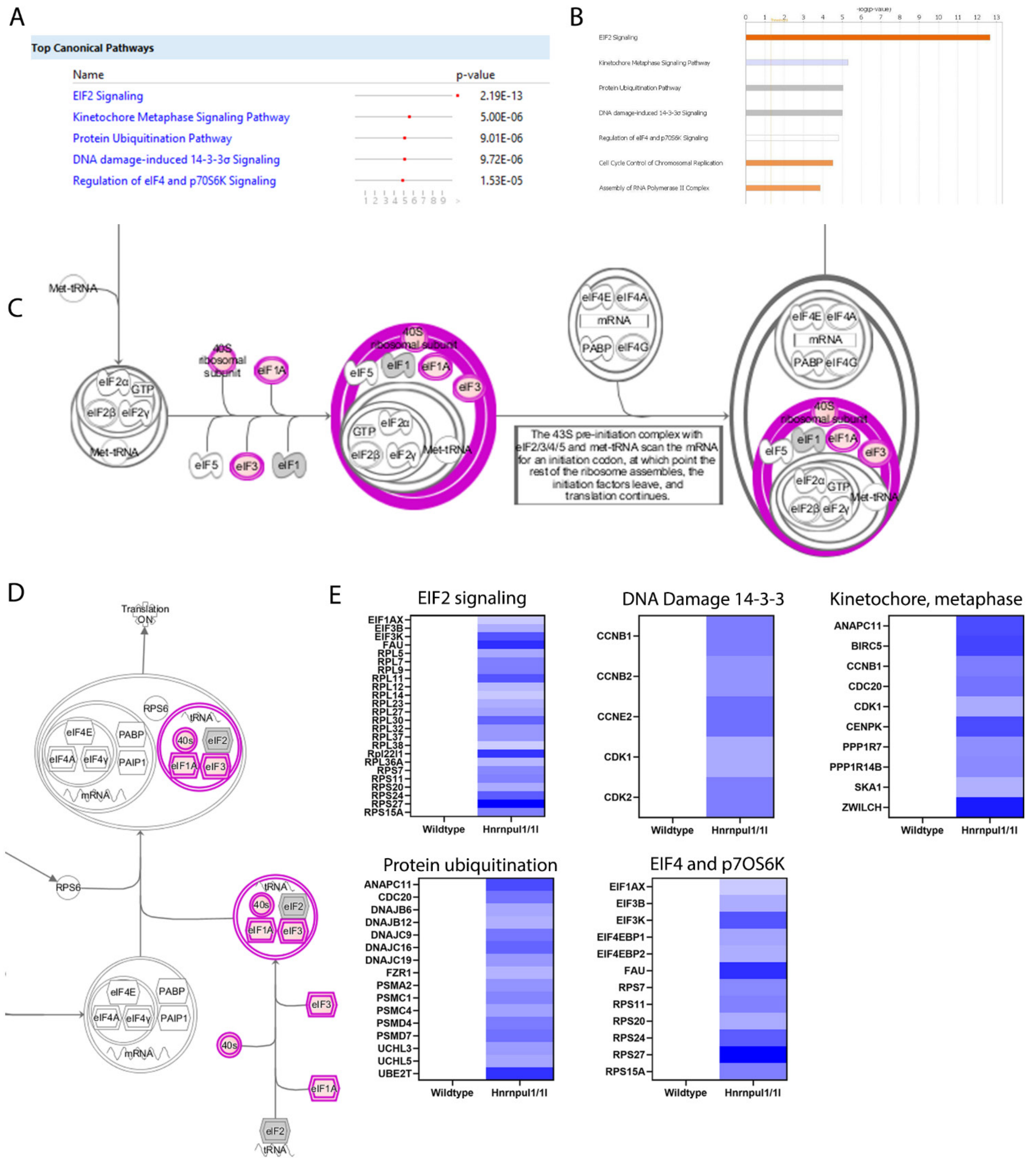

Figure S4–*hnrnpul1/1l* mutants show expression changes in pathways controlling fundamental cell processes by Ingenuity Pathway Analysis A) The top 5 pathways identified as disrupted in*hnrnpul1*$^{-/-}$;*hnrnpul1l*$^{-/-}$ double mutants. B) Graphical representation of most highly changed pathways. C) Pathway steps highlighted in purple mark the steps/genes of the EIF2 translational processes disrupted in *hnrnpul1*$^{-/-}$;*hnrnpul1l*$^{-/-}$ double mutants. D) Purple highlights disruptions in gene expression of components of the EIF2 and p70S6K pathway in *hnrnpul1*$^{-/-}$;*hnrnpul1l*$^{-/-}$ double mutants.

E) Heatmaps showing upregulation of genes in the marked pathways (Baseline in wild type is white (0), and increased expression over baseline is color coded in increasingly dark blue with 2-fold being darkest blue).

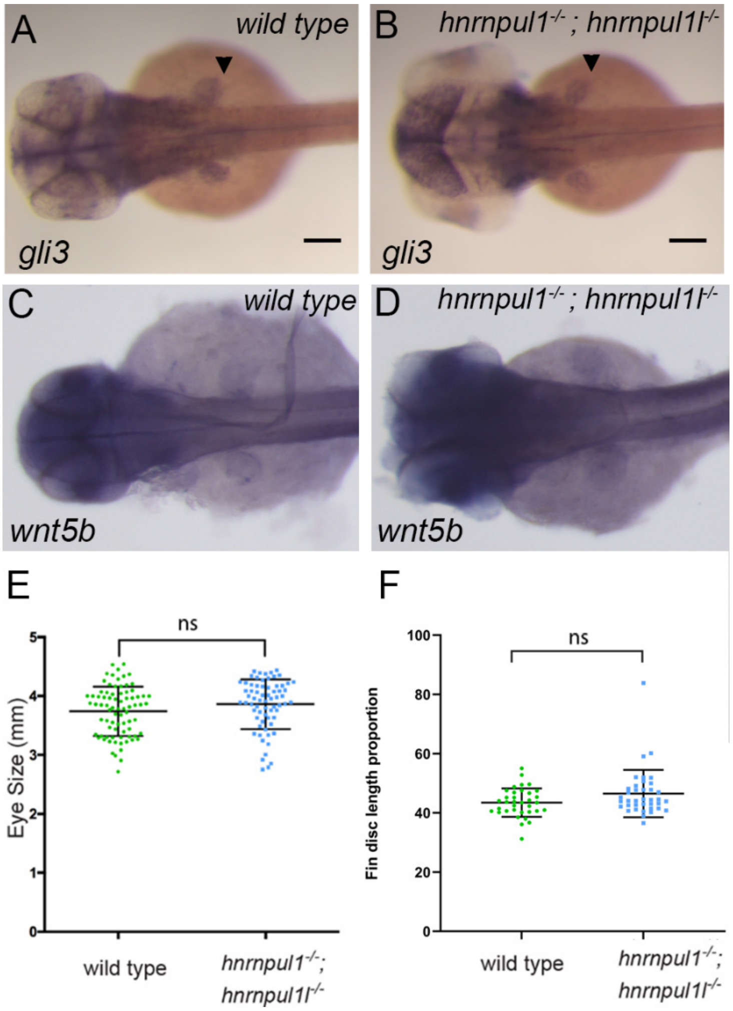

Figure S5: *hnrnpu1/1l* double mutant fins are specified correctly; Eye size nor fin disc to fin length ratio are not changed in hnrnpul1/1l mutants despite body growth defects.

A-D) mRNA expression of fin markers *gli3* (A-B) and *wnt5b* (C-D) at 48 hpf in wild type (A, C) and *hnrnpul1*$^{-/-}$;*hnrnpul1l*$^{-/-}$ double mutant (B, D) embryos shows no difference in expression. Scale bars = 100 µm. E) Eye size was measured from the indicated genotypes from 8 dpf larvae in the longest dimension. There is no statistical difference (ns) in eye size between *hnrnpul1*$^{-/-}$;*hnrnpul1l*$^{-/-}$ double mutant and wildtype (n=87 wildtype and 68 mutant eyes). F) The ratio of fin disc length to fin total length was measured from 16 dpf larvae (n=35 wildtype and 38 mutant fish). There is no statistically significant difference between *hnrnpul1*$^{-/-}$;*hnrnpul1l*$^{-/-}$ double mutant and wildtype as determined by Student's t-test.

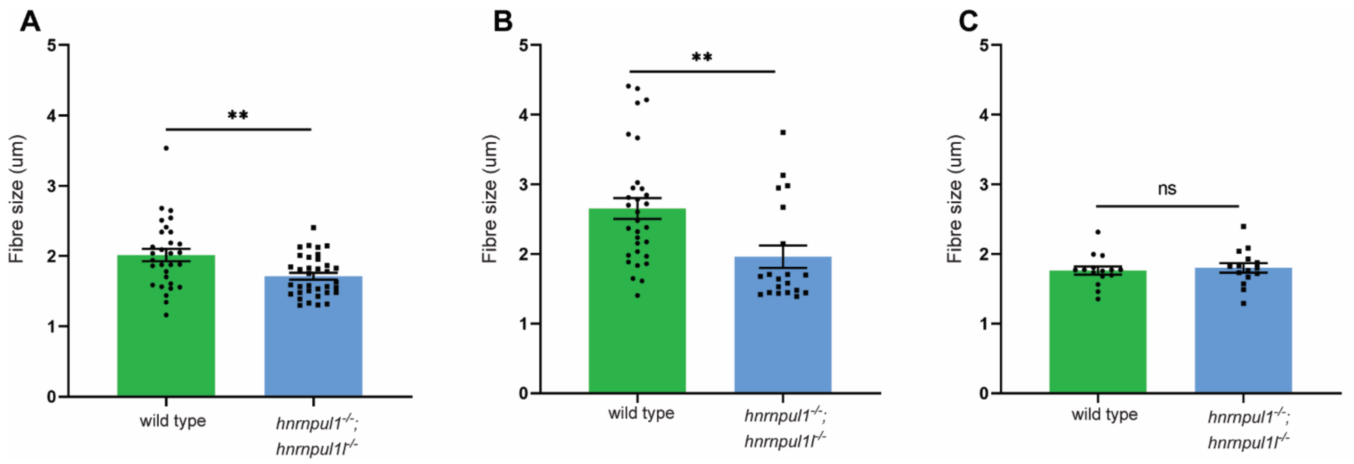

Figure S6- Muscle Fiber size is decreased in *hnrnpul1/1l* mutant fins and pharyngeal muscle

Average width of single MF20 stained muscle fibers at 72 hpf in a) Fin (n= 31 wildtype and 20 *hnrnpul1/1l* fibres), B) Pharyngeal muscle (n= 30 wildtype and 35 *hnrnpul1/1l* fibres) and C) Trunk (n= 15 wildtype and 15 *hnrnpul1/1l* fibers). **=p<0.01; ns= not significantly different as determined by Student's t-test.

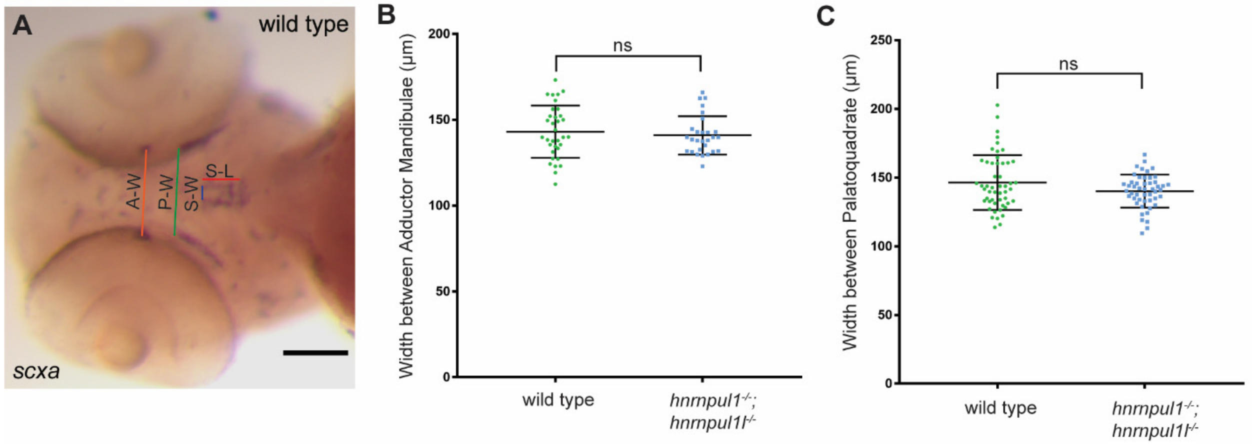

Figure S7 – *hnrnpu1/1l* mutants do not show differences in the width between the Palatoquadrate or Adductor Mandibulae tendons.

A) WISH staining for *scleraxis* (*scxa*) in the Sternohyoideus, Palatoquadrate and Adductor Mandibulae tendons in a wild type embryo at 72hpf. Coloured lines demonstrate location of tendon measurements. A-W = width between Adductor Mandibulae tendons, P-W = width between Palatoquadrate tendons, S-W= width between Sternohyoideus tendons, S-L = Sternohyoideus length. B) Quantification of the width between Adductor Mandibulae tendons in wild type (n=34) and *hnrnpul1*$^{-/-}$; *hnrnpul1l*$^{-/-}$ double mutant (n=28) embryos. C) Quantification of the width between Palatoquatrade tendons in wild type (n=51) and *hnrnpul1*$^{-/-}$; *hnrnpul1l*$^{-/-}$ double mutant (n=49) embryos. ns = not significant, determined by Student T-test.

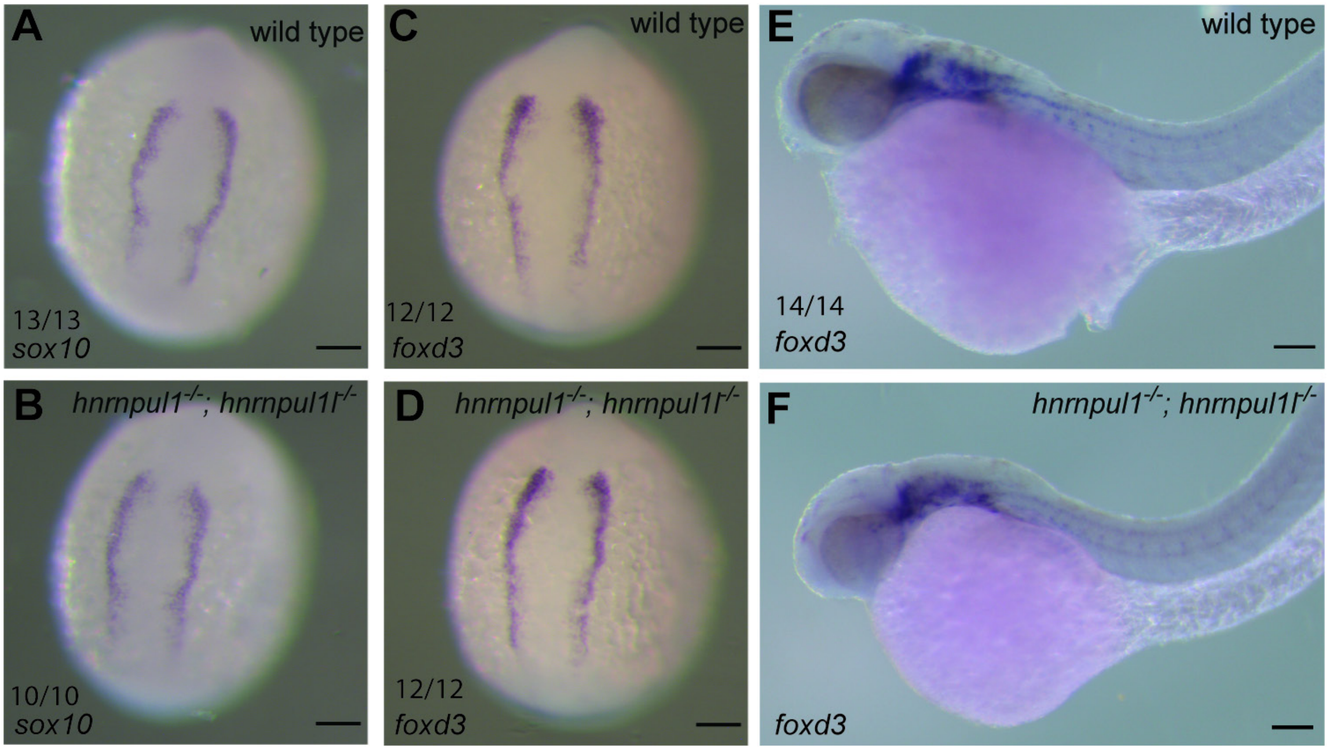

Figure S8 – Neural crest gene expression is not affected by *hnrpul1/1l* mutations

A,B) WISH staining for *sox10* in wildtype (A) and *hnrnpul1*$^{-/-}$; *hnrnpul1l*$^{-/-}$ double mutant (B) embryos at 12hpf. C-F) WISH staining for *foxd3* in wildtype (C, E) and *hnrnpul1*$^{-/-}$; *hnrnpul1l*$^{-/-}$ double mutants (D, F) embryos at 12hpf (C,D), or at 32 hpf (E, F). Scale bars = 100µm.

Table S3 – Non-causative variants identified in patients with limb anomalies.

Gene	Variant	Reason for exclusion
Podocalyxin-like gene *POXDL*	Chr7(GRCh37):g.131195974C>T, NM_001018111.2(PODXL): c.319G>A, p.(Val107Met)	• A biallelic loss of function variant in this gene was reported in an autosomal recessive juvenile Parkinson family (1)<br>• Homozygous knockout in mouse results in perinatal lethality due to severe defects in kidney development and omphalocele. Limb development is normal in these embryos (2)
protein kinase D2 gene *PRKD2*	Chr19(GRCh37):g.47204104G>A, NM_016457.4(PRKD2): c.1073C>T, p.(Ala358Val)	• *in silico* analysis (SIFT, PolyPhen2, alignGVGD, MutationTaster) did not predict any damaging effect on the protein and either heterozygous or homozygous loss of PRKD2 in Rhesus monkey and mouse, respectively, leads to hyperinsulinemia and insulin resistance without any reported congenital limb anomalies (3).
cystic fibrosis transmembrane conductance regulator gene *CFTR*	Chr7(GRCh37):g.117232214A>T, NM_000492.3(CFTR): c.1993A>T, p.(Thr665Ser)	• This variant has been reported as disease-associated in the literature where is has been found in the heterozygous state in two clinically affected individuals of Tunisian and Egyptian descent (4,5)<br>• *In vitro* functional studies suggest potentially decreased chloride currents (6) and a splice enhancer effects leading to partial exclusion of coding sequence (7).<br>• This finding was unexpected as neither affected individual presented with clinical features of cystic fibrosis, although this variant may be associated with a mild presentation. Given the paucity of clinical information on the impact of this variant in the literature, it is impossible to determine if homozygosity for this variant has any clinical impact. Nevertheless given the well studied nature of pathogenic variation in *CFTR* in humans, this gene is not a plausible candidate for the striking developmental anomalies in these siblings.

Table S4 – Primers used for CRISPR mutagenesis, genotyping, production of WISH probes and qPCR.

Gene Name	Forward Primer	Reverse Primer	Product Size	Use
hnrnpul1l	TAATACGACTCACTATAGCGAACTGATGAGGAAGGGAGTTTTAGAGCTAGAAATAGCAAG	N/A	N/A	sgRNA
hnrnpul1l	AAAGCGAACTGATGAGGAAGGTCATGGCGTTTAAACCTTAATTAAGCTGTTGTAGGGAAGGATGTTCCCGATCAT	N/A	N/A	STOP cassette
hnrnpul1	TAATACGACTCACTATAGGTGTAAGCAAGCTGAGGATGTTTTAGAGCTAGAAATAGCAAG	N/A	N/A	sgRNA
hnrnpul1	GAAGGTGTAAGCAAGCTGAGGTCATGGCGTTTAAACCTTAATTAAGCTGTTGTAGGATGGGAAAGATGTGCCCGA	N/A	N/A	STOP cassette
hnrnpul1l	AGGTTTTCAACGCAAGGCTA	GGGTGGGTTCTTCCAAGTCT	WT= 238bp,<br>Ca52= 344bp	Genotyping
hnrnpul1	GGTTTCACCGTAAGGCTGTT	TTTGTAGCATCCCATTTTTCA	WT= 246 bp<br>Ca53= 281bp<br>Ca54= 309bp	Genotyping
hnrnpul1	AACAGCCCACCTGTGATGAG	GCTCACTGCTGGGAAGATGT	672bp	WISH
hnrnpul1l	TCTTCCAGGAGCAGAAAAAGCA	GCACCTGCCCACAAATAAGC	538bp	WISH
hnrnpul1-12f1-14r1	GGCCACTCAGTGTCTGAACC	GTCTCTCTGCAGCTCGATGAA	396 bp	RT-PCR
hnrnpul1-12f2-14r2	ACCGACTACTTCAGATCGCC	AGTCTCGAGCTTCGTCTCTCT	400 bp	RT-PCR
hnrnpul1l-12f- 14r	GGCCACACAGTGTCTCAACA	TGAGCCTCCATCCCTGTAGT	495 bp	RT-PCR
scleraxis	ACAGAGACAGAAAGCCGGAGGAGT	CTTACCATTTTCCTCTGGTTGCTGAG	850bp	WISH
hand2	TCGCTGTCATGAAGAACCCC	GGCCAACCAGTTCTCCCTTT	513bp	WISH
tbx5	AAACTCTCCCAGTGACAGCG	TGTGTGTTCGTGGTAGGAGC	913bp	WISH
col1a1a	AAGGAGGGCCAGAAAGGTA	AGGGTGGTGTCAACCTCAAG	995bp	WISH

	A			
gli3	(8)			WISH
sox10	ACCGTGACACACTCTACCAAGATGACC	TAATACGACTCACTATAGGCATGATAAAATTTGCACCCTGAAAAGG	935bp	WISH
foxd3	CGGCATTGGGAATCCATA	TAATACGACTCACTATAGGCAACGAAATGAAATAGAAAGAAGGA	684bp	WISH
wnt5b	GGATTACTGCCTGCGCAATG	**TGTAATACGACTCACTATAC**AGCTCTGACATCAGCAAGGT	659 bp	WISH
hnrnpul1	AGACGTCAGCTTGGAGAACA	CAGCATGTTTAGCAGCCCAT	125bp, exon 9-10	qPCR
hnrnpul1l	AAATCACGGCAGATCCCAGA	CTTCACATCATAACGCCCGG	75bp, exon 3-4	qPCR

Supplementary methods:

Genotyping *hnrnpul1/1l* mutants

Genomic DNA was prepared by exposing tissue to 25mM NaOH at 55°C for 30 mins (50µl for adult fin clips and whole embryos, 25µl for embryo tails). The solution was neutralised with an equal volume of 40 mM Tris HCl pH=5. Samples were vortexed and centrifuged, stored at -20°C until required for PCR. Final PCR reaction consisted of 2 µl 5X Phusion HF buffer (New England Biolabs, Massachusetts – B0518S), 0.2 µl 10 mM dNTPs (ThermoFisher), 0.5 µl 50 µM forward primer, 0.5 µl 50 µM reverse primer (Table S4), 0.1 µl Phusion polymerase (NEB – M0530), 5.7 µl H_2O and 1 µl gDNA. PCR reactions were subject to the following conditions 98°C for 30 secs; 98°C for 10 secs, 62°C for 20 secs, 72°C for 30 secs (35 cycles); 72°C for 3 mins. PCR products were visualised on 2% agarose electrophoresis gel, expected product sizes are detailed in Table S4.

References

1. Sudhaman, S., Prasad, K., Behari, M., et al. (2016) Discovery of a frameshift mutation in podocalyxinlike (PODXL) gene, coding for a neural adhesion molecule, as causal for autosomal-recessive juvenile Parkinsonism. *J. Med. Genet.*, **53**, 450–456.
2. Doyonnas, R., Kershaw, D. B., Duhme, C., et al. (2001) Anuria, omphalocele, and perinatal lethality in mice lacking the CD34-related protein podocalyxin. *J. Exp. Med.*, **194**, 13–27.
3. Xiao, Y., Wang, C., Chen, J. Y., et al. (2018) Deficiency of PRKD2 triggers hyperinsulinemia and metabolic disorders. *Nat. Commun.*, **9**, 1–11.
4. Messaond, T., Verlingue, C., Denamur, E., et al. (1996) Distribution of CFTR mutations in cystic fibrosis patients of Tunisian origin: Identification of two novel mutations. *Eur. J. Hum. Genet.*, **4**, 20–24.
5. Naguib, M. L., Schrijver, I., Gardner, P., et al. (2007) Cystic fibrosis detection in high-risk Egyptian children and CFTR mutation analysis. *J. Cyst. Fibros.*, **6**, 111–116.
6. Vankeerberghen, A., Wei, L., Jaspers, M., et al. (1998) Characterization of 19 disease-associated missense mutations in the regulatory domain of the cystic fibrosis transmembrane conductance regulator. *Hum. Mol. Genet.*, **7**, 1761–1769.
7. Aznarez, I., Chan, E. M., Zielenski, J., et al. (2003) Characterization of disease-associated mutations affecting an exonic splicing enhancer and two cryptic splice sites in exon 13 of the cystic fibrosis transmembrane conductance regulator gene. *Hum. Mol. Genet.*, **12**, 2031–2040.
8. Tyurina, O. V., Guner, B., Popova, E., et al. (2005) Zebrafish Gli3 functions as both an activator and a repressor in Hedgehog signaling. *Dev. Biol.*, **277**, 537–556.
